# Supplementary material for: Deuteration promotes circularly polarized light emission by suppression of vibration
Source: Nat Commun. 2025 Dec 18;17:678. doi: 10.1038/s41467-025-67342-y (PMC12820309; doi:10.1038/s41467-025-67342-y)
Supplement: Supplementary file 1 — Supplementary Information [file 41467_2025_67342_MOESM1_ESM.pdf]

Supplementary Information

Deuteration promotes circularly polarized light emission by suppression of vibration

Zhanxiang Chen[1], Manli Huang[1], Cheng Zhong[2], Mengcheng Wang[1], Jingsheng Miao[1], Chuluo Yang[1]*

[1]Shenzhen Key Laboratory of New Display and Storage Materials, College of Materials Science and Engineering, Shenzhen University, Shenzhen, P. R. China

[2]Hubei Key Lab on Organic and Polymeric Optoelectronic Materials, Department of Chemistry, Wuhan University, Wuhan, P. R. China

*Corresponding author. Email: Chuluo Yang (clyang@szu.edu.cn)

Table of Contents

Supplementary Figures

Supplementary Fig. 1. ^{1}H NMR spectrum of R/S-D(16)CzCN in $CDCl_3$ at room temperature

Supplementary Fig. 2. ^{13}C NMR spectrum of R/S-D(16)CzCN in $CDCl_3$ at room temperature

Supplementary Fig. 3. HRMS spectrum of R/S-D(16)CzCN

Supplementary Fig. 4. ^{1}H NMR spectrum of R/S-MeCzCN in $CDCl_3$ at room temperature

Supplementary Fig. 5. ^{13}C NMR spectrum of R/S-MeCzCN in $CDCl_3$ at room temperature

Supplementary Fig. 6. HRMS spectrum of R/S-MeCzCN

Supplementary Fig. 7. Single crystal structures of R/S-D(16)CzCN

Supplementary Fig. 8. Single crystal structures of R/S-MeCzCN

Supplementary Fig. 9. Single-crystal X-ray diffraction analysis of R/S-D(16)CzCN

Supplementary Fig. 10. TGA curves of R/S-MeCzCN, R/S-CzCN, and R/S-D(16)CzCN with a heating rate of 10 $^{o}C \cdot min^{-1}$ under nitrogen atmosphere

Supplementary Fig. 11. Photophysical and chiroptical properties of R/S-MeCzCN, R/S-CzCN, and R/S-D(16)CzCN in different solvents (1×10^{-5} M) at 300 K

Supplementary Fig. 12. Transient decay of R/S-MeCzCN, R/S-CzCN, and R/S-D(16)CzCN neat films at room temperature

Supplementary Fig. 13. Vector displacement diagram of the normal mode with Huang-Rhys factor exceeding 0.2

Supplementary Fig. 14. Vector displacement diagram of the high-frequency modes

Supplementary Fig. 15. Simulated CD spectra at the CAM-B3LYP/def2-SVP level based on optimized S_0 geometries

Supplementary Fig. 16. g_{PL} values versus wavelength curves of deuterated R/S-D(16)CzCN and non-deuterated R/S-CzCN as chiral emitters

Supplementary Fig. 17. ΔI and DC values versus wavelength curves of R/S-CzCN and R/S-D(16)CzCN

Supplementary Fig. 18. Atomic and fragment contributions to **m**

Supplementary Fig. 19. Calculated orientation distributions and angles (θ, in units of deg) of the **μ** (red) and **m** (blue) and theoretically calculated *g*-factors of stationary-point structures: R-MeCzCN; R-CzCN; R-D(16)CzCN

Supplementary Fig. 20. $S_1 \rightarrow S_0$ transition density distributions of R-MeCzCN, R-CzCN and R-D(16)CzCN calculated from stationary-point TDDFT calculations

Supplementary Fig. 21. Vector displacement diagram of the normal mode with a frequency of about 1,360 cm^{-1}

Supplementary Fig. 22. Perturbational effect on the exciton wavefunction (transition density) of the S_1 state along the coordinate of representative vibrational modes

Supplementary Fig. 23. Theoretically calculated **m** versus vibrational frequency

Supplementary Fig. 24. Chemical structures of the comprising materials in CP-OLEDs

Supplementary Fig. 25. Conventional CP-OLEDs based on R/S-chiral emitters

Supplementary Fig. 26. Tandem CP-OLEDs based on R/S-CzCN

Supplementary Fig. 27. BN2 enantiomers and diastereomeric interactions with the chiral host

Supplementary Fig. 28. Chiroptical properties of R/S-D(16)CzCN:BN2

Supplementary Fig. 29. Diastereomeric interactions and chiroptical properties of R/S-D(16)CzCN:DtBuCzB

Supplementary Fig. 30. Diastereomeric interactions and chiroptical properties of R/S-D(16)CzCN:BNSeSe

Supplementary Fig. 31. Förster resonance energy transfer

Supplementary Fig. 32. g_{PL} values versus wavelength curves of deuterated R/S-D(16)CzCN and non-deuterated R/S-CzCN as chiral hosts

Supplementary Fig. 33. CP-OLEDs based on R/S-D(16)CzCN:DtBuCzB

Supplementary Fig. 34. CP-OLED s based on R/S-D(16)CzCN:BNSeSe

Supplementary Fig. 35. Circular polarization of CP-OLEDs based on R/S-D(16)CzCN:DtBuCzB and R/S-D(16)CzCN:BNSeSe

Supplementary Fig. 36. CP-OLEDs based on R-MeCzCN:BN2 and R-CzCN:BN2

Supplementary Fig. 37. CP-OLEDs based on S-MeCzCN:BN2 and S-CzCN:BN2

Supplementary Fig. 38. Circular polarization of CP-OLEDs based on R/S-CzCN:BN2 and R/S-MeCzCN:BN2

Supplementary Fig. 39. Operational lifetimes of the conventional CP-OLEDs based on R-D(16)CzCN:BN2, R-CzCN:BN2 and R-MeCzCN:BN2, measured at an initial luminance of 1,000 $cd \cdot m^{-2}$

Supplementary Fig. 40. ^1H NMR spectrum of (P,P)/(M,M)-D(32)CzTBCO in $CDCl_3$ at room

temperature

Supplementary Fig. 41. ^{13}C NMR spectrum of (P,P)/(M,M)-D(32)CzTBCO in $CDCl_3$ at room temperature

Supplementary Fig. 42. HRMS spectrum of (P,P)/(M,M)-D(32)CzTBCO

Supplementary Fig. 43. Photophysical and chiroptical properties of (P,P)/(M,M)-CzTBCO and (P,P)/(M,M)-D(32)CzTBCO in different solvents (1×10^{-5} M) at 300 K

Supplementary Fig. 44. CP-OLEDs based on (P,P)/(M,M)-D(32)CzTBCO

Supplementary Fig. 45. Δ*I* and DC values versus wavelength curves of (P,P)/(M,M)-CzTBCO and (P,P)/(M,M)-D(32)CzTBCO

Supplementary Fig. 46. CP-OLEDs based on (P,P)/(M,M)-CzTBCO

Supplementary Fig. 47. Chemical structures of representative organic chiral emitters

Supplementary Fig. 48. HPLC chromatograms of CzCN

Supplementary Fig. 49. HPLC chromatograms of CzTBCO

Supplementary Fig. 50. HPLC chromatograms of D(16)CzCN

Supplementary Fig. 51. HPLC chromatograms of MeCzCN

Supplementary Fig. 52. HPLC chromatograms of D(32)CzTBCO

Supplementary Tables

Supplementary Table 1. Absolute *Φ* values of R/S-CzCN neat films measured in air and under vacuum conditions at room temperature with different excitation wavelengths

Supplementary Table 2. Physical data and kinetic parameters of R/S-D(16)CzCN, R/S-CzCN and R/S-MeCzCN in undoped film

Supplementary Table 3. Energy levels of the S_1 and S_2 states, S_1-S_2 splitting and excitonic coupling strength for R-D(16)CzCN, and R-CzCN

Supplementary Table 4. Maximum and average g_{PL} values for R/S-MeCzCN, R/S-CzCN, and R/S-D(16)CzCN across various solvents, measured within the 430-580 nm spectral range

Supplementary Table 5. Comparison of bond lengths, bond angles, and dihedral angles between R-CzCN and R-D(16)CzCN

Supplementary Table 6. Atomic coordinates of R-MeCzCN

Supplementary Table 7. Atomic coordinates of R-CzCN

Supplementary Table 8. Atomic coordinates of R-D(16)CzCN

Supplementary Table 9. Features of the reported CP-OLEDs

Supplementary Table 10. Maximum and average g_{PL} values for (P,P)/(M,M)-CzTBCO, and (P,P)/(M,M)-D(32)CzTBCO across various solvents, measured within the 480-600 nm spectral range

Supplementary Table 11. Summary of the calculated data for absolute configuration (*R* or *P*)

Supplementary Table 12. The specific rotation of new chiral compounds

Supplementary Notes

Supplementary Note 1. Orbital contribution to the magnetic transition dipole moment

Supplementary Note 2. Analyses of rate constants

Supplementary Note 3. Critical role of diastereomeric interactions

Supplementary Note 4. Mechanisms and rationale underlying CPL emission in CP-OLEDs based on R/S-D(16)CzCN as host

Supplementary References

Supplementary Figures

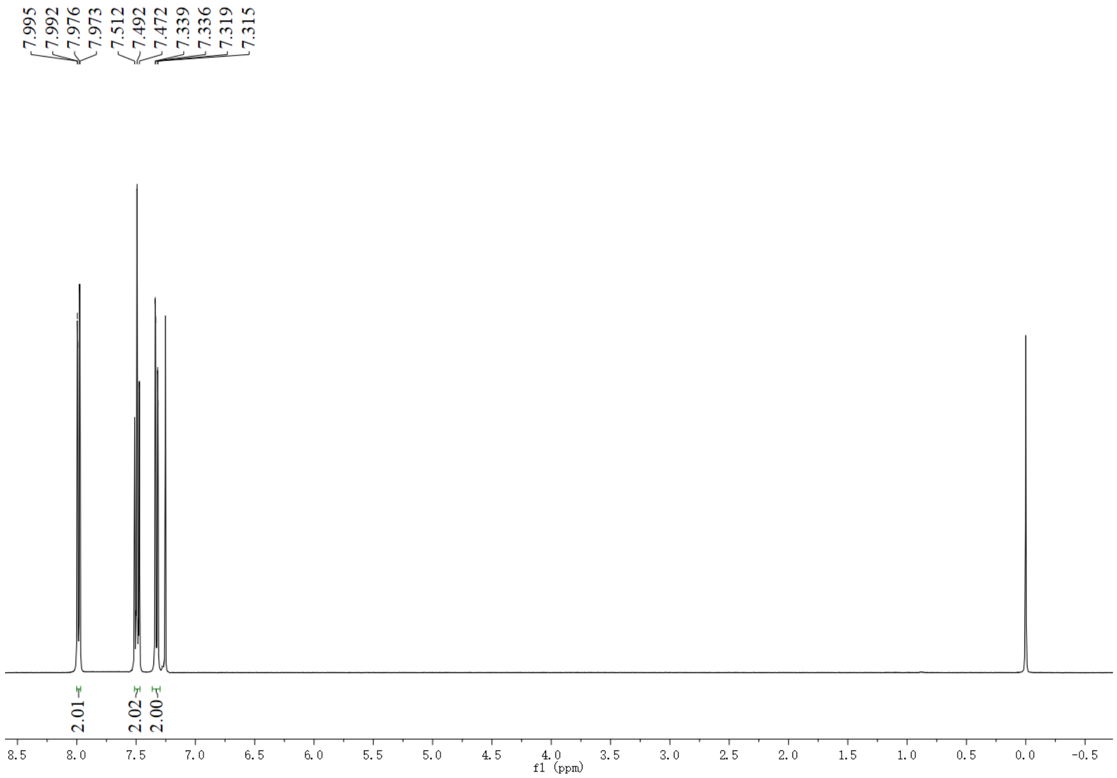

Supplementary Fig. 1. ^{1}H NMR spectrum of R/S-D(16)CzCN in $CDCl_3$ at room temperature.

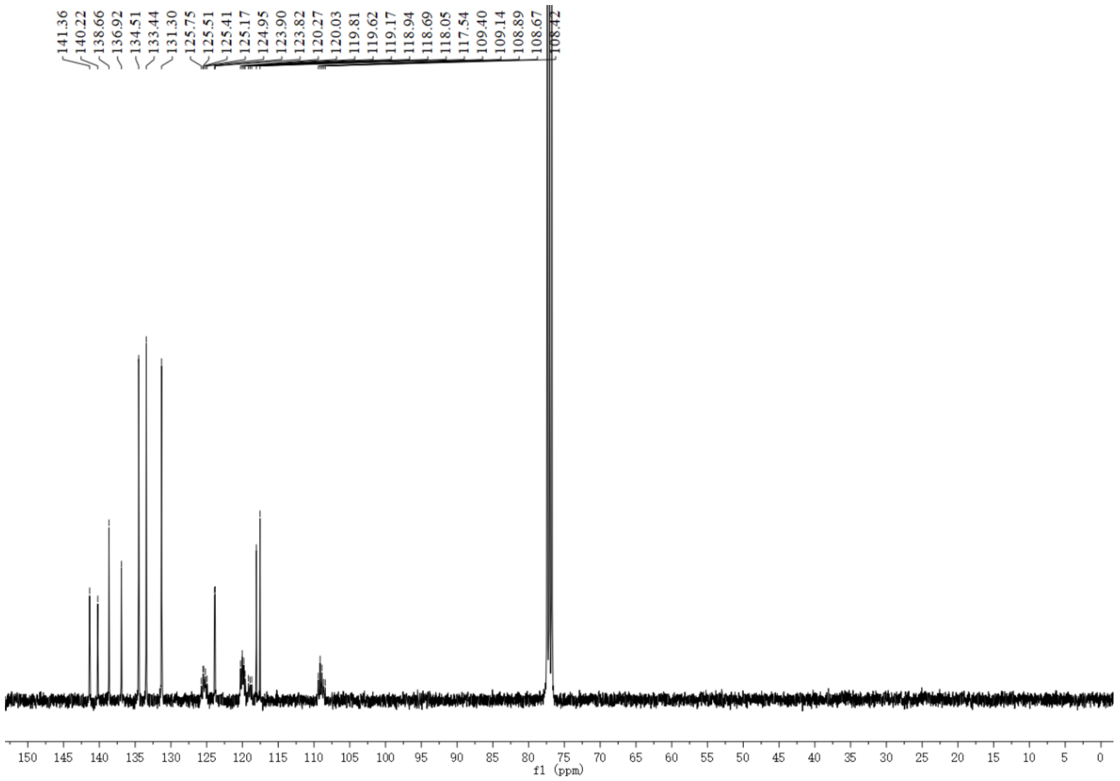

Supplementary Fig. 2. **^{13}C NMR spectrum of R/S-D(16)CzCN in $CDCl_3$ at room temperature.**

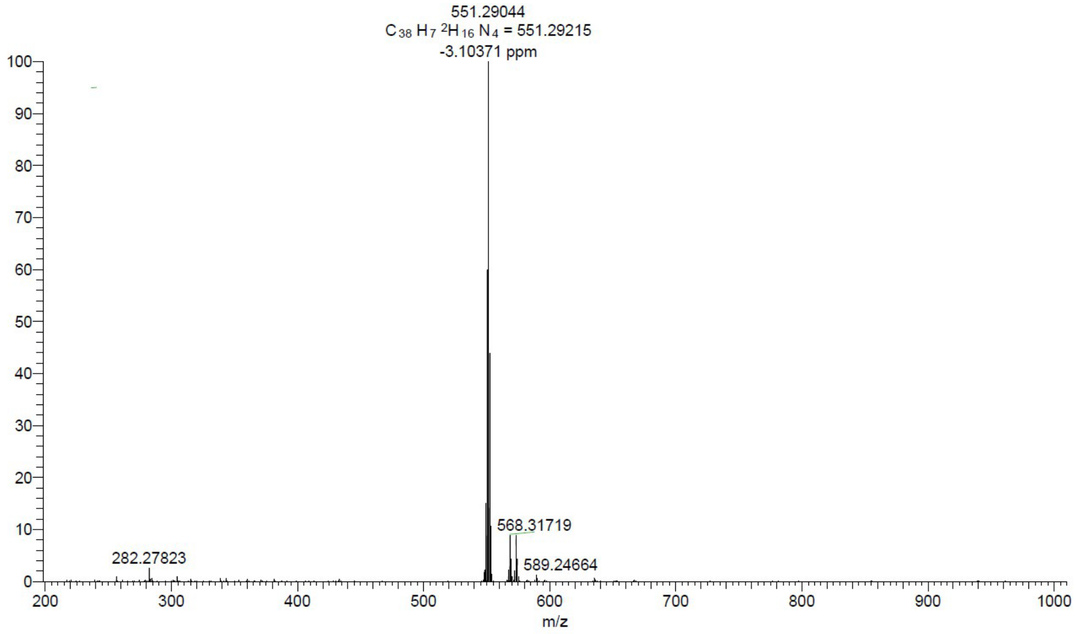

Supplementary Fig. 3. HRMS spectrum of R/S-D(16)CzCN.

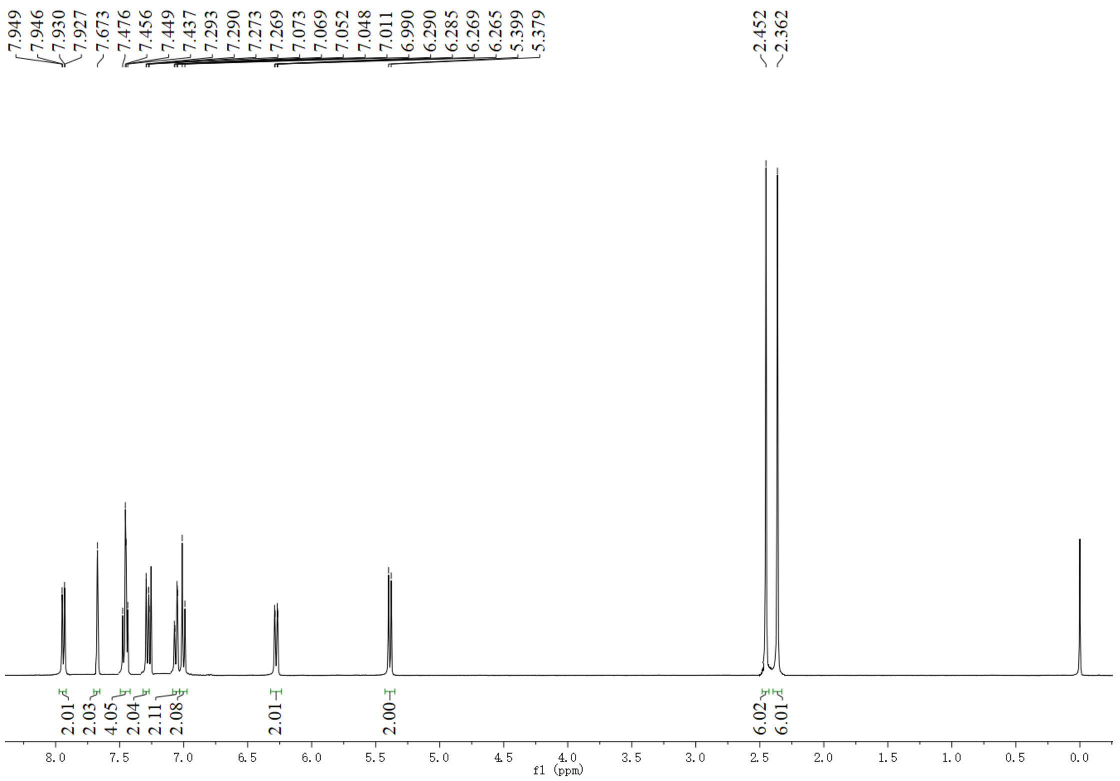

Supplementary Fig. 4. ^{1}H NMR spectrum of R/S-MeCzCN in $CDCl_3$ at room temperature.

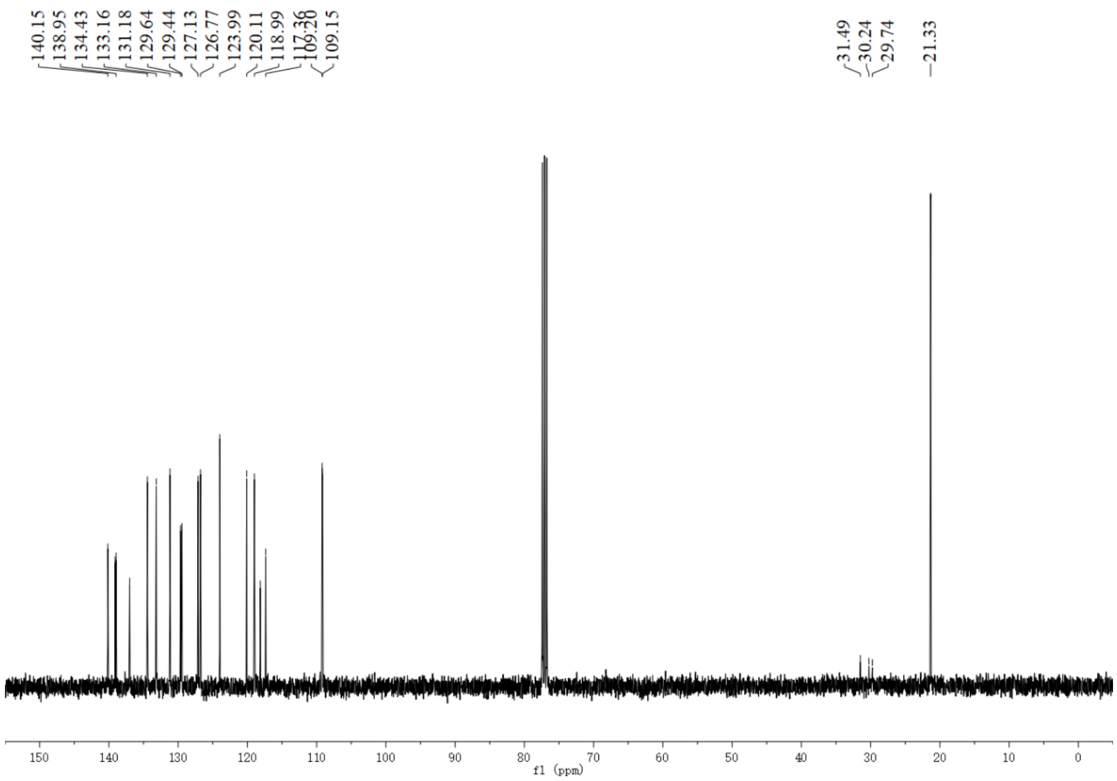

Supplementary Fig. 5. ^{13}C NMR spectrum of R/S-MeCzCN in $CDCl_3$ at room temperature.

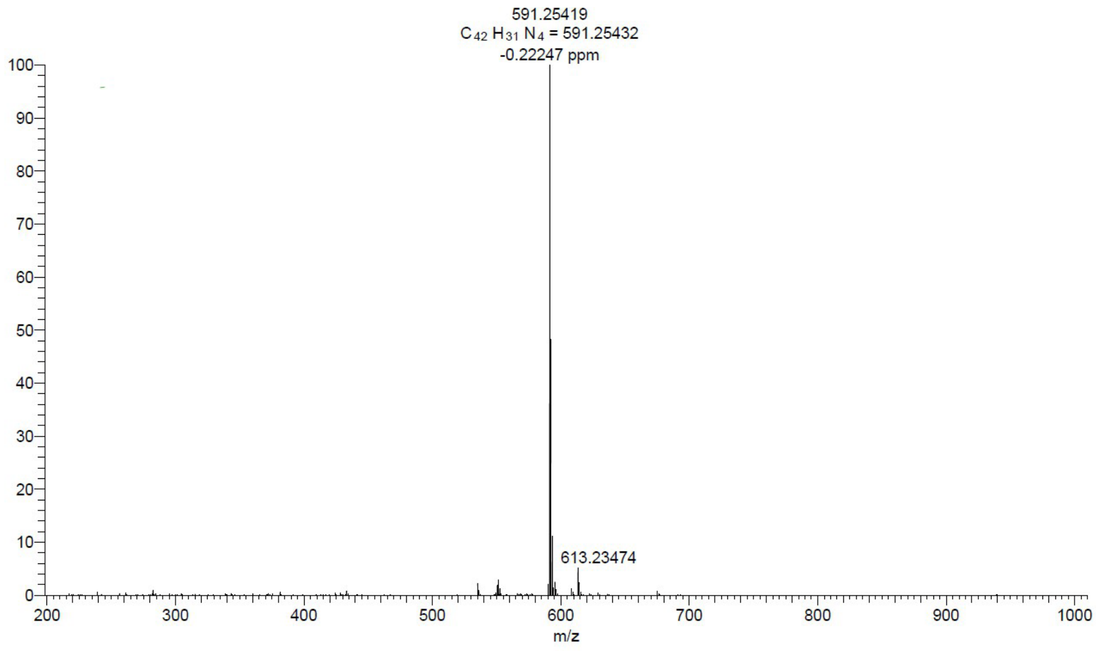

Supplementary Fig. 6. HRMS spectrum of R/S-MeCzCN.

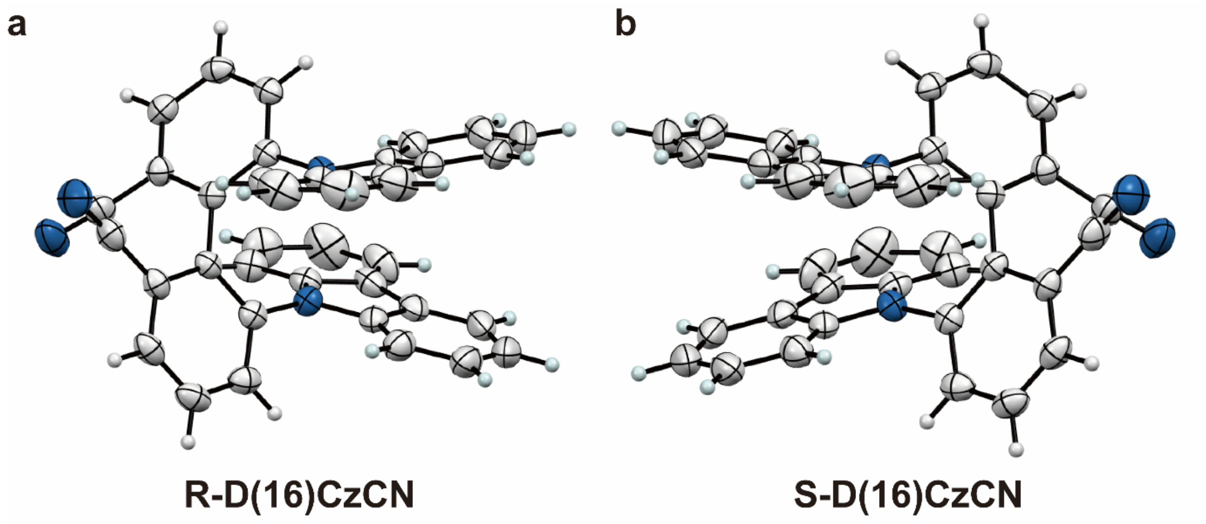

Supplementary Fig. 7. Single crystal structures of R/S-D(16)CzCN. (**a**) R-D(16)CzCN (CCDC 2267192). (**b**) S-D(16)CzCN (CCDC 2285327). The two crystals were grown by slow evaporating a solution in CH_2Cl_2:methanol (5:2) at 25°C. Selected bond length (Å) and angles (°): For R-D(16)CzCN: N1-C13 1.418(2) Å, N4-C21 1.422(3) Å, C14-C20 1.492(3) Å, ∠C7-N1-C13-C18 -42.9(3)°, ∠C27-N4-C21-C22 -52.3(3)°, ∠C13-C14-C20-C21 -62.1(3)°; For S-D(16)CzCN: N7-C14 1.418(2) Å, C26-N28 1.422(2) Å, C19-C21 1.492(2) Å, ∠C6-N7-C14-C15 42.8(2)°, ∠C29-N28-C26-C25 52.3(2)°, ∠C14-C19-C21-C26 62.3(2)°. For comparison, crystallographic data of R/S-CzCN (CCDC codes: 1945359 for R-CzCN, 1945360 for S-CzCN) were obtained from the Cambridge Crystallographic Data Centre. Selected bond length (Å) and angles (°): For R-CzCN: N1-C25 1.421 Å, N2-C32 1.421 Å, C30-C31 1.493 Å, ∠C12-N1-C25-C26 -42.8(2)°, ∠C37-N3-C21-C22 -52.1(7)°, ∠C25-C30-C31-C32 -62.2(4)°; For S-CzCN: N1-C25 1.421 Å, N2-C33 1.421 Å, C30-C32 1.493 Å, ∠C24-N2-C33-C34 42.8(8)°, ∠C1-N1-C25-C26 52.2(0)°, ∠C25-C30-C32-C33 62.2(1)°.

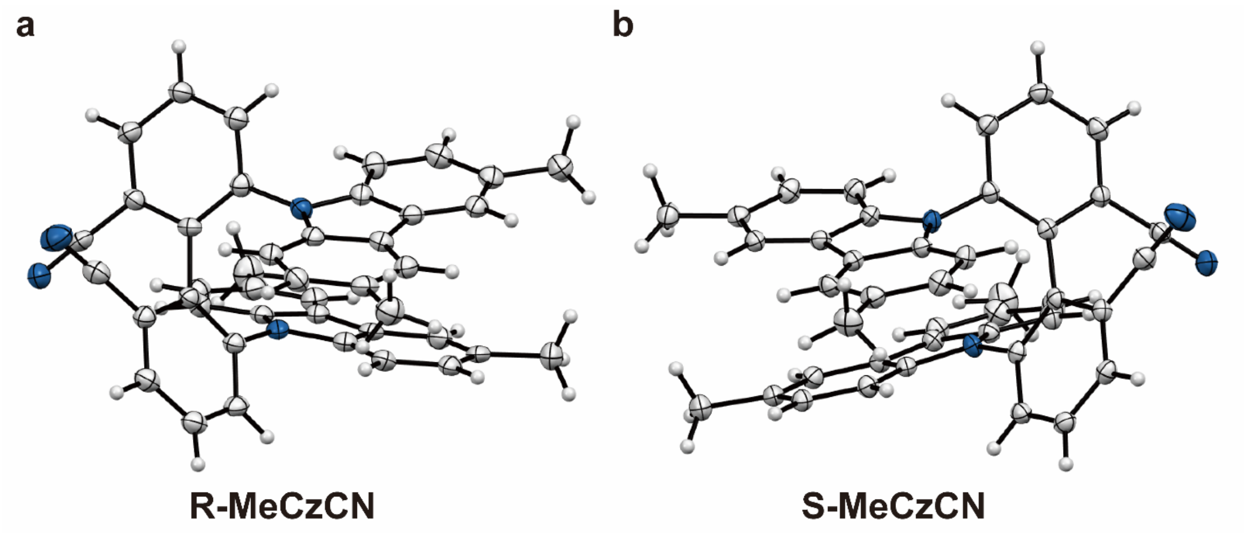

Supplementary Fig. 8. Single crystal structures of R/S-MeCzCN. (**a**) R-MeCzCN (CCDC 2280086). (**b**) S-MeCzCN (CCDC 2280087). The two crystals were grown by slow evaporating a solution in CH_2Cl_2:methanol:dioxane (2:2:1) at 25ºC. Selected bond length (Å) and angles (º): For R-MeCzCN: N1-C15 1.424(6) Å, N2-C28 1.412(6) Å, C21-C22 1.506(6) Å, ∠C1-N1-C15-C16 114.2(5)º, ∠C29-N2-C28-C27 -50.0(6)º, ∠C15-C21-C22-C28 -64.9(6)º; For S-MeCzCN: N1-C15 1.416(2) Å, N2-C26 1.425(2) Å, C20-C21 1.494(2) Å, ∠C7-N1-C15-C16 48.5(2)º, ∠C29-N2-C26-C25 52.0(2)º, ∠C15-C20-C21-C26 -65.0(2)º.

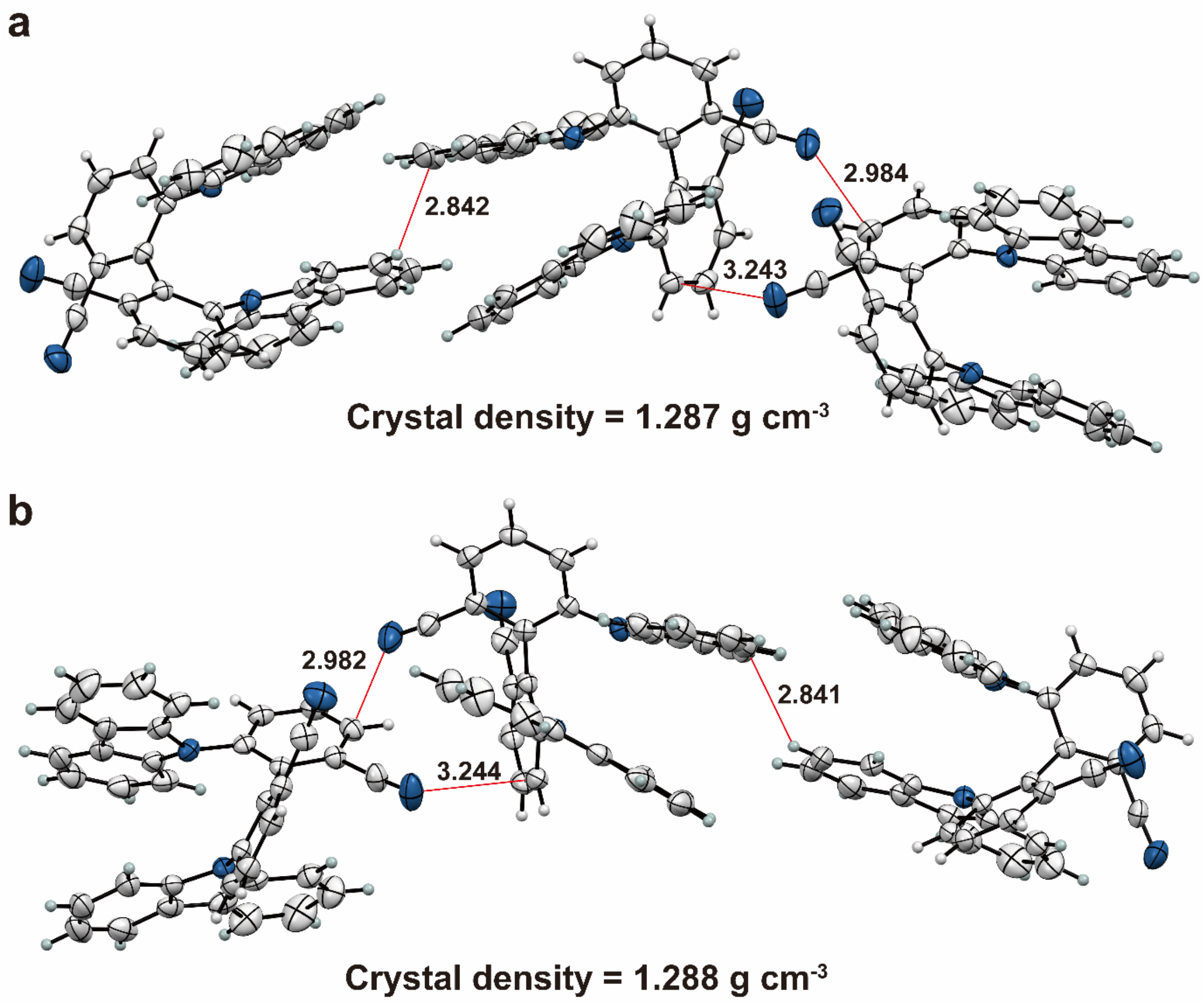

Supplementary Fig. 9. Single-crystal X-ray diffraction analysis of R/S-D(16)CzCN. (**a**) R-D(16)CzCN (CCDC 2267192). (**b**) S-D(16)CzCN (CCDC 2285327). Selected crystallographic data: For R-D(16)CzCN: C-D···π 2.842 Å, C≡N···π 2.984 Å, C≡N···π 3.243 Å, crystal density: 1.287 g cm^{-3}; For S-D(16)CzCN: C-D···π 2.841 Å, C≡N···π 2.982 Å, C≡N···π 3.244 Å, crystal density: 1.288 g cm^{-3}. For comparison, crystallographic data of R/S-CzCN (CCDC codes: 1945359 for R-CzCN, 1945360 for S-CzCN) were obtained from the Cambridge Crystallographic Data Centre. Selected crystallographic data: For R-CzCN, C-H···π 2.863 Å, C≡N···π 2.981 Å, C≡N···π 3.248 Å, crystal density: 1.284 g cm^{-3}; For S-CzCN, C-H···π 2.865 Å, C≡N···π 2.983 Å, C≡N···π 3.247 Å, crystal density: 1.284 g cm^{-3}.

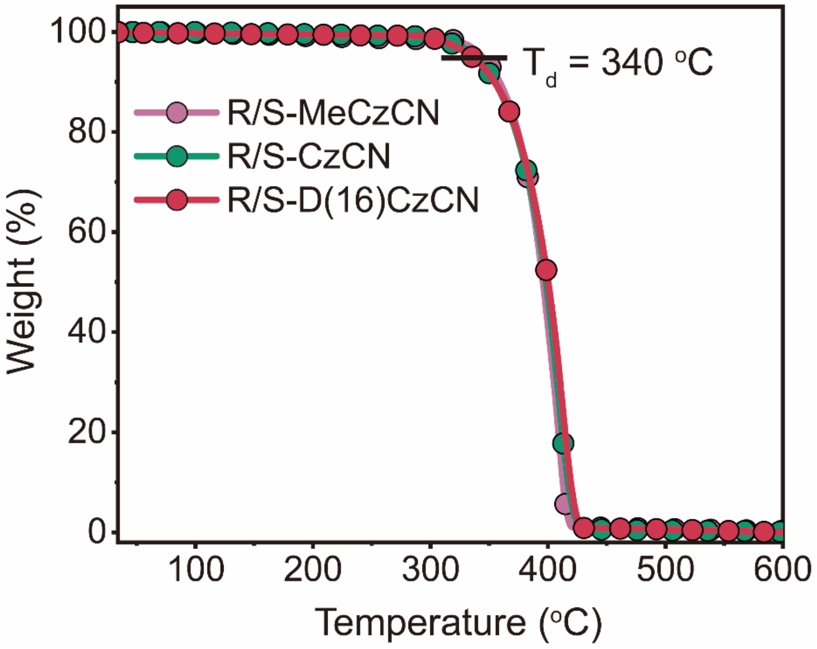

Supplementary Fig. 10. TGA curves of R/S-MeCzCN, R/S-CzCN, and R/S-D(16)CzCN with a heating rate of 10 °C·min^{-1} under nitrogen atmosphere.

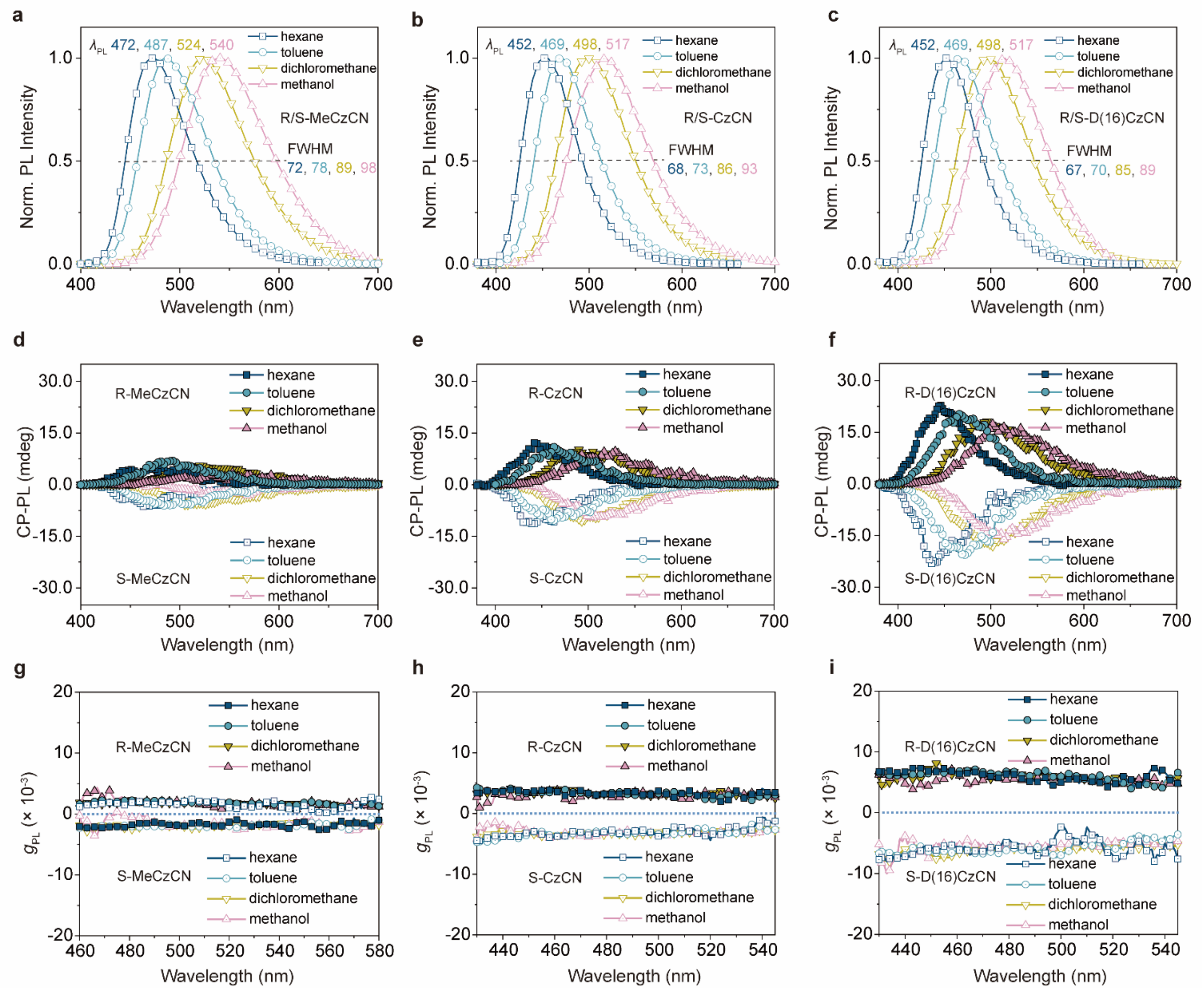

Supplementary Fig. 11. Photophysical and chiroptical properties of R/S-MeCzCN, R/S-CzCN, and R/S-D(16)CzCN in different solvents (1 × 10^{-5} M) at 300 K. (**a**) PL spectra of R/S-MeCzCN in different solvents (1 × 10^{-5} M) at 300 K. (**b**) PL spectra of R/S-CzCN in different solvents (1 × 10^{-5} M) at 300 K. (**c**) PL spectra of R/S-D(16)CzCN in different solvents (1 × 10^{-5} M) at 300 K. (**d**) CP-PL spectra of R/S-MeCzCN in different solvents (1 × 10^{-5} M) at 300 K. (**e**) CP-PL spectra of R/S-CzCN in different solvents (1 × 10^{-5} M) at 300 K. (**f**) CP-PL spectra of R/S-D(16)CzCN in different solvents (1 × 10^{-5} M) at 300 K. (**g**) g_{PL} values versus wavelength curves of R/S-MeCzCN in different solvents (1 × 10^{-5} M) at 300 K. (**h**) g_{PL} values versus wavelength curves of R/S-CzCN in different solvents (1 × 10^{-5} M) at 300 K. (**i**) g_{PL} values versus wavelength curves of R/S-D(16)CzCN in different solvents (1 × 10^{-5} M) at 300 K.

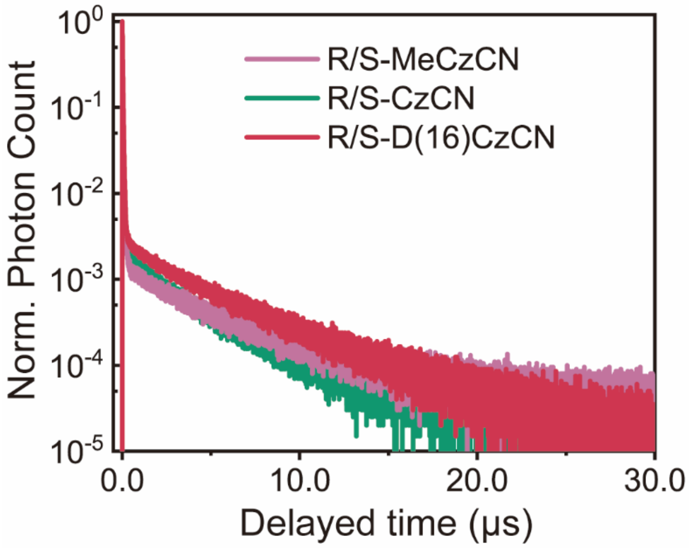

Supplementary Fig. 12. Transient decay of R/S-MeCzCN, R/S-CzCN, and R/S-D(16)CzCN neat films at room temperature.

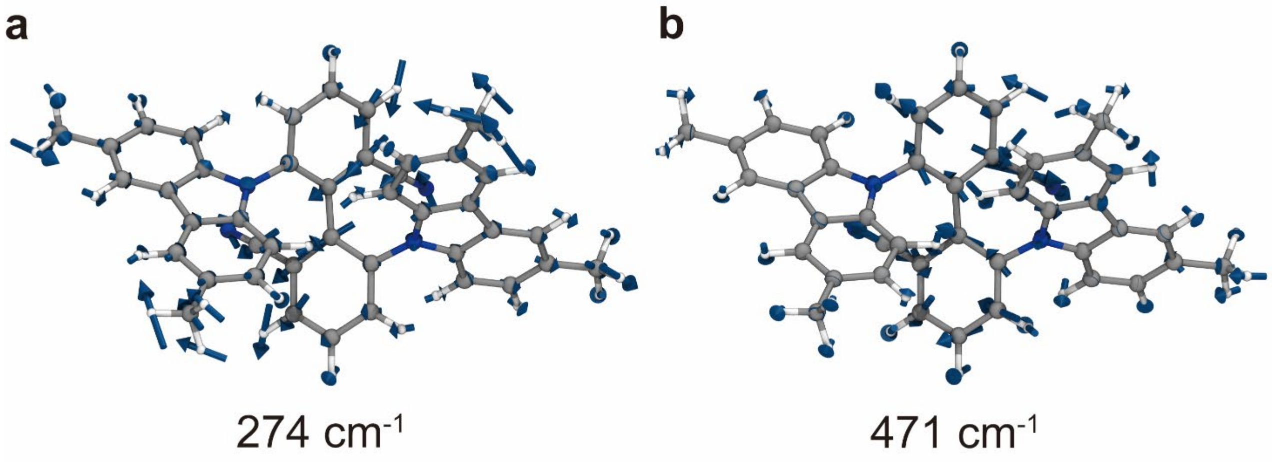

Supplementary Fig. 13. Vector displacement diagram of the normal mode with Huang-Rhys factor exceeding 0.2. (**a**) Frequency 274 cm^{-1} plotted on the optimized S_1 geometry of R-MeCzCN. (**b**) Frequency 471 cm^{-1} plotted on the optimized S_1 geometry of R-MeCzCN.

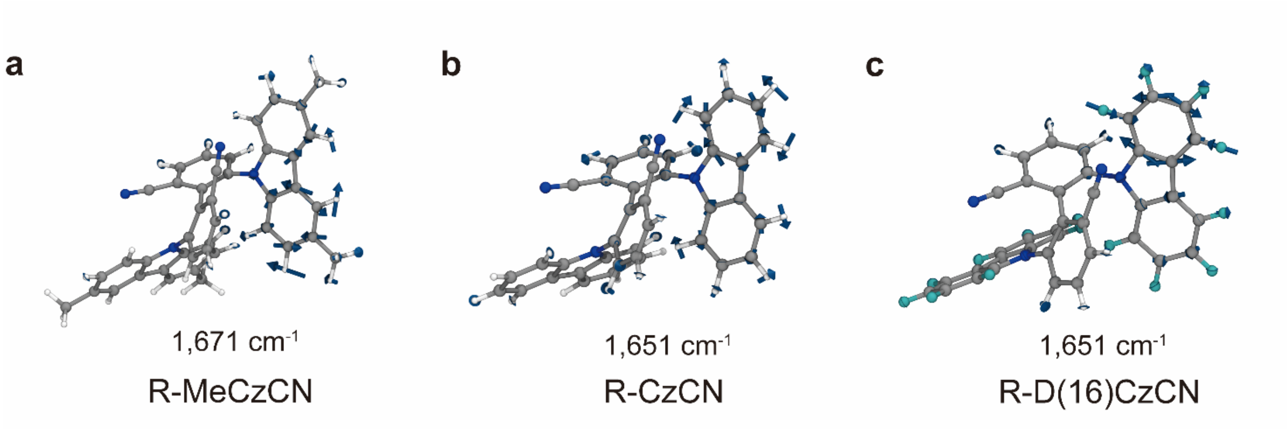

Supplementary Fig. 14. Vector displacement diagram of the high-frequency modes. (**a**) Frequency 1,671 cm^{-1} plotted on the optimized S_1 geometry of R-MeCzCN. (**b**) Frequency 1,651 cm^{-1} plotted on the optimized S_1 geometry of R-CzCN. (**c**) Frequency 1,651 cm^{-1} plotted on the optimized S_1 geometry of R-D(16)CzCN.

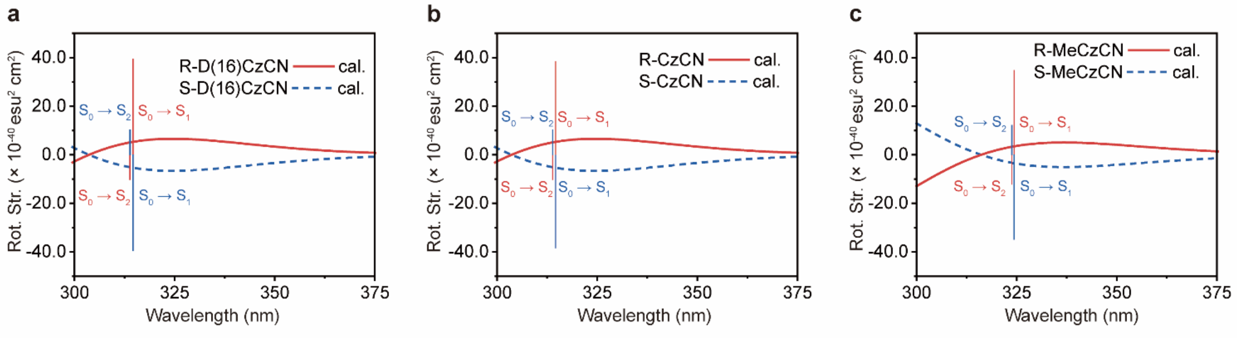

Supplementary Fig. 15. Simulated CD spectra at the CAM-B3LYP/def2-SVP level based on optimized S_0 geometries. (**a**) R/S-D(16)CzCN. (**b**) R/S-CzCN. (**c**) R/S-MeCzCN.

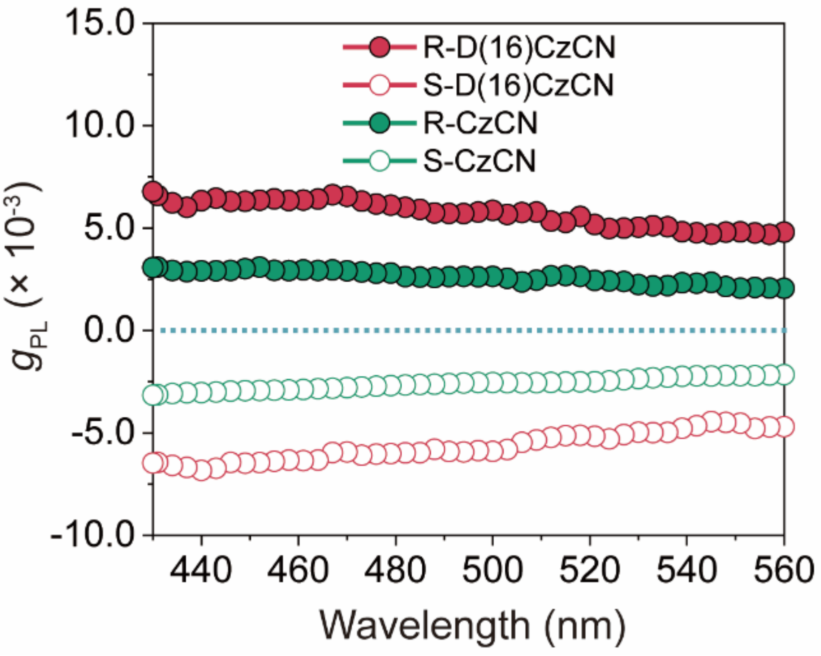

Supplementary Fig. 16. ***g_{PL}*** **values versus wavelength curves of deuterated R/S-D(16)CzCN and non-deuterated R/S-CzCN as chiral emitters.**

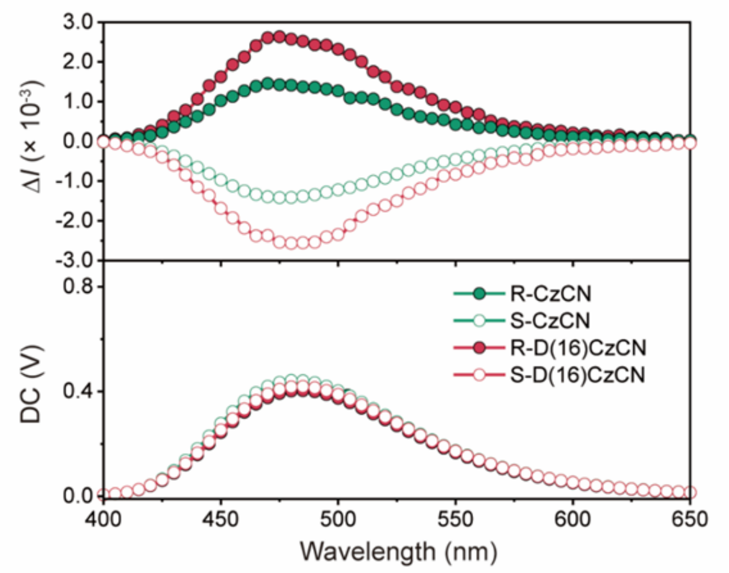

Supplementary Fig. 17. Δ*I* and DC values versus wavelength curves of R/S-CzCN and R/S-D(16)CzCN.

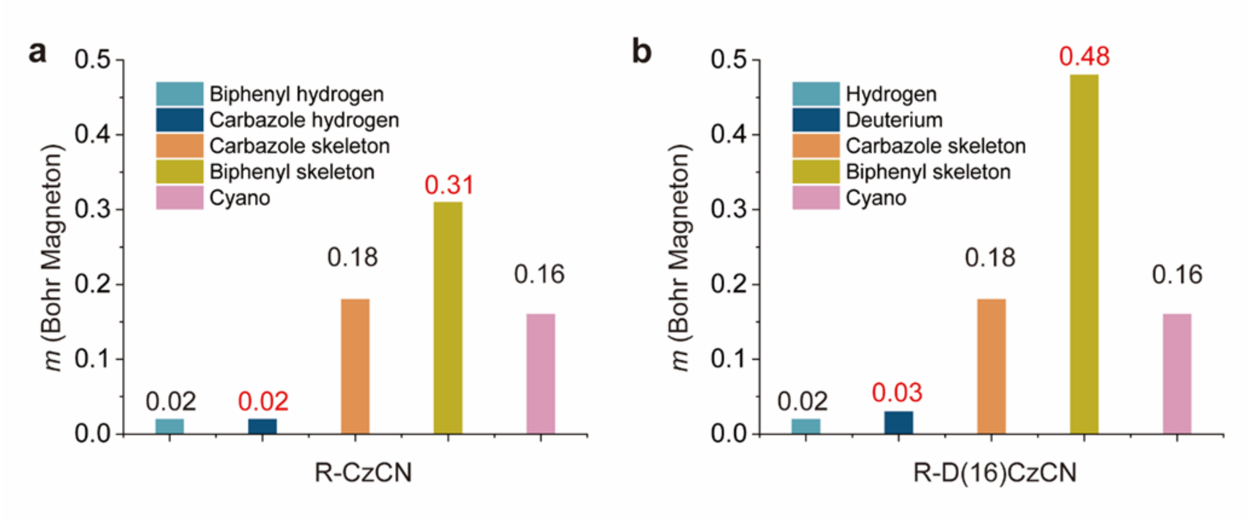

Supplementary Fig. 18. Atomic and fragment contributions to m. (**a**) R-CzCN. (**b**) R-D(16)CzCN.

Note: The scalar sum of fragment |**m**| values is larger than the whole-molecule **m** obtained by vector summation, because the fragment **m** vectors are not collinear and partially cancel.

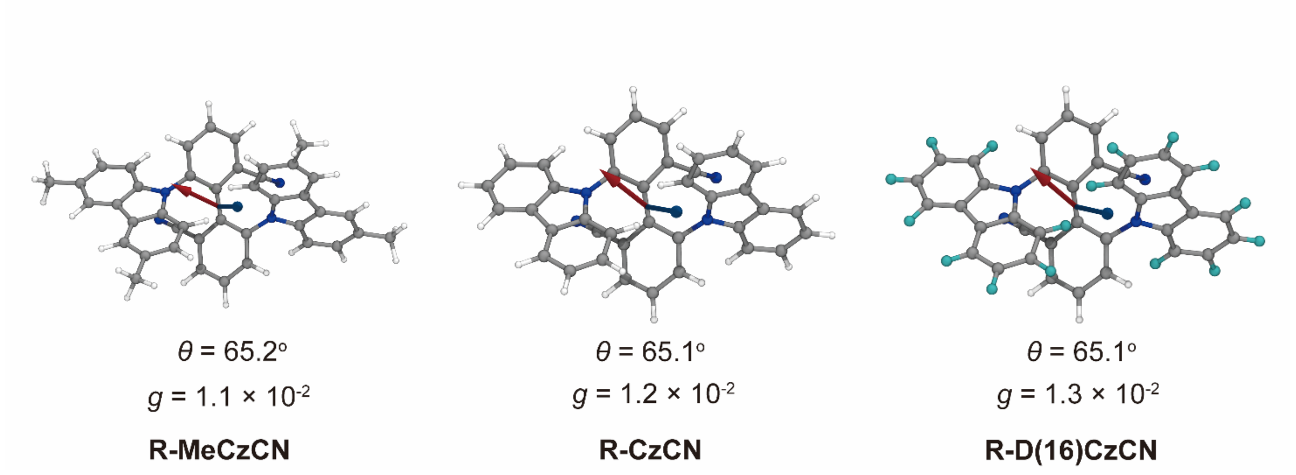

Supplementary Fig. 19. Calculated orientation distributions and angles (*θ*, in units of deg) of the μ (red) and m (blue) and theoretically calculated *g*-factors of stationary-point structures: R-MeCzCN; R-CzCN; R-D(16)CzCN.

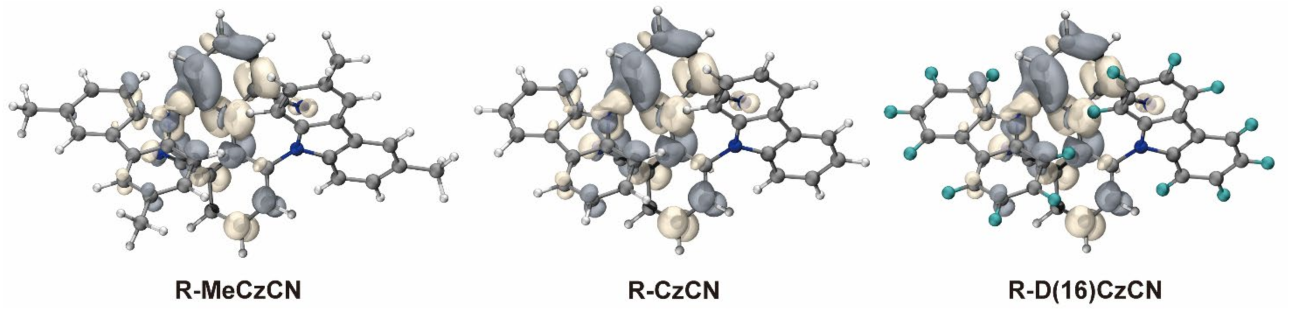

Supplementary Fig. 20. $S_1 \rightarrow S_0$ transition density distributions of R-MeCzCN, R-CzCN and R-D(16)CzCN calculated from stationary-point TDDFT calculations.

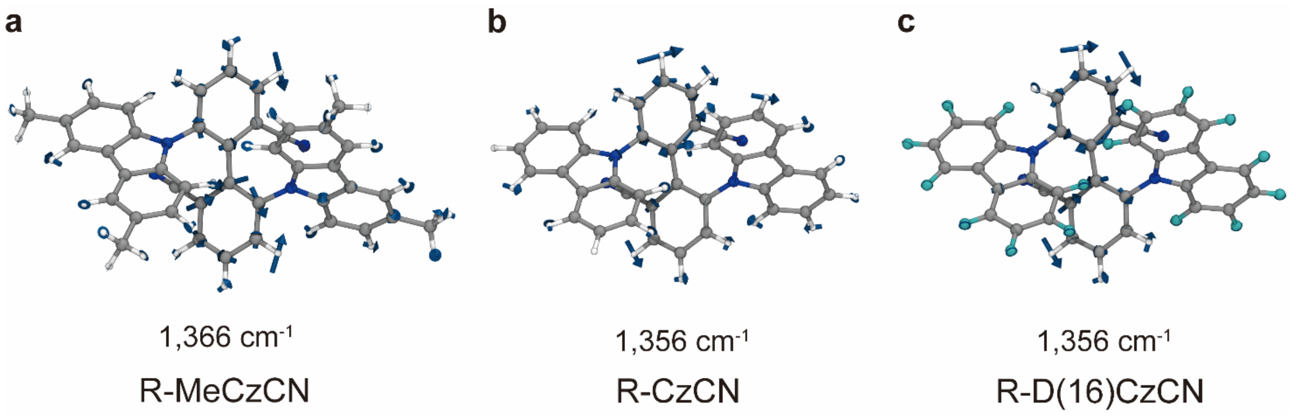

Supplementary Fig. 21. Vector displacement diagram of the normal mode with a frequency of about 1,360 cm^{-1}. (**a**) Frequency 1,366 cm^{-1} plotted on the optimized S_1 geometry of R-MeCzCN. (**b**) Frequency 1,356 cm^{-1} plotted on the optimized S_1 geometry of R-CzCN. (**c**) Frequency 1,356 cm^{-1} plotted on the optimized S_1 geometry of R-D(16)CzCN.

ω_k	q(k)	$\{\Delta\rho\}_k$	g_k
489 cm^{-1}	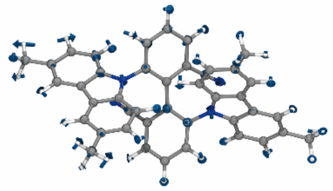 R-MeCzCN	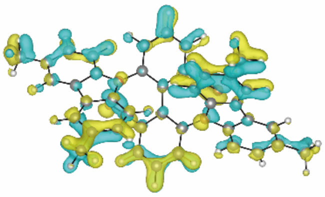 R-MeCzCN	1.9×10^{-3}
487 cm^{-1}	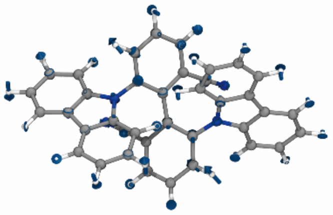 R-CzCN	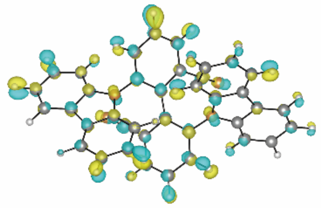 R-CzCN	6.2×10^{-3}
475 cm^{-1}	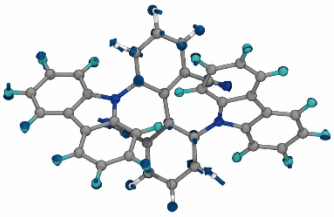 R-D(16)CzCN	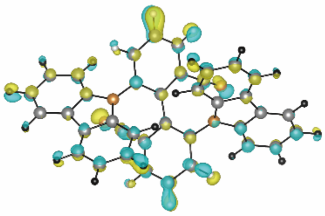 R-D(16)CzCN	7.5×10^{-3}
807 cm^{-1}	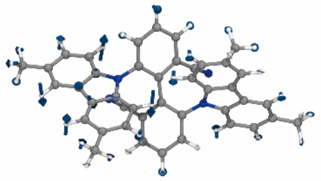 R-MeCzCN	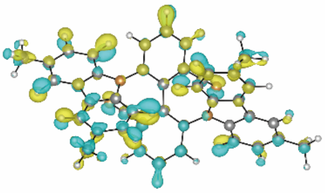 R-MeCzCN	4.1×10^{-3}
800 cm^{-1}	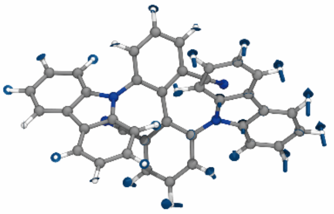 R-CzCN	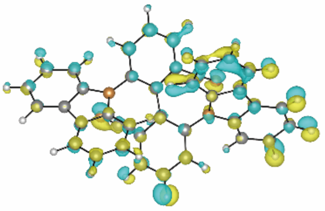 R-CzCN	5.1×10^{-3}
779 cm^{-1}	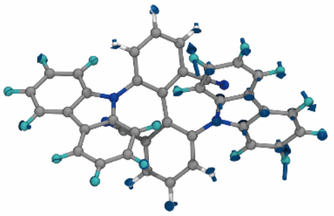 R-D(16)CzCN	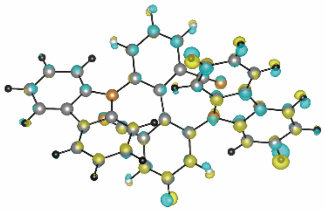 R-D(16)CzCN	7.4×10^{-3}

Supplementary Fig. 22. Perturbational effect on the exciton wavefunction (transition density) of the S_1 state along the coordinate of representative vibrational modes. In the table, ω_k corresponds to the frequency of the kth vibrational mode. q(k) corresponds to the vector displacement diagram of the kth mode. $\{\Delta\rho\}_k$ is the differential exciton wavefunction (transition density) upon displacement along the kth mode for S_1 exciton. g_k corresponds to the Boltzmann-weighted g-factors of the kth vibrational mode.

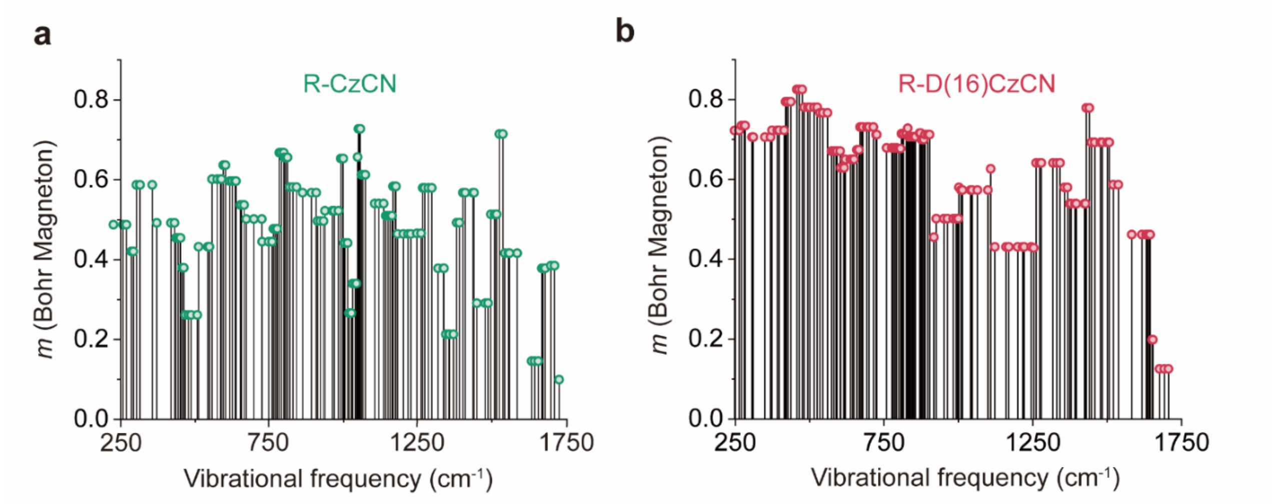

Supplementary Fig. 23. Theoretically calculated m versus vibrational frequency. (**a**) R-CzCN. (**b**) R-D(16)CzCN.

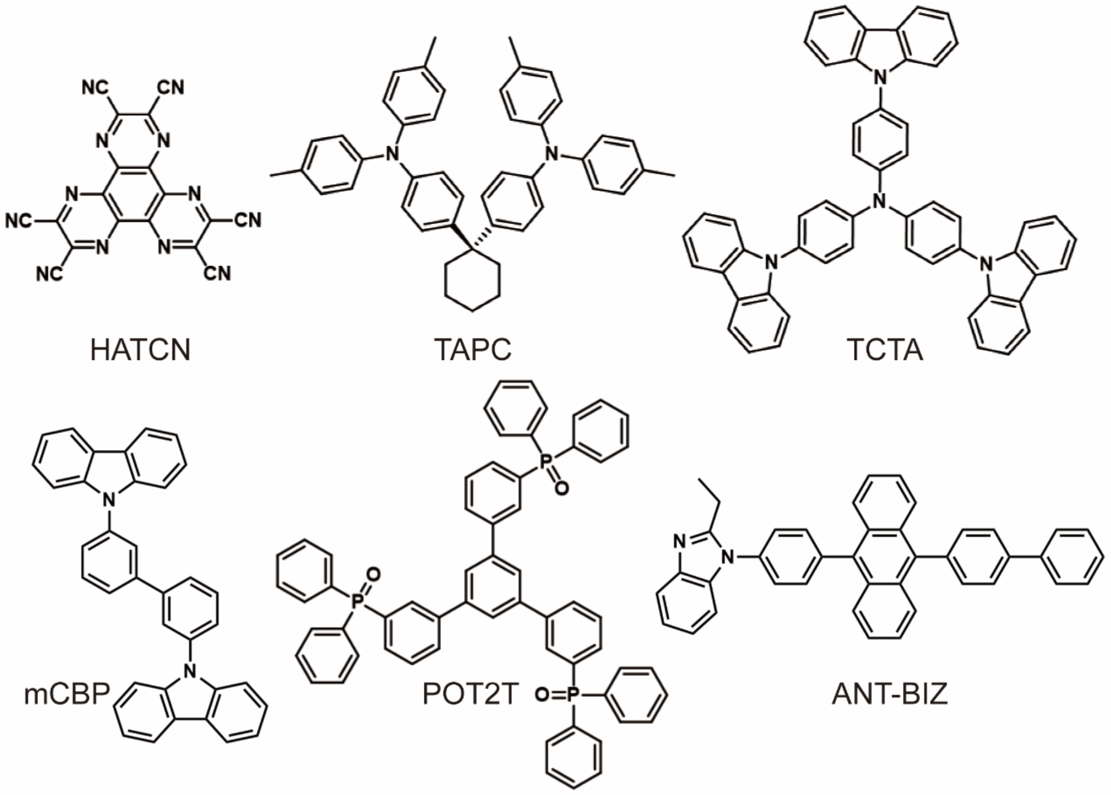

Supplementary Fig. 24. Chemical structures of the comprising materials in CP-OLEDs.

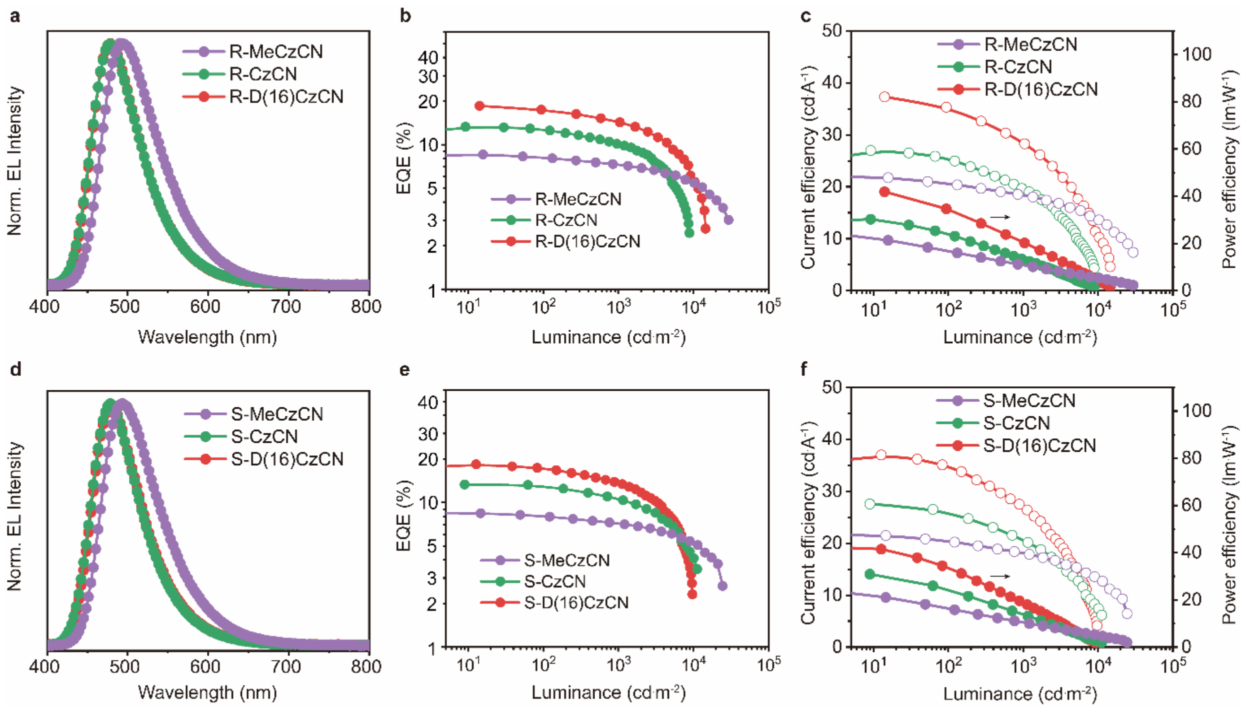

Supplementary Fig. 25. Conventional CP-OLEDs based on R/S-chiral emitters. (**a**) Normalized electroluminescence spectra of CP-OLEDs based on R-MeCzCN, R-CzCN and R-D(16)CzCN. (**b**) EQE versus luminance characteristics of CP-OLEDs based on R-MeCzCN, R-CzCN and R-D(16)CzCN. (**c**) Current and power efficiency versus luminance characteristics of CP-OLEDs based on R-MeCzCN, R-CzCN and R-D(16)CzCN. (**d**) Normalized electroluminescence spectra of CP-OLEDs based on S-MeCzCN, S-CzCN and S-D(16)CzCN. (**e**) EQE versus luminance characteristics of CP-OLEDs based on S-MeCzCN, S-CzCN and S-D(16)CzCN. (**f**) Current and power efficiency versus luminance characteristics of CP-OLEDs based on S-MeCzCN, S-CzCN and S-D(16)CzCN.

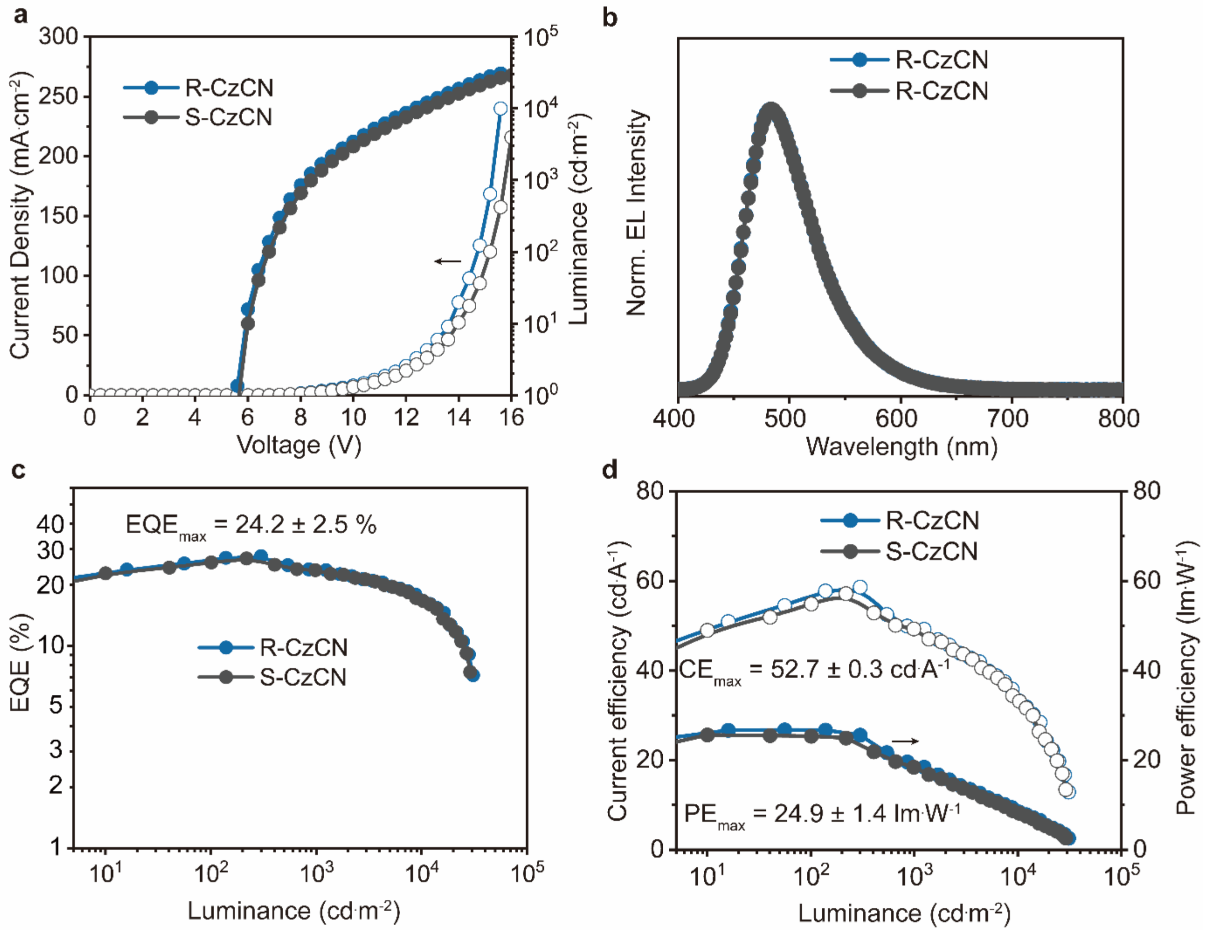

Supplementary Fig. 26. Tandem CP-OLEDs based on R/S-CzCN. (**a**) Current density and luminance versus driving voltage characteristics. (**b**) Normalized electroluminescence spectra. (**c**) EQE versus luminance characteristics. (**d**) Current efficiency and power efficiency versus luminance characteristics.

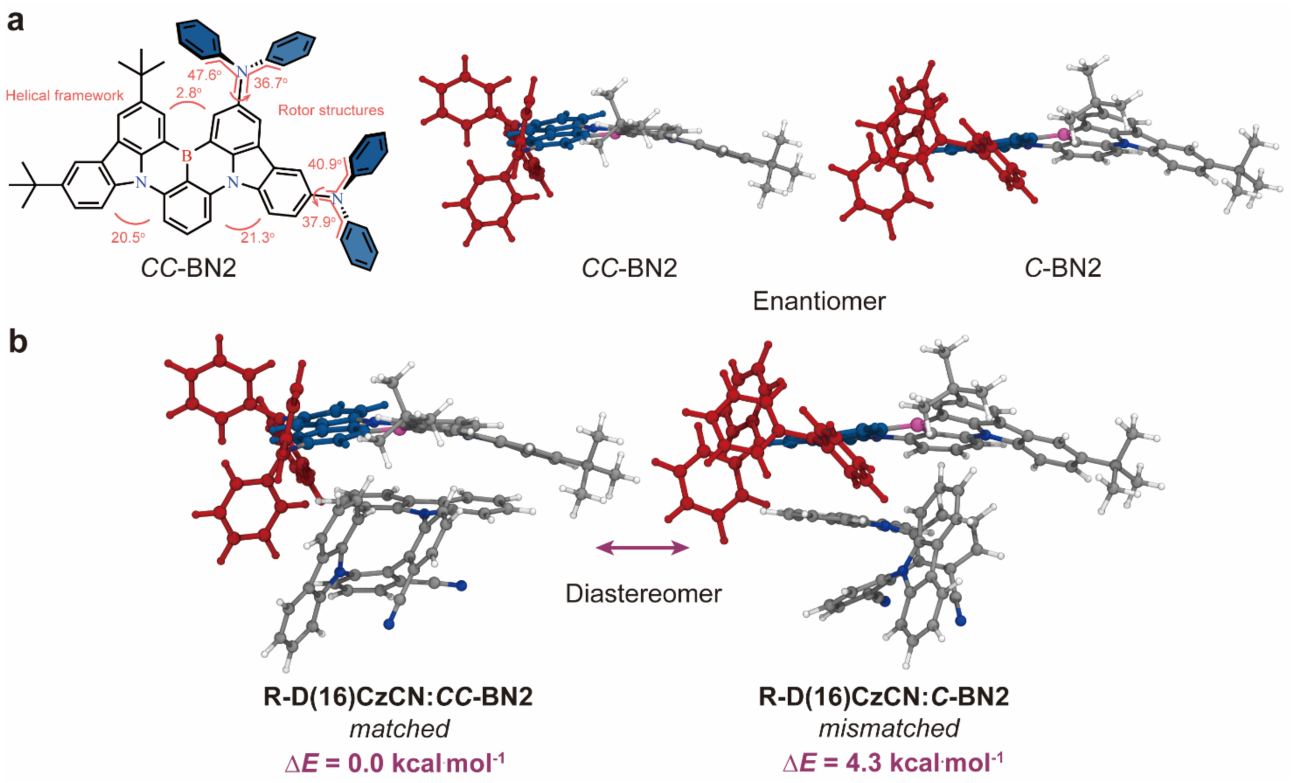

Supplementary Fig. 27. BN2 enantiomers and diastereomeric interactions with the chiral host. (**a**) The chemical structures of BN2 and the optimized geometries of BN2 enantiomers, *CC*-BN2 and *C*-BN2. (**b**) Calculated diastereomeric interactions between R-D(16)CzCN and the BN2 enantiomers (*CC*-BN2 and *C*-BN2). Geometries and relative energies were computed at the B3LYP-D3(BJ)/def2-SVP level of theory.

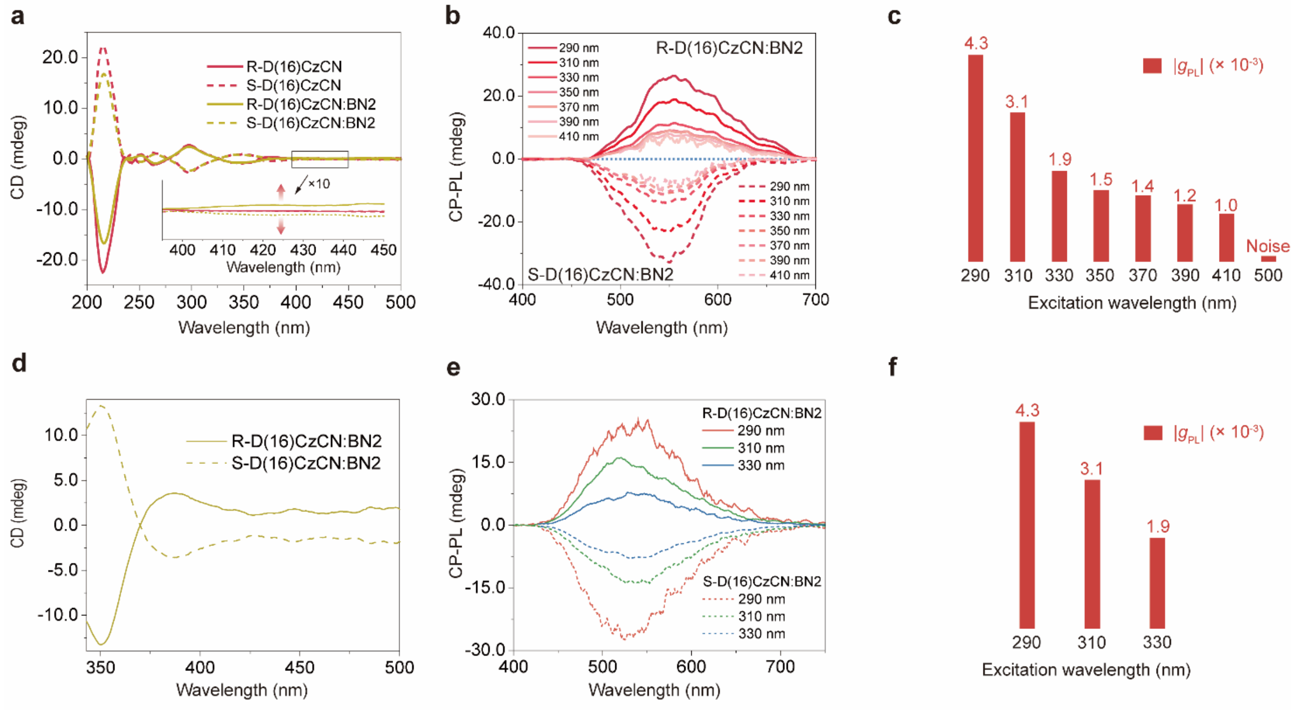

Supplementary Fig. 28. Chiroptical properties of R/S-D(16)CzCN:BN2. (**a**) Experimental CD spectra of R/S-D(16)CzCN neat films and R/S-D(16)CzCN:BN2 films. (**b**) CP-PL spectra of R/S-D(16)CzCN:BN2 films under excitation at 290, 310, 330, 350, 370, 390 and 410 nm. (**c**) Column diagrams of |g_{PL}| values of R/S-D(16)CzCN:BN2 films excited at 290, 310, 330, 350, 370, 390, 410 and 500 nm. (**d**) Experimental CD spectra of R/S-D(16)CzCN:BN2 in THF/water mixtures (V_{THF}:V_{water} = 10:90) at the concentration of 1 mM. (**e**) CP-PL spectra of R/S-D(16)CzCN:BN2 in THF/water mixtures (V_{THF}:V_{water} = 10:90) at the concentration of 1 mM, under excitation at 290, 310, and 330 nm. (**f**) Column diagrams of |g_{PL}| values of R/S-D(16)CzCN:BN2 in THF/water mixtures (V_{THF}:V_{water} = 10:90) at the concentration of 1 mM, excited at 290, 310, and 330 nm.

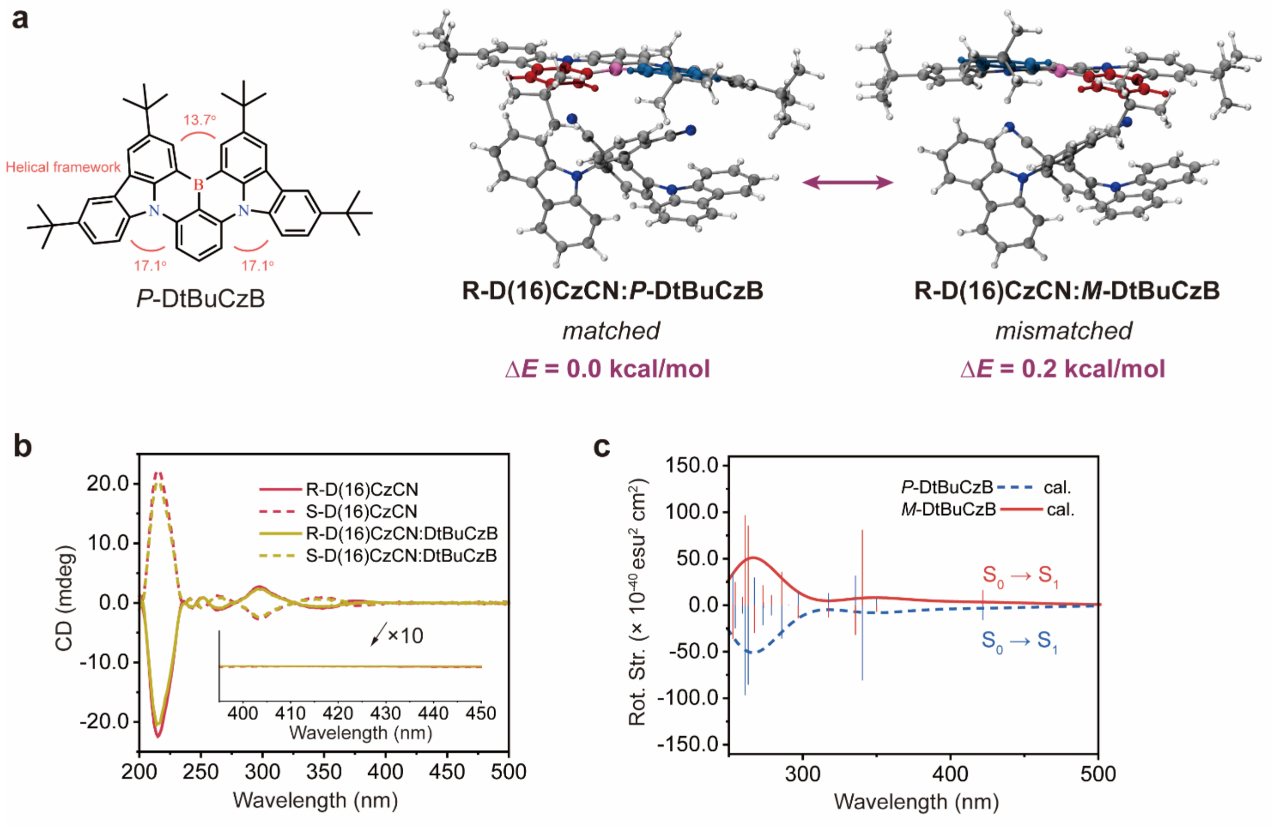

Supplementary Fig. 29. Diastereomeric interactions and chiroptical properties of R/S-D(16)CzCN:DtBuCzB. (**a**) Calculated diastereomeric interactions between R-D(16)CzCN and the DtBuCzB enantiomers (*P*-DtBuCzB and *M*-DtBuCzB). Geometries and relative energies were computed at the B3LYP-D3(BJ)/def2-SVP level of theory. (**b**) Experimental CD spectra of R/S-D(16)CzCN neat films and R/S-D(16)CzCN:DtBuCzB films. (**c**) Calculated CD spectra of DtBuCzB enantiomers.

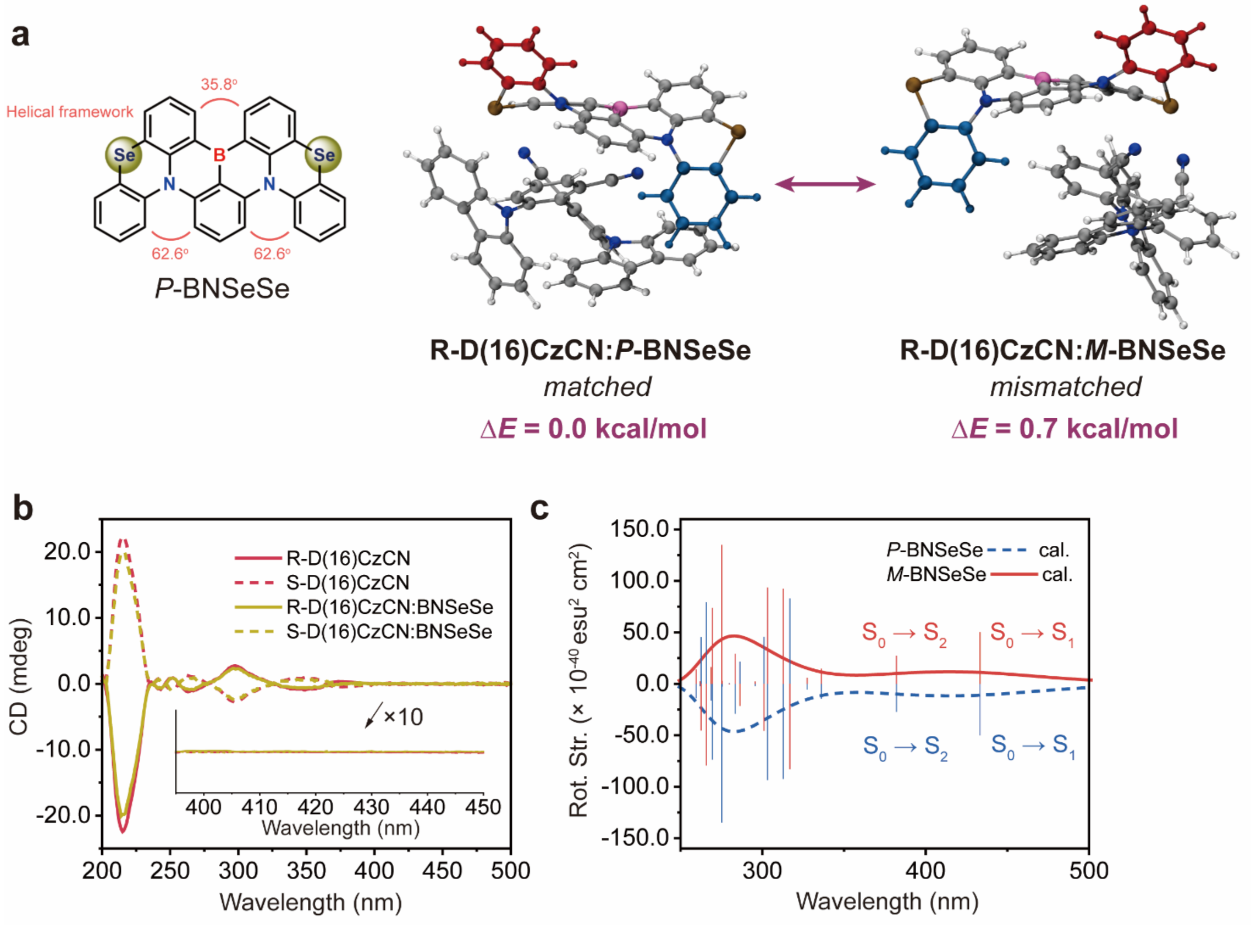

Supplementary Fig. 30. Diastereomeric interactions and chiroptical properties of R/S-D(16)CzCN:BNSeSe. (**a**) Calculated diastereomeric interactions between R-D(16)CzCN and the BNSeSe enantiomers (*P*-BNSeSe and *M*-BNSeSe). Geometries and relative energies were computed at the B3LYP-D3(BJ)/def2-SVP level of theory.. (**b**) Experimental CD spectra of R/S-D(16)CzCN neat films and R/S-D(16)CzCN:BNSeSe films. (**c**) Calculated CD spectra of BNSeSe enantiomers.

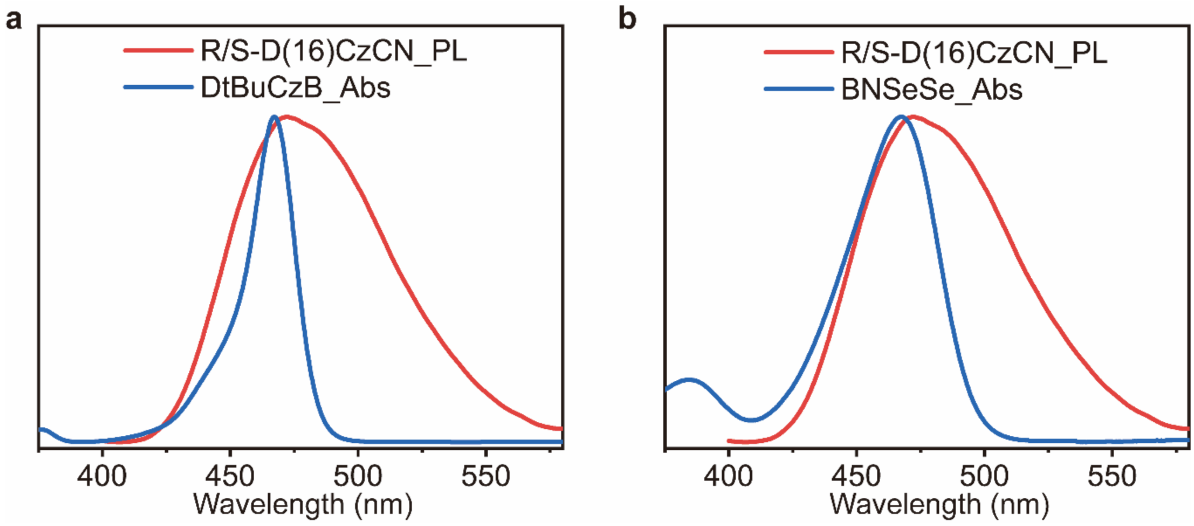

Supplementary Fig. 31. Förster resonance energy transfer. Overlap between the normalized PL spectra of R/S-D(16)CzCN neat film and extinction spectrum of achiral MR-TADF emitter (**a**) DtBuCzB and (**b**) BNSeSe in solution with a concentration of 10^{-5} mol·L^{-1}.

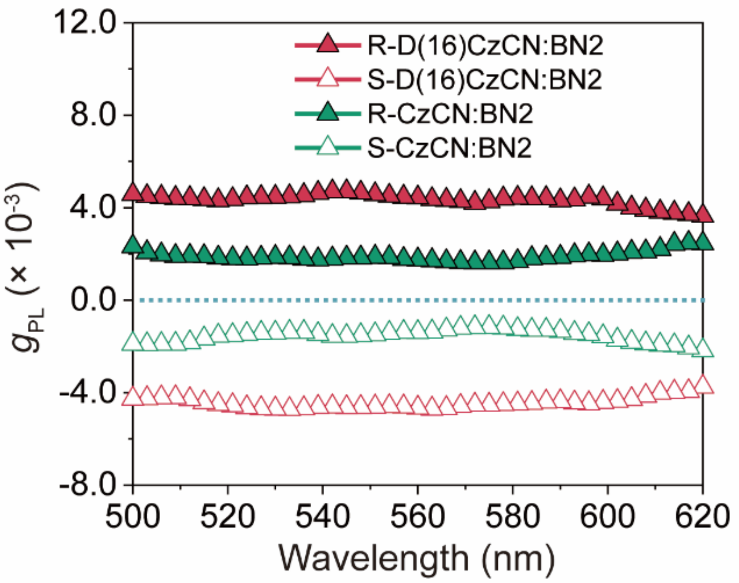

Supplementary Fig. 32. g_{PL} **values versus wavelength curves of deuterated R/S-D(16)CzCN and non-deuterated R/S-CzCN as chiral hosts.**

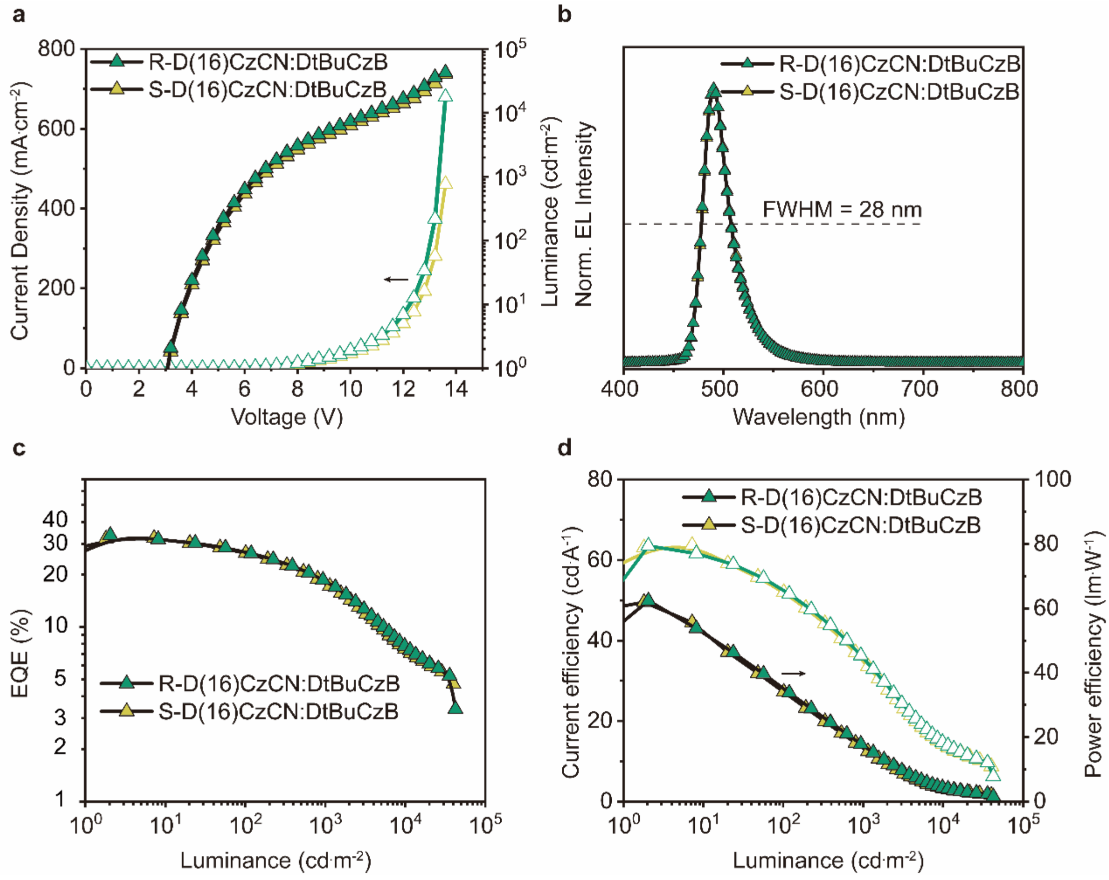

Supplementary Fig. 33. CP-OLEDs based on R/S-D(16)CzCN:DtBuCzB. (**a**) Current density and luminance versus driving voltage characteristics. (**b**) Normalized electroluminescence spectra. (**c**) EQE versus luminance characteristics (average, 32.3 ± 0.9%; highest, 33.6%). (**d**) Current and power efficiency versus luminance characteristics.

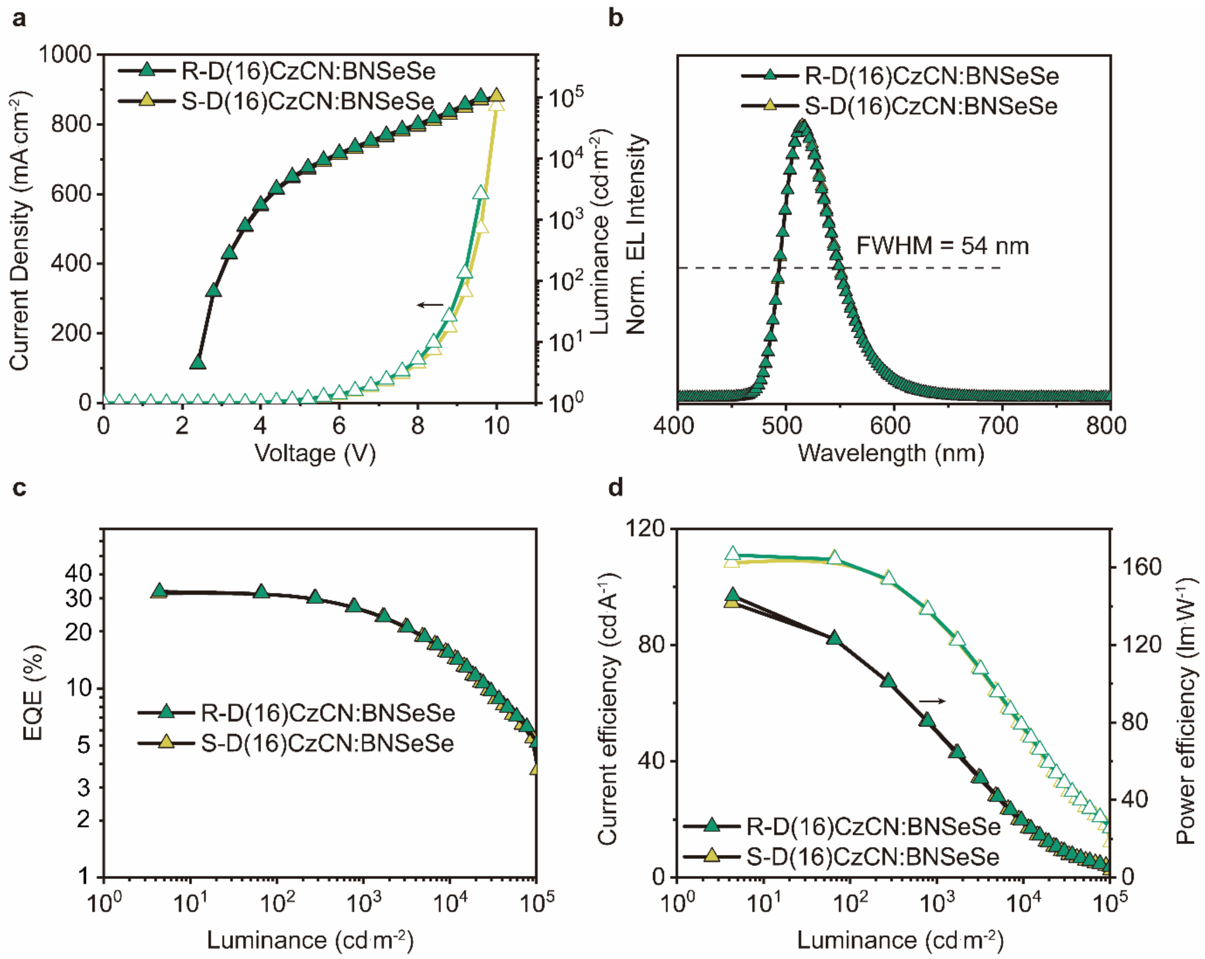

Supplementary Fig. 34. CP-OLED s based on R/S-D(16)CzCN:BNSeSe. (**a**) Current density and luminance versus driving voltage characteristics. (**b**) Normalized electroluminescence spectra. (c) EQE versus luminance characteristics (average, 32.1 ± 0.2%; highest, 32.4%). (d) Current and power efficiency versus luminance characteristics.

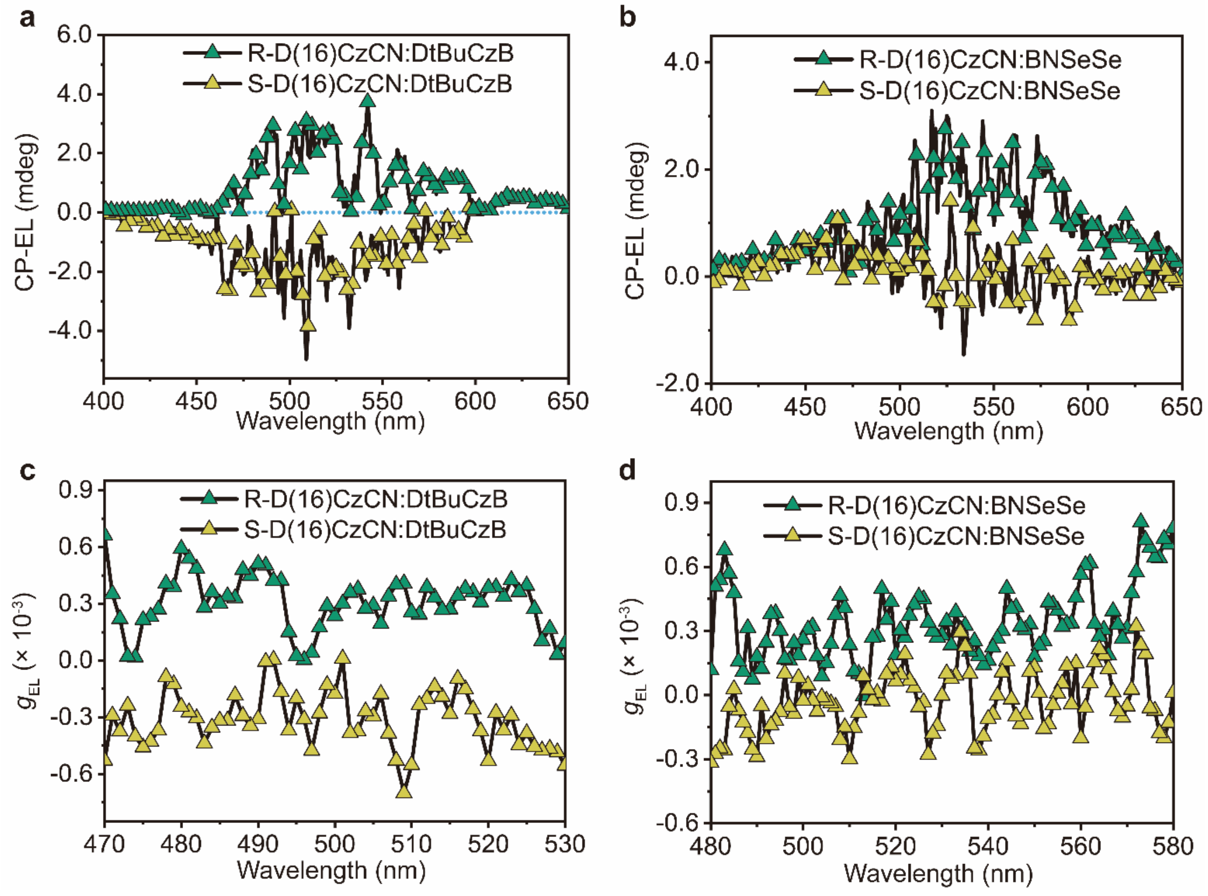

Supplementary Fig. 35. Circular polarization of CP-OLEDs based on R/S-D(16)CzCN:DtBuCzB and R/S-D(16)CzCN:BNSeSe. CP-EL spectra of CP-OLEDs based on (**a**) R/S-CzCN:DtBuCzB and (**b**) R/S-MeCzCN:BNSeSe. g_{EL} values versus wavelength curves of CP-OLEDs based on (**c**) R/S-CzCN:DtBuCzB and (**d**) R/S-MeCzCN:BNSeSe.

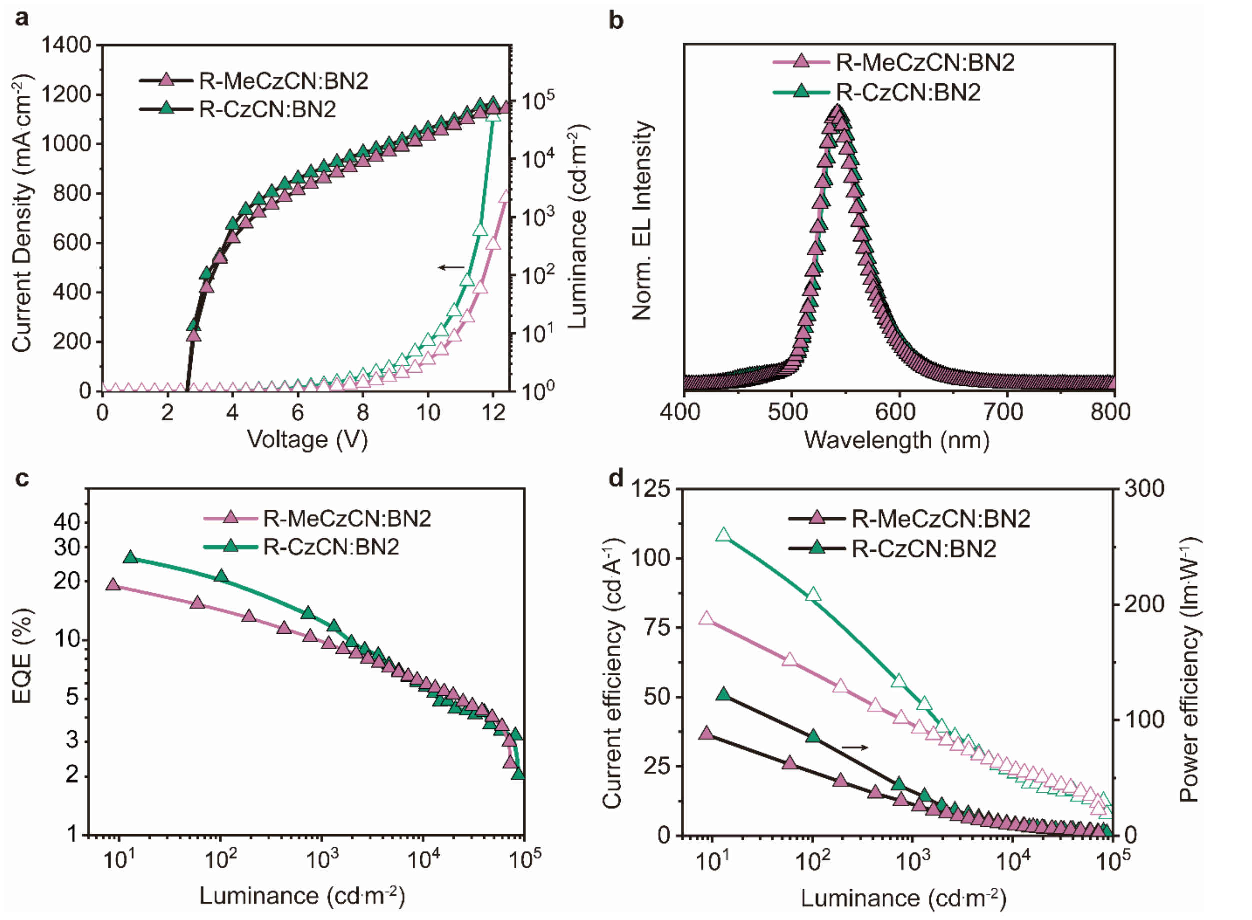

Supplementary Fig. 36. CP-OLEDs based on R-MeCzCN:BN2 and R-CzCN:BN2. (**a**) Current density and luminance versus driving voltage characteristics. (**b**) Normalized electroluminescence spectra. (**c**) EQE versus luminance characteristics. (**d**) Current and power efficiency versus luminance characteristics.

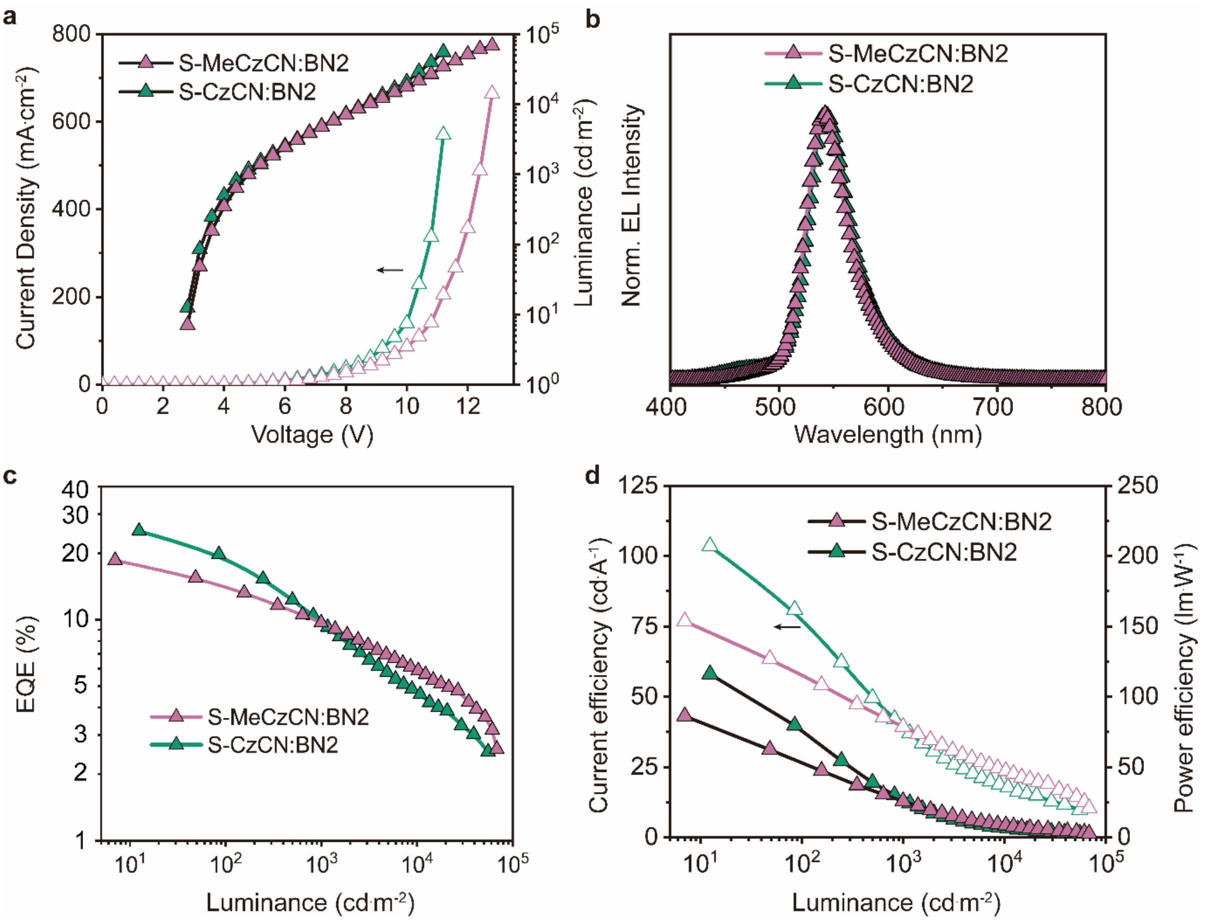

Supplementary Fig. 37. CP-OLEDs based on S-MeCzCN:BN2 and S-CzCN:BN2. (**a**) Current density and luminance versus driving voltage characteristics. (**b**) Normalized electroluminescence spectra. (**c**) EQE versus luminance characteristics. (**d**) Current and power efficiency versus luminance characteristics.

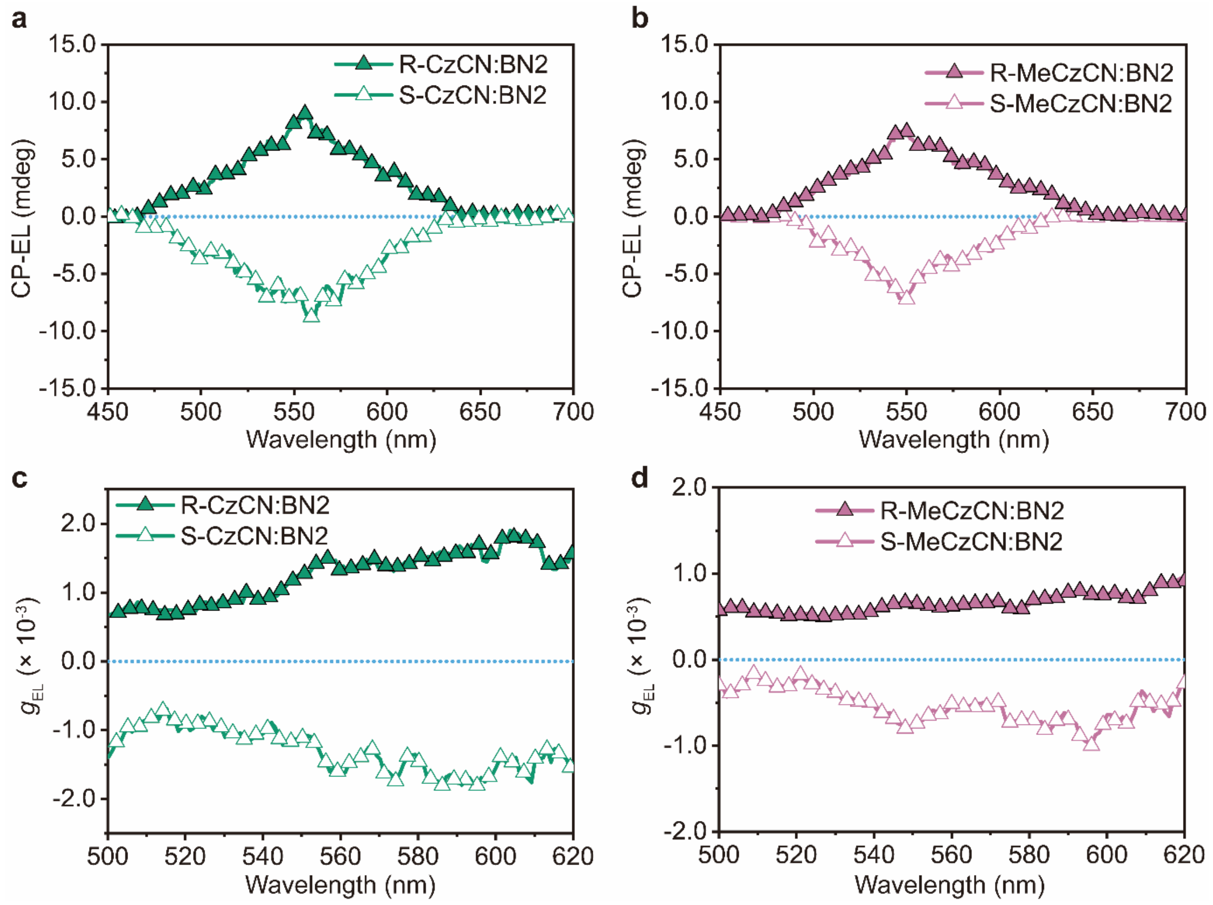

Supplementary Fig. 38. Circular polarization of CP-OLEDs based on R/S-CzCN:BN2 and R/S-MeCzCN:BN2. CP-EL spectra of CP-OLEDs based on (**a**) R/S-CzCN:BN2 and (**b**) R/S-MeCzCN:BN2. g_{EL} values versus wavelength curves of CP-OLEDs based on (**c**) R/S-CzCN:BN2 and (**d**) R/S-MeCzCN:BN2.

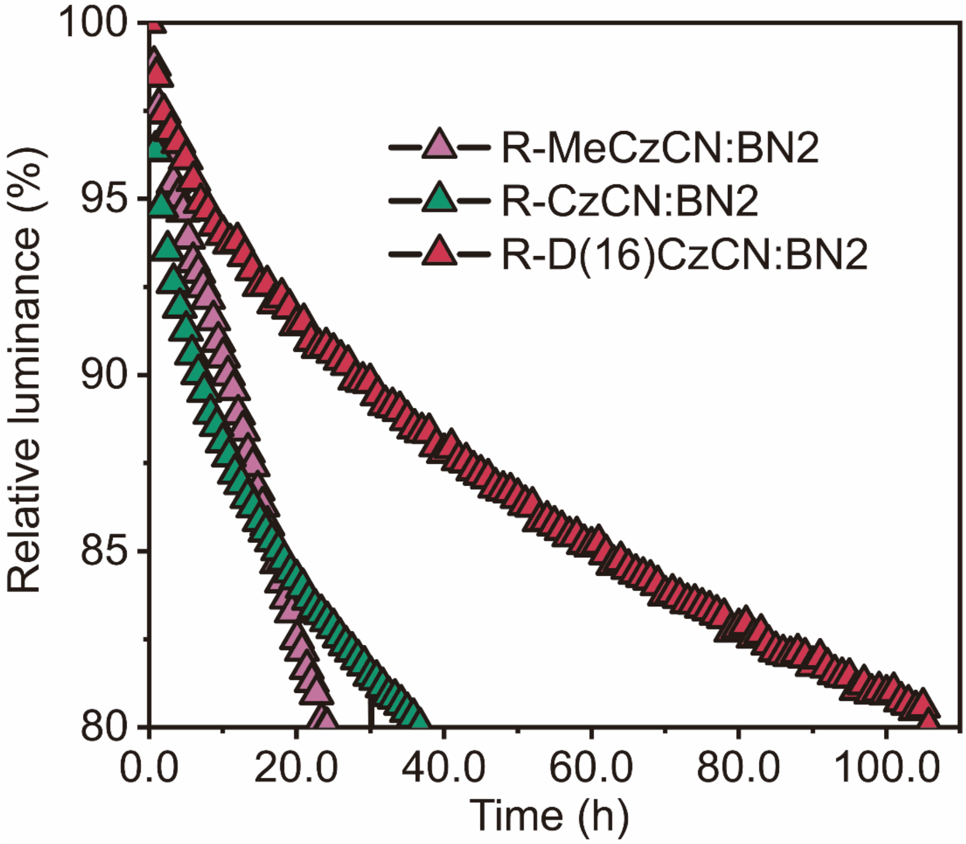

Supplementary Fig. 39. Operational lifetimes of the conventional CP-OLEDs based on R-D(16)CzCN:BN2, R-CzCN:BN2 and R-MeCzCN:BN2, measured at an initial luminance of 1,000 cd·m^{-2}.

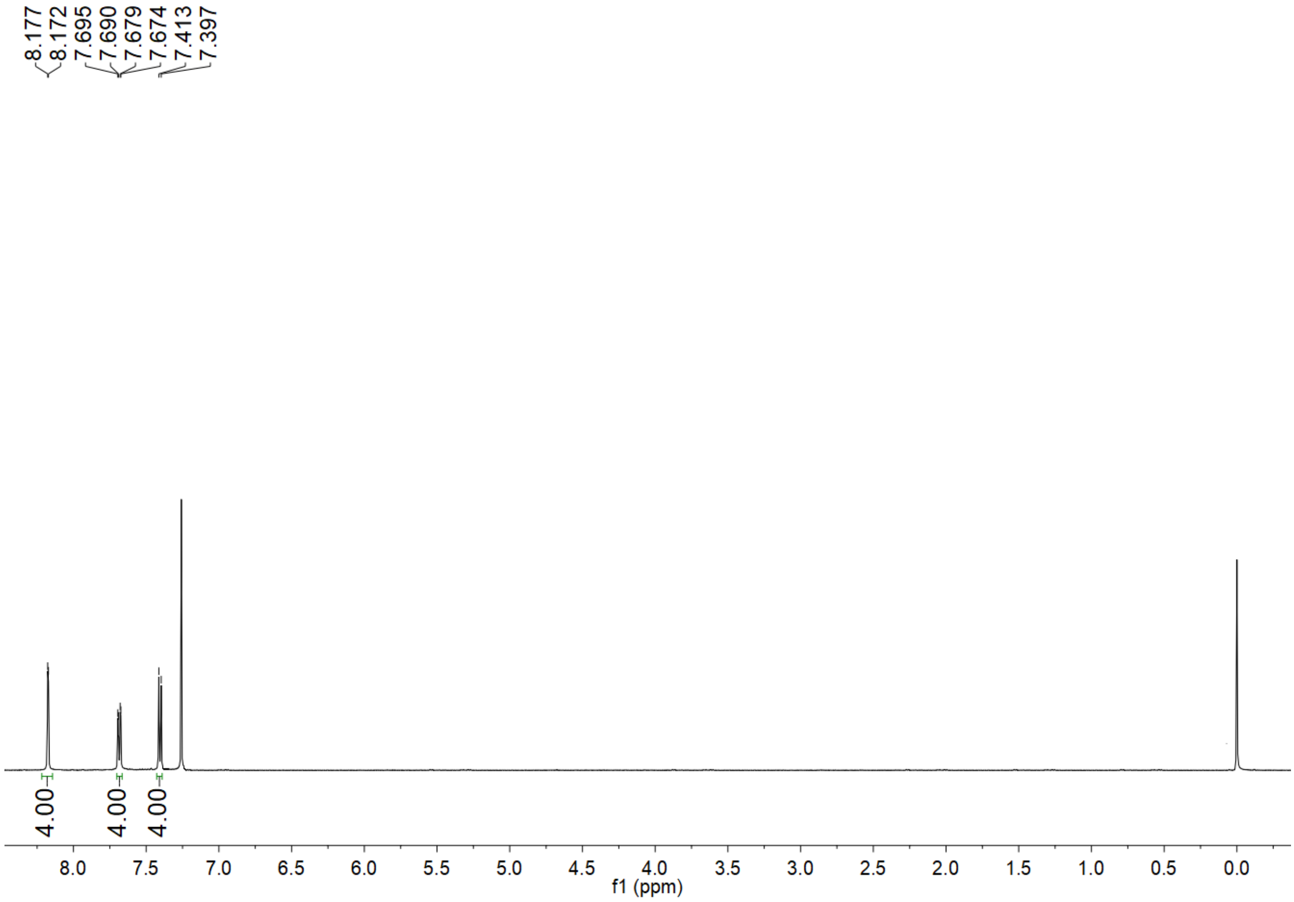

Supplementary Fig. 40. ^{1}H NMR spectrum of (P,P)/(M,M)-D(32)CzTBCO in $CDCl_3$ at room temperature.

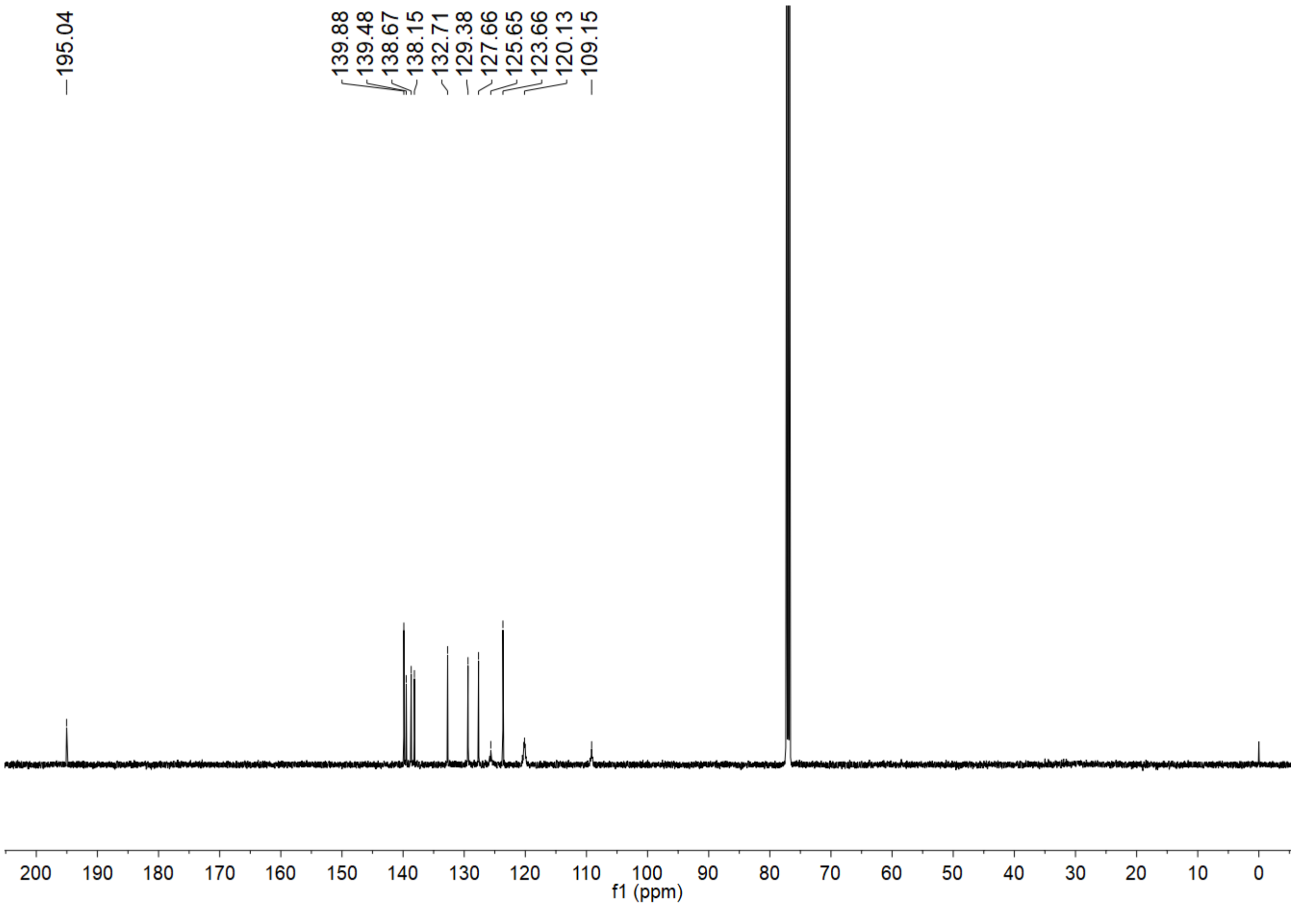

Supplementary Fig. 41. ^{13}**C NMR spectrum of (P,P)/(M,M)-D(32)CzTBCO in** $CDCl_3$ **at room temperature.**

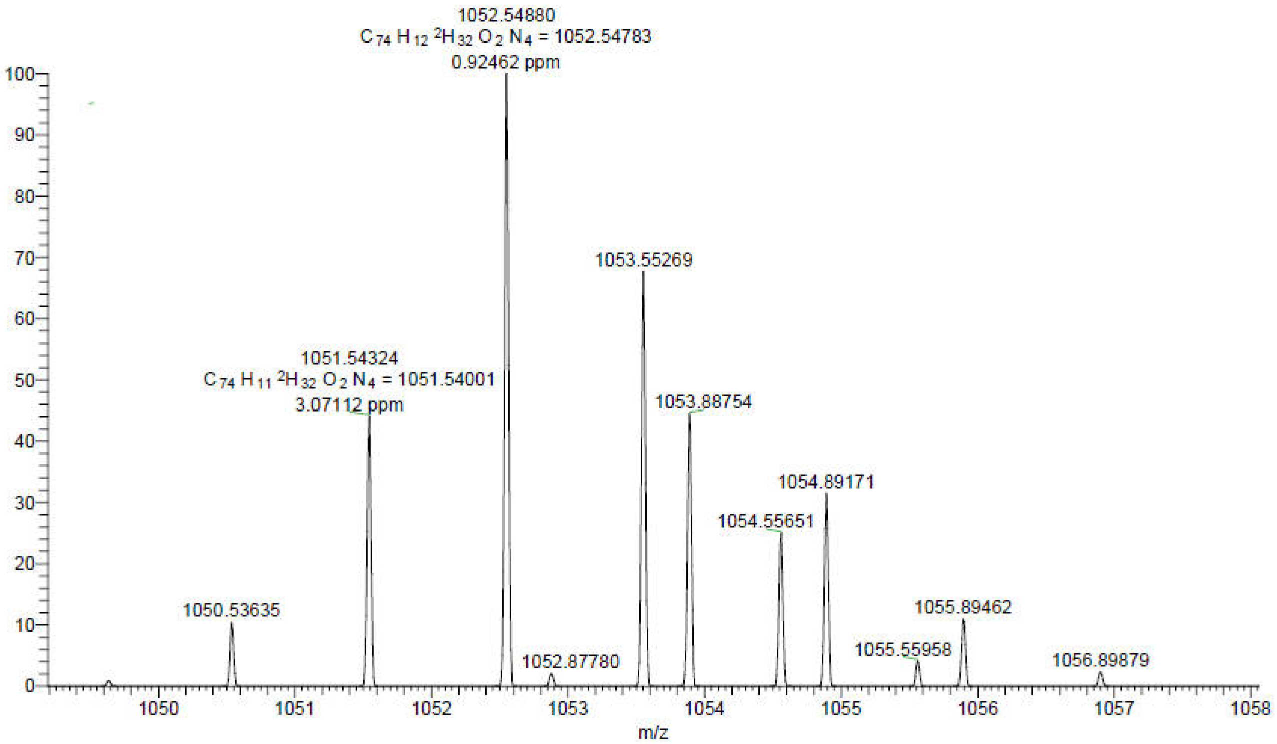

Supplementary Fig. 42. HRMS spectrum of (P,P)/(M,M)-D(32)CzTBCO.

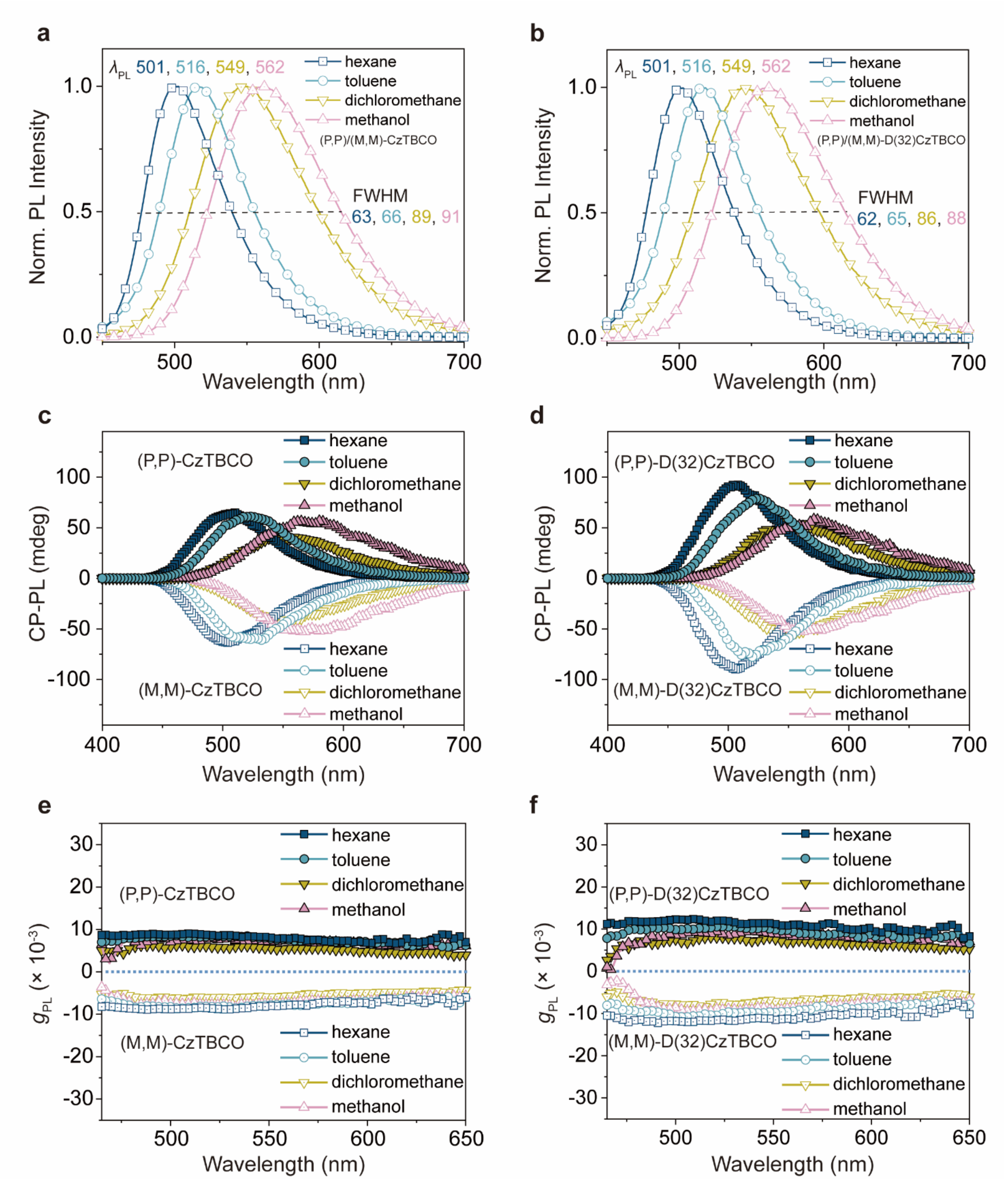

Supplementary Fig. 43. Photophysical and chiroptical properties of (P,P)/(M,M)-CzTBCO and (P,P)/(M,M)-D(32)CzTBCO in different solvents (1 × 10^{-5} M) at 300 K. (**a**) PL spectra of (P,P)/(M,M)-CzTBCO in different solvents (1 × 10^{-5} M) at 300 K. (**b**) PL spectra of (P,P)/(M,M)-D(32)CzTBCO in different solvents (1 × 10^{-5} M) at 300 K. (**c**) CP-PL spectra of (P,P)/(M,M)-CzTBCO in different solvents (1 × 10^{-5} M) at 300 K. (**d**) CP-PL spectra of (P,P)/(M,M)-D(32)CzTBCO in different solvents (1 × 10^{-5} M) at 300 K. (**e**) g_{PL} values versus wavelength curves of (P,P)/(M,M)-CzTBCO in different solvents (1 × 10^{-5} M) at 300 K. (**f**) g_{PL} values versus wavelength curves of (P,P)/(M,M)-D(32)CzTBCO in different solvents (1 × 10^{-5} M) at 300 K.

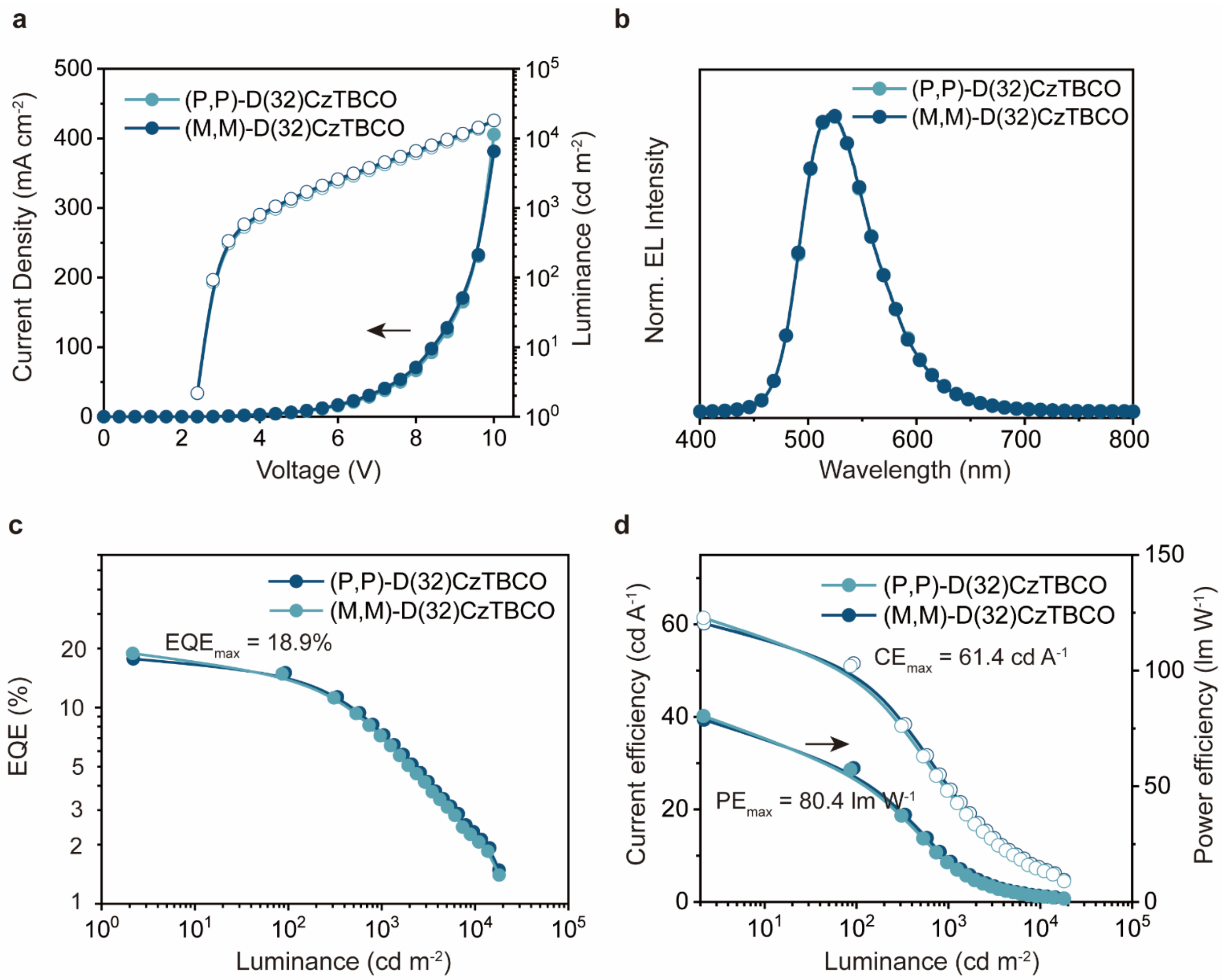

Supplementary Fig. 44. CP-OLEDs based on (P,P)/(M,M)-D(32)CzTBCO. (**a**) Current density and luminance versus driving voltage characteristics. (**b**) Normalized electroluminescence spectra. (**c**) EQE versus luminance characteristics. (**d**) Current and power efficiency versus luminance characteristics.

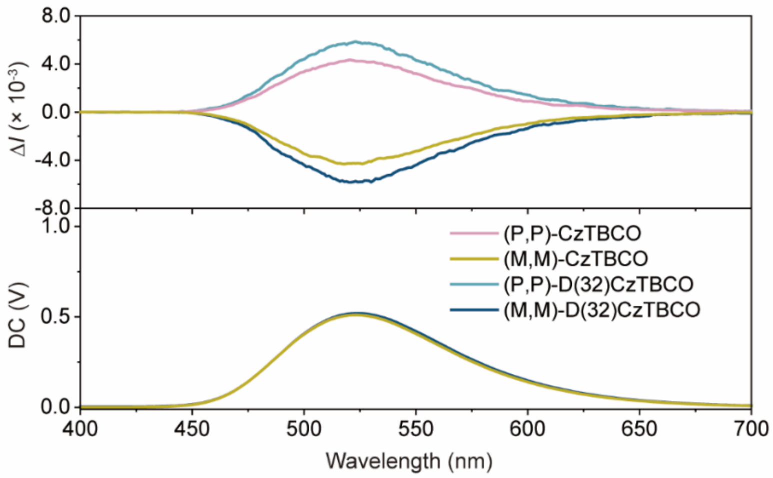

Supplementary Fig. 45. Δ*I* and DC values versus wavelength curves of (P,P)/(M,M)-CzTBCO and (P,P)/(M,M)-D(32)CzTBCO.

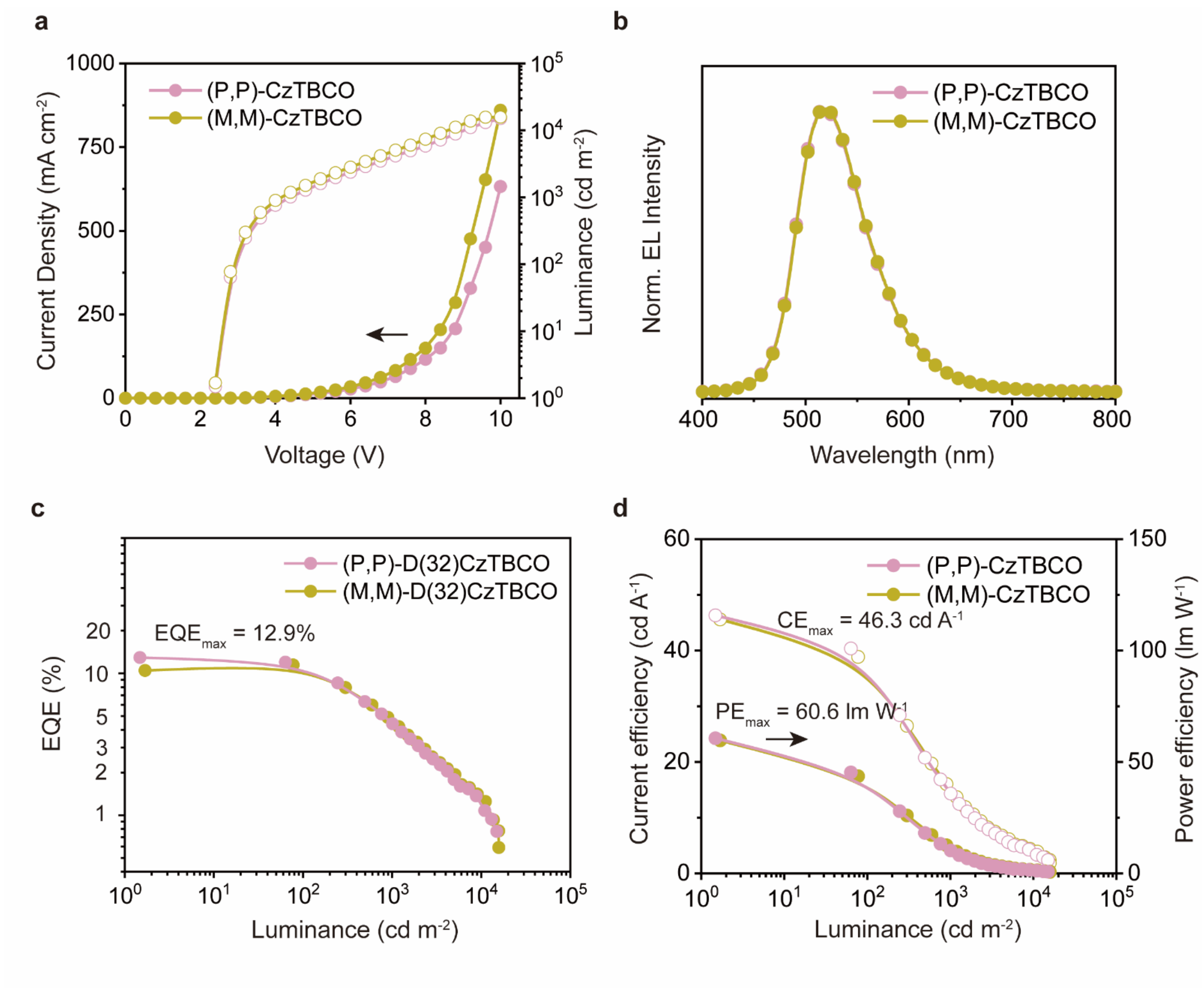

Supplementary Fig. 46. CP-OLEDs based on (P,P)/(M,M)-CzTBCO. (**a**) Current density and luminance versus driving voltage characteristics. (**b**) Normalized electroluminescence spectra. (**c**) EQE versus luminance characteristics. (**d**) Current and power efficiency versus luminance characteristics.

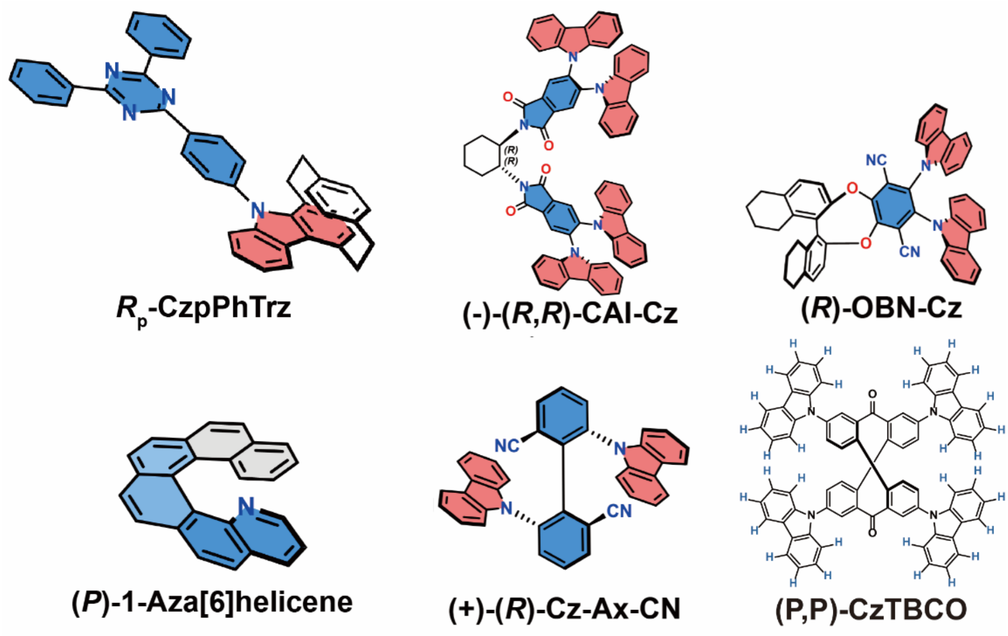

Supplementary Fig. 47. Chemical structures of representative organic chiral emitters.

a

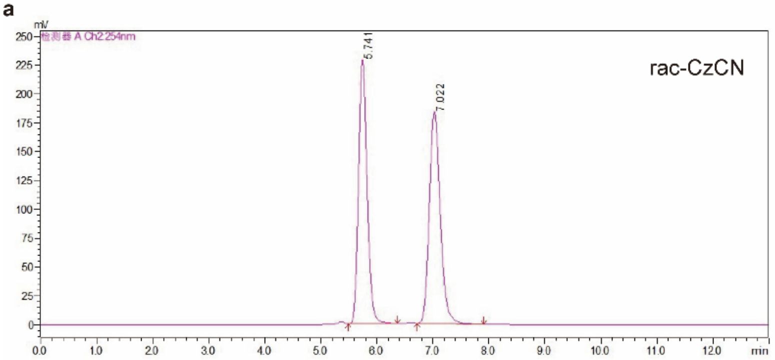

Peak#	Ret. Time	Area	Area%
1	5.741	2448750	49.4386
2	7.022	2504361	50.5614

b

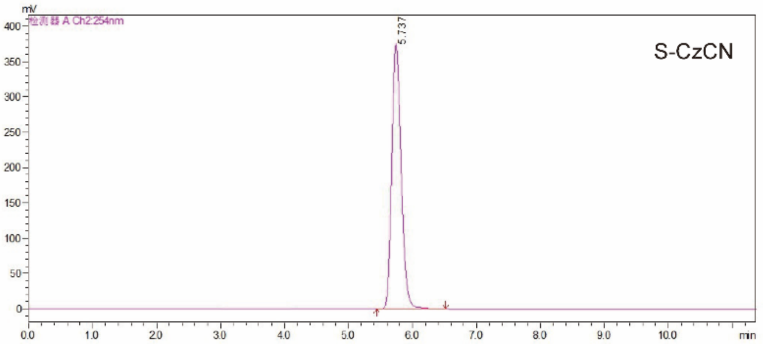

Peak#	Ret. Time	Area	Area%
1	5.737	3860035	100.0000

c

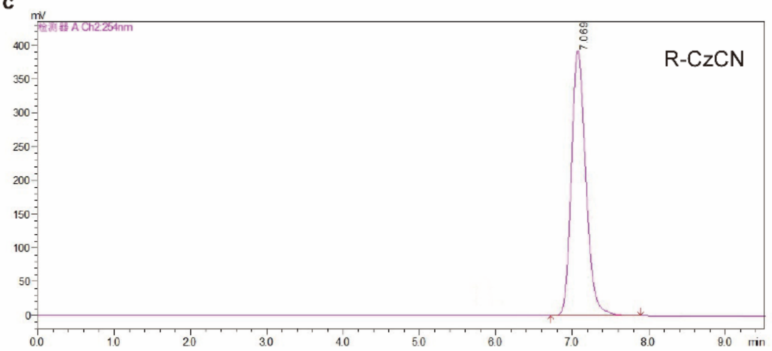

Peak#	Ret. Time	Area	Area%
2	7.069	5179901	100.0000

Supplementary Fig. 48. HPLC chromatograms of CzCN. (**a**) rac-CzCN. (**b**) S-CzCN. (**c**) R-CzCN.

a

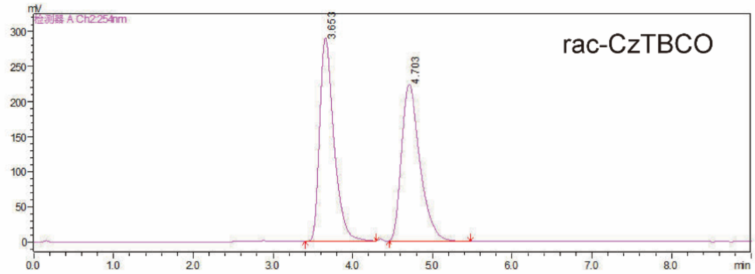

Peak#	Ret. Time	Area	Area%
1	3.653	3612880	50.2280
2	4.703	3580084	49.7720

b

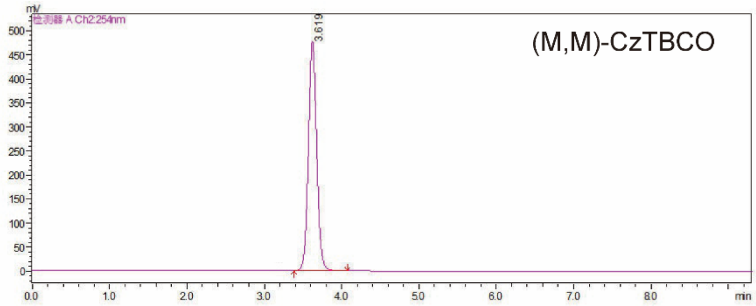

Peak#	Ret. Time	Area	Area%
1	3.619	3560151	100.0000

c

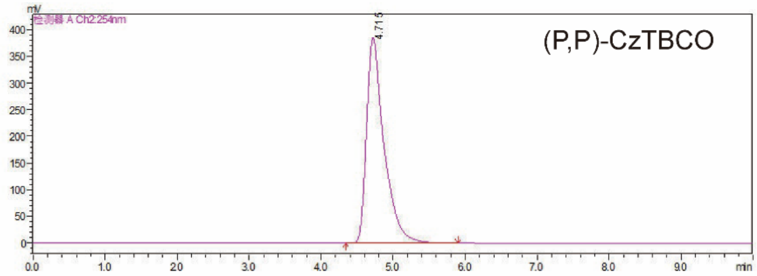

Peak#	Ret. Time	Area	Area%
2	4.715	6539983	100.0000

Supplementary Fig. 49. HPLC chromatograms of CzTBCO. (**a**) rac-CzTBCO. (**b**) S-CzTBCO. (**c**) R-CzTBCO.

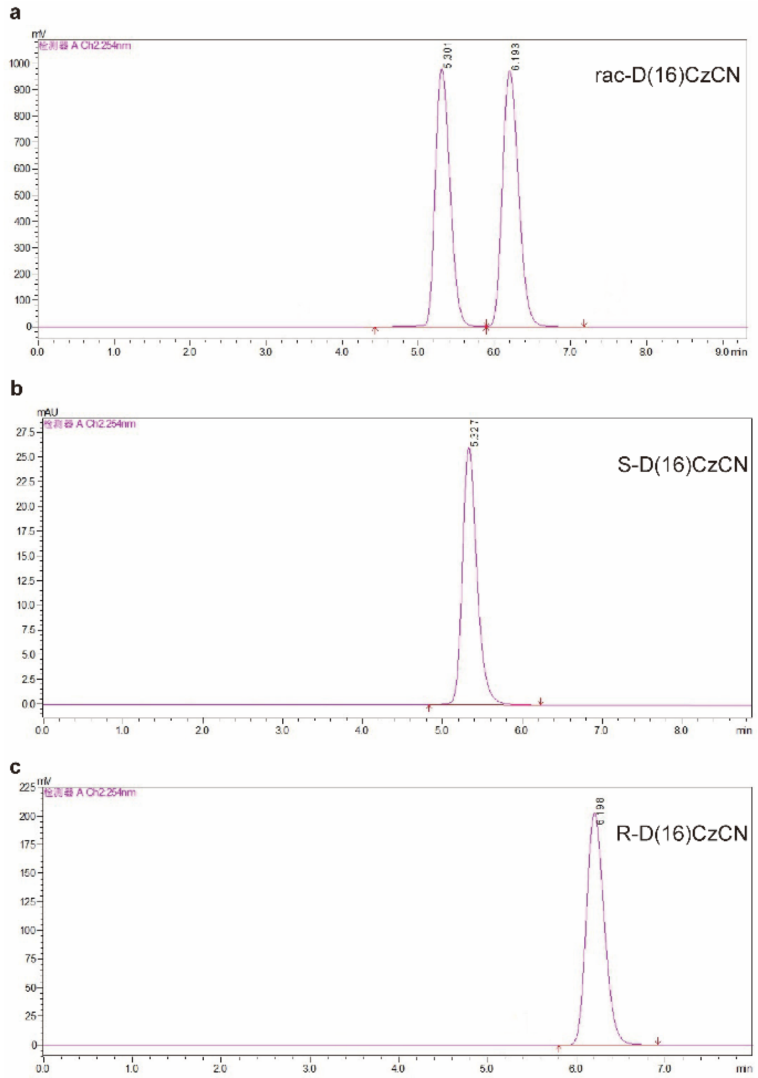

Peak#	Ret. Time	Area	Area%
1	5.301	13249127	48.0241
2	6.193	14339350	51.9759

Peak#	Ret. Time	Area	Area%
1	5.327	331288	100.0000

Peak#	Ret. Time	Area	Area%
2	6.198	2839707	100.0000

Supplementary Fig. 50. HPLC chromatograms of D(16)CzCN. (**a**) rac-D(16)CzCN. (**b**) S-D(16)CzCN. (**c**) R-CzCN.

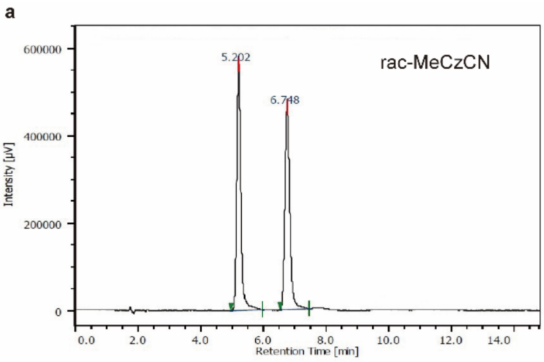

Peak#	Ret. Time	Area	Area%
1	5.202	5125096	50.291
2	6.748	5065849	49.709

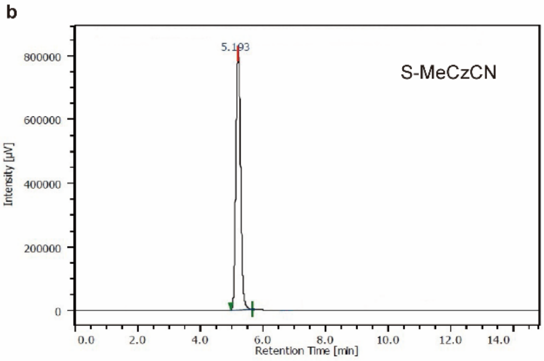

Peak#	Ret. Time	Area	Area%
1	5.193	8485247	100.0000

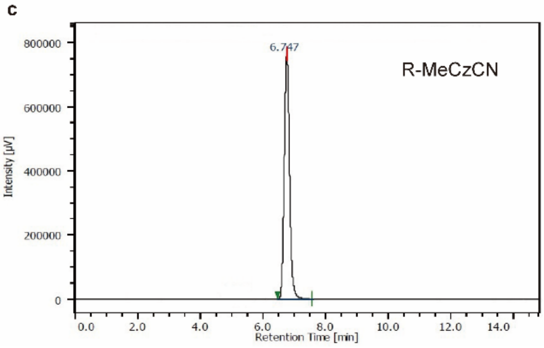

Peak#	Ret. Time	Area	Area%
2	6.747	8950863	100.0000

Supplementary Fig. 51. HPLC chromatograms of MeCzCN. (**a**) rac-MeCzCN. (**b**) S-MeCzCN. (**c**) R-MeCzCN.

a

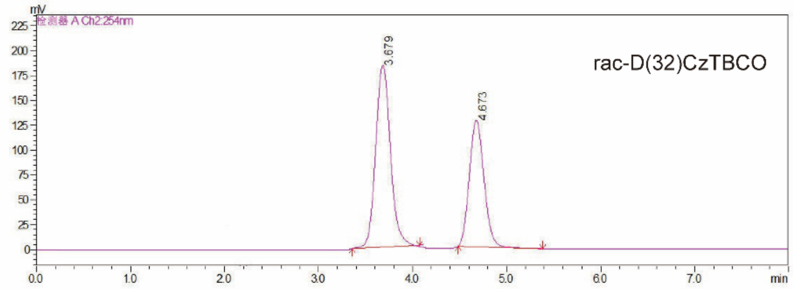

Peak#	Ret. Time	Area	Area%
1	3.679	2001311	59.1095
2	4.673	1384456	40.8905

b

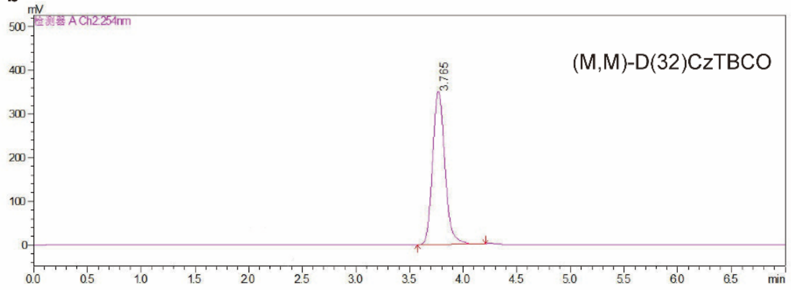

Peak#	Ret. Time	Area	Area%
1	3.765	2772412	100.0000

c

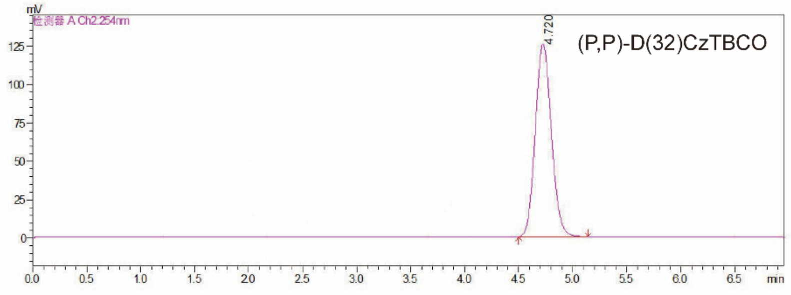

Peak#	Ret. Time	Area	Area%
2	4.720	1301684	100.0000

Supplementary Fig. 52. HPLC chromatograms of D(32)CzTBCO. (**a**) rac-D(32)CzTBCO. (**b**) S-D(32)CzTBCO. (**c**) R-D(32)CzTBCO.

Supplementary Tables

Supplementary Table 1. Absolute *Φ* values of R/S-CzCN neat films measured in air and under vacuum conditions at room temperature with different excitation wavelengths.

	250 nm	270 nm	290 nm	310 nm	330 nm	350 nm	370 nm
**Air**	61.2%	60.9%	57.2%	54.3%	50.5%	49.2%	46.5%
**Vacuum**	62.8%	62.2%	60.6%	58.9%	56.5%	55.8%	54.3%

Supplementary Table 2. Physical data and kinetic parameters of R/S-D(16)CzCN, R/S-CzCN and R/S-MeCzCN in undoped film.

	PLQY[a] / %	Prompt PLQY[b] / %	Delayed PLQY[c] / %	τ_{PF}[d] / ns	τ_{DF}[e] / µs	$k_{r,S}$[f] / × 10^7 s^{-1}	k_{ISC}[g] / × 10^7 s^{-1}	k_{RISC}[h] / × 10^5 s^{-1}	$k_{nr,S}$[i] / × 10^6 s^{-1}
**R/S-D(16)CzCN**	89.6 ± 1.1	31.8 ± 0.4	57.8 ± 0.7	23.5	4.5	1.4	2.7	6.2	1.6
**R/S-CzCN**	63.6 ± 2.3	15.8 ± 0.6	47.8 ± 1.7	24.8	4.2	0.6	2.9	9.4	3.7
**R/S-MeCzCN**	42.0 ± 1.6	11.8 ± 0.5	30.2 ± 1.1	23.1	4.7	0.5	3.0	7.5	7.1

[a]Photoluminescence quantum yield. [b]Prompt fluorescence component in the PLQY. [c]Delayed fluorescence component in the PLQY. [d]Prompt fluorescence lifetime. [e]Delayed fluorescence lifetime. [f]Rate constant of singlet radiative decay. [g]Rate constant of intersystem crossing process. [h]Rate constant of reverse intersystem crossing process. [i]Rate constant of singlet nonradiative decay.

Supplementary Table 3. Energy levels of the S_1 and S_2 states, S_1-S_2 splitting and excitonic coupling strength for R-D(16)CzCN, and R-CzCN.

R-D(16)CzCN	Energy (eV)	S_1	3.9132
		S_2	3.9235
	Energy splitting (eV)	$\Delta E_{S2\text{-}S1}$	0.0103
	Excitonic coupling strength (eV)	J	0.0052
R-CzCN	Energy (eV)	S_1	3.9157
		S_2	3.9259
	Energy splitting (eV)	$\Delta E_{S2\text{-}S1}$	0.0102
	Excitonic coupling strength (eV)	J	0.0051

Supplementary Table 4. Maximum and average g_{PL} values for R/S-MeCzCN, R/S-CzCN, and R/S-D(16)CzCN across various solvents, measured within the 430-580 nm spectral range.

Molecule	Solvent	Max g_{PL}[a]	Average g_{PL}[a]	Max g_{PL}[b]	Average g_{PL}[b]
**R/S-MeCzCN**	hexane	2.9×10^{-3}	1.5×10^{-3}	-2.8×10^{-3}	-1.8×10^{-3}
	toluene	2.2×10^{-3}	1.7×10^{-3}	-2.5×10^{-3}	-1.9×10^{-3}
	dichloromethane	2.3×10^{-3}	1.7×10^{-3}	-2.9×10^{-3}	-1.9×10^{-3}
	methanol	4.3×10^{-3}	1.8×10^{-3}	-3.6×10^{-3}	-1.7×10^{-3}
**R/S-CzCN**	hexane	4.2×10^{-3}	3.3×10^{-3}	-4.5×10^{-3}	-3.1×10^{-3}
	toluene	4.3×10^{-3}	3.3×10^{-3}	-4.8×10^{-3}	-3.3×10^{-3}
	dichloromethane	4.1×10^{-3}	3.3×10^{-3}	-4.0×10^{-3}	-3.3×10^{-3}
	methanol	3.9×10^{-3}	2.9×10^{-3}	-4.2×10^{-3}	-3.1×10^{-3}
**R/S-D(16)CzCN**	hexane	7.5×10^{-3}	6.0×10^{-3}	-8.0×10^{-3}	-5.8×10^{-3}
	toluene	7.1×10^{-3}	6.1×10^{-3}	-7.3×10^{-3}	-5.8×10^{-3}
	dichloromethane	8.5×10^{-3}	5.9×10^{-3}	-8.0×10^{-3}	-6.1×10^{-3}
	methanol	7.1×10^{-3}	5.4×10^{-3}	-1.0×10^{-2}	-5.5×10^{-3}

[a]Maximum g_{PL} values and average g_{PL} values for the R-enantiomer. [b]Maximum g_{PL} values and average g_{PL} values for the S-enantiomer.

Supplementary Table 5. Comparison of bond lengths, bond angles, and dihedral angles between R-CzCN and R-D(16)CzCN.

	Atom1	Atom2	Atom3	Atom4	R-CzCN	R-D(16)CzCN
Bond #1	1	2	-	-	1.4410 Å	1.4366 Å
Bond #2	2	3	-	-	1.3909 Å	1.3896 Å
Bond #3	3	4	-	-	1.3955 Å	1.3954 Å
Bond #4	4	5	-	-	1.3929 Å	1.3931 Å
Bond #5	5	6	-	-	1.3924 Å	1.3928 Å
Bond #6	6	1	-	-	1.4425 Å	1.4394 Å
Bond #7	7	8	-	-	1.4471 Å	1.4454 Å
Bond #8	8	9	-	-	1.4096 Å	1.4094 Å
Bond #9	9	10	-	-	1.3744 Å	1.3744 Å
Bond #10	10	11	-	-	1.4130 Å	1.4140 Å
Bond #11	11	12	-	-	1.3815 Å	1.3803 Å
Bond #12	12	7	-	-	1.4381 Å	1.4341 Å
Angle #1	1	2	3	-	122.345°	122.497°
Angle #2	2	3	4	-	121.437°	121.128°
Angle #3	3	4	5	-	118.093°	118.251°

Angle #4	4	5	6	-	121.429^{o}	121.422^{o}
Angle #5	5	6	1	-	122.218^{o}	122.003^{o}
Angle #6	6	1	2	-	113.894^{o}	114.194^{o}
Angle #7	7	8	9	-	121.354^{o}	121.060^{o}
Angle #8	8	9	10	-	120.441^{o}	120.474^{o}
Angle #9	9	10	11	-	119.043^{o}	119.212^{o}
Angle #10	10	11	12	-	122.188^{o}	121.832^{o}
Angle #11	11	12	7	-	120.258^{o}	120.435^{o}
Angle #12	12	7	8	-	115.813^{o}	116.113^{o}
Dihedral #1	1	2	3	4	1.48^{o}	1.23^{o}
Dihedral #2	2	3	4	5	3.88^{o}	3.67^{o}
Dihedral #3	3	4	5	6	2.89^{o}	2.61^{o}
Dihedral #4	4	5	6	1	3.49^{o}	3.37^{o}
Dihedral #5	5	6	1	2	8.26^{o}	7.73^{o}
Dihedral #6	6	1	2	3	7.27^{o}	6.70^{o}
Dihedral #7	6	1	7	8	46.9^{o}	47.5^{o}
Dihedral #8	7	8	9	10	3.75^{o}	3.81^{o}

Dihedral #9	8	9	10	11	3.10°	3.00°
Dihedral #10	9	10	11	12	3.03°	3.08°
Dihedral #11	10	11	12	7	3.98°	3.74°
Dihedral #12	11	12	7	8	10.23°	10.02°
Dihedral #13	12	7	8	9	10.23°	10.14°

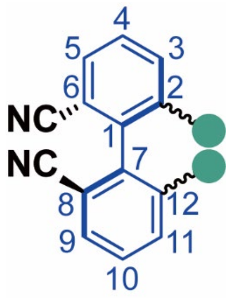

Supplementary Table 6. Atomic coordinates of R-MeCzCN.

C	0.176421	0.999884	-0.711652		C	6.455936	1.192873	-0.805152
C	1.362421	0.492961	-1.34845		H	5.857178	2.804916	-2.10094
C	1.489619	0.41172	-2.728968		H	6.744583	-0.485092	0.54342
C	0.476609	0.858842	-3.581751		N	-2.441849	0.088232	0.484545
C	-0.638123	1.465838	-3.011827		C	-3.712832	0.546602	0.851898
C	-0.779012	1.57339	-1.629234		C	-2.617834	-0.907747	-0.479331
C	-0.071116	0.929932	0.709567		C	-4.067807	1.551833	1.754125
C	0.935005	1.332704	1.667064		C	-4.69718	-0.143613	0.12456
C	0.817724	1.011114	3.034906		C	-1.68669	-1.67682	-1.18141
C	-0.299817	0.365125	3.506242		C	-3.99746	-1.07975	-0.72813
C	-1.355278	0.081457	2.61123		C	-5.41432	1.830379	1.920139
C	-1.279385	0.38286	1.26474		H	-3.31001	2.113214	2.299394
H	2.391287	-0.04089	-3.144539		C	-6.05045	0.15938	0.308652
H	0.577907	0.766047	-4.662441		C	-2.14852	-2.61227	-2.09694
H	-1.408694	1.905654	-3.647636		H	-0.61801	-1.5261	-1.05984
H	1.616379	1.313188	3.715087		C	-4.43574	-2.02459	-1.65909
H	-0.394165	0.107466	4.561803		C	-6.42386	1.146467	1.211569
H	-2.265506	-0.392699	2.980016		H	-5.70493	2.620068	2.618196
C	2.000182	2.187537	1.278471		H	-6.81373	-0.37731	-0.26072
N	2.872686	2.894816	0.979729		C	-3.51841	-2.80775	-2.35117
C	-1.834782	2.408453	-1.144035		H	-1.41777	-3.2078	-2.65098
N	-2.660494	3.146107	-0.806775		H	-5.5061	-2.14743	-1.84362
N	2.486903	0.008446	-0.605741		C	7.906001	1.573679	-0.79769
C	2.554415	-1.073155	0.272073		H	8.196652	1.961424	0.192181
C	3.759651	0.508644	-0.789001		H	8.547005	0.702759	-1.00241
C	1.544595	-1.90649	0.746225		H	8.127001	2.350219	-1.541
C	3.902708	-1.275722	0.652908		C	3.587358	-4.26953	2.945488
C	4.165392	1.623718	-1.530348		H	4.363301	-4.91955	2.512431
C	4.685501	-0.249128	-0.030954		H	3.993414	-3.86744	3.887265
C	1.904341	-2.940679	1.60879		H	2.716899	-4.89138	3.19076
H	0.504213	-1.754532	0.479992		C	-3.96354	-3.83684	-3.35767
C	4.236469	-2.305174	1.505638		H	-5.05714	-3.84465	-3.4657
C	5.517492	1.937028	-1.53353		H	-3.53108	-3.64213	-4.35194
H	3.442223	2.237636	-2.062302		H	-3.65044	-4.85137	-3.06314
C	6.020013	0.086696	-0.040656		C	-7.87092	1.502309	1.432834
C	3.226894	-3.157976	2.003513		H	-8.536	0.882345	0.815342
H	1.119819	-3.594311	1.993581		H	-8.1643	1.362409	2.485489
H	5.276518	-2.469678	1.796981		H	-8.06725	2.556662	1.181129

Supplementary Table 7. Atomic coordinates of R-CzCN.

C	-0.152017	0.794243	-0.742852		C	-5.468548	1.698772	-1.593439
C	-1.332628	1.36575	-0.160999		H	-3.398038	2.313218	-1.769686
C	-1.457154	2.724441	0.096003		C	-5.957896	-0.084154	0.004708
C	-0.435396	3.617295	-0.233365		C	-3.11303	-2.534885	2.894424
C	0.683474	3.121993	-0.896853		H	-1.010701	-2.554862	3.36279
C	0.816904	1.76433	-1.180296		H	-5.172923	-2.302724	2.245219
C	0.074811	-0.62667	-0.834421		C	-6.373984	0.835132	-0.973452
C	-0.944338	-1.519149	-1.337754		H	-5.817752	2.377662	-2.371876
C	-0.866013	-2.909916	-1.119231		H	-6.675513	-0.769045	0.458779
C	0.224117	-3.456343	-0.486002		H	-3.345187	-3.351925	3.579218
C	1.29709	-2.615058	-0.111232		H	-7.424674	0.861148	-1.266775
C	1.254786	-1.249635	-0.309011		N	2.416443	-0.513088	0.069193
H	-2.357646	3.081848	0.597676		C	3.683487	-0.819663	-0.437149
H	-0.528426	4.677627	-0.002237		C	2.574538	0.416978	1.096964
H	1.466676	3.802168	-1.235391		C	4.030088	-1.66786	-1.48888
H	-1.675373	-3.544632	-1.484287		C	4.65876	-0.08042	0.259479
H	0.284826	-4.530188	-0.306552		C	1.62969	1.045154	1.910945
H	2.187887	-3.039679	0.352643		C	3.951378	0.712239	1.242977
C	-1.988451	-1.023954	-2.159058		C	5.374188	-1.77696	-1.81458
N	-2.848812	-0.623783	-2.830391		H	3.269114	-2.21149	-2.04717
C	1.89603	1.371398	-2.032487		C	6.006753	-0.20363	-0.08572
N	2.748178	1.11159	-2.770656		C	2.073172	1.951689	2.865756
N	-2.446562	0.560279	0.223066		H	0.565086	0.87871	1.782788
C	-2.493693	-0.479783	1.155028		C	4.375301	1.627734	2.206913
C	-3.718437	0.788759	-0.257824		C	6.358904	-1.05737	-1.12031
C	-1.467772	-1.048108	1.905601		H	5.668891	-2.42976	-2.63886
C	-3.83172	-0.924688	1.273233		H	6.768626	0.368228	0.448389
C	-4.121094	1.682702	-1.257714		C	3.434045	2.244299	3.020107
C	-4.626178	-0.107173	0.360116		H	1.338	2.454154	3.497927
C	-1.801661	-2.085771	2.776875		H	5.437911	1.855269	2.315324
H	-0.437551	-0.727758	1.804565		H	7.407056	-1.16486	-1.40561
C	-4.145321	-1.950442	2.141526		H	3.751878	2.964241	3.776487

Supplementary Table 8. Atomic coordinates of R-D(16)CzCN.

C	-0.14965	0.80494	-0.73674		C	-5.48638	1.69943	-1.56274
C	-1.34086	1.36351	-0.15973		H(Iso=2)	-3.41747	2.31214	-1.75726
C	-1.47884	2.72174	0.09942		C	-5.96163	-0.092911	0.02775
C	-0.46172	3.6233	-0.2163		C	-3.09936	-2.56597	2.87599
C	0.66869	3.13929	-0.8709		H(Iso=2)	-0.99326	-2.59333	3.32754
C	0.81663	1.78465	-1.15908		H(Iso=2)	-5.16306	-2.3226	2.24494
C	0.07915	-0.61322	-0.84658		C	-6.38682	0.832909	-0.93924
C	-0.94042	-1.49734	-1.36423		H(Iso=2)	-5.8422	2.383109	-2.334
C	-0.86677	-2.8901	-1.16095		H(Iso=2)	-6.67457	-0.781301	0.48393
C	0.22067	-3.44595	-0.53058		H(Iso=2)	-3.32834	-3.38586	3.55846
C	1.29534	-2.61204	-0.14461		H(Iso=2)	-7.4403	0.861749	-1.2222
C	1.25724	-1.24436	-0.32655		N	2.4182	-0.51403	0.06399
H	-2.38529	3.06968	0.59683		C	3.6866	-0.81603	-0.44182
H	-0.56439	4.68168	0.01956		C	2.57347	0.40654	1.10102
H	1.44911	3.82767	-1.19915		C	4.03588	-1.65401	-1.5008
H	-1.67638	-3.51896	-1.53543		C	4.65991	-0.08329	0.26422
H	0.27803	-4.52179	-0.36228		C	1.6267	1.02655	1.91918
H	2.1837	-3.04448	0.31677		C	3.94995	0.70027	1.25314
C	-1.97779	-0.98785	-2.18586		C	5.38083	-1.75985	-1.82416
N	-2.83089	-0.57369	-2.85774		H(Iso=2)	3.2765	-2.19246	-2.06625
C	1.90674	1.40647	-2.00399		C	6.00875	-0.203	-0.07875
N	2.76816	1.15992	-2.73588		C	2.06812	1.92383	2.88366
N	-2.44611	0.548	0.21783		H(Iso=2)	0.56228	0.86028	1.78861
C	-2.48684	-0.50277	1.14193		C	4.3717	1.60651	2.2267
C	-3.72402	0.78117	-0.24896		C	6.36364	-1.04681	-1.12055
C	-1.45767	-1.07796	1.88202		H(Iso=2)	5.67772	-2.40475	-2.65386
C	-3.82446	-0.94457	1.26902		H(Iso=2)	6.76916	0.363969	0.46259
C	-4.13564	1.68008	-1.24038		C	3.42859	2.21528	3.04358
C	-4.62601	-0.11875	0.37025		H(Iso=2)	1.33156	2.41964	3.51944
C	-1.78773	-2.11939	2.75022		H(Iso=2)	5.43407	1.832889	2.33972
H(Iso=2)	-0.4271	-0.76075	1.77569		H(Iso=2)	7.41252	-1.15142	-1.40422
C	-4.13475	-1.97436	2.13466		H(Iso=2)	3.74467	2.92796	3.80753

Supplementary Table 9. Features of the reported CP-OLEDs.

Year	Emitting materials[ref]	CE / $cd \cdot A^{-1}$	PE / $lm \cdot W^{-1}$	EQE / %	g_{EL} / × 10^{-3}	Device structure	CP-EL detection
**2023**	**This work (BN2)**	**145.9**	**163.7**	**34.6**	**3.3**	**ITO/HATCN/TAPC/TCTA/mCBP/R/S-D(16)CzCN:BN2/POT2T/ANT-BIZ/Liq/Al**	**CPL-300**
2023	4CzTPNBu[1]	66.9	72.4	20.4	2.9	ITO/HATCN/TAPC/TCTA/mCP/R/S-BNPCN-*p*-CP:4CzTPNBu/PPF/TmPyPB/LiF/Al	CPL-300
2023	$Ir(tptpy)_2acac$[1]	103.3	120.1	29.3	2.3	ITO/HATCN/TAPC/TCTA/mCP/R/S-BNPCN-*p*-CP:$Ir(tptpy)_2acac$/PPF/TmPyPB/LiF/Al	CPL-300
2023	BN3[1]	89.6	104.1	23.8	2.7	ITO/HATCN/TAPC/TCTA/mCP/R/S-BNPCN-*p*-CP:BN3/PPF/TmPyPB/LiF/Al	CPL-300
2023	Ir1[2]	115.7	107.0	32.0	3.2	ITO/TAPC/TCTA/TCTA:R/S-TRZOBN:Ir1/TmPyPB/Liq/Al	CPL-300
2023	$Ir(mppy)_3$[3]	61.0	Not reported	17.1	1.3	ITO/PEDOT:PSS/R/S-BN-2mCP:$Ir(mppy)_3$/DPEPO/TmPyPB/LiF/Al	CPL-200
2022	BN1[4]	103.7	100.3	33.2	2.8	ITO/HATCN/TAPC/mCP/R/S-CzOBN:POT2T:BN1/POT2T/TmPyPB/Liq/Al	CPL-300
2023	R/S-ax-DMAC[5]	87.3	64.7	30.1	2.0	ITO/NPB/TAPC/mCBP:R/S-ax-DMAC/PPT/TmPyPB/LiF/Al	CPL-300
2023	R/S-Ax-CN[6]	64.0	43.8	21.0	4.2	ITO/TAPC/mCP/35DczPPy:R/S-Ax-CN/PPT/TPBi/LiF/Al	CPL-300
2023	(P,P)/(M,M)-CNSPZ[7]	58.0	52.2	20.0	2.9	ITO/TAPC/TCTA/mCBP:(P,P)/(M,M)-CNSPZ/B3PyMPM/LiF/AL	CPL-200
2023	R/S-Czp-tBuCzB[8]	38.7	35.9	32.1	1.5	ITO/HATCN/TAPC/2,6DczPPy:R/S-Czp-tBuCzB/TmPyPb/LiF/Al	CPL-300
2023	R/S-$(BINAP)_2Cu(\mu\text{-}I_2)$[9]	78.0	57.0	21.7	3.0	ITO/MoO_3/TAPC/2,6Dczppy:R/S-$(BINAP)_2Cu(\mu\text{-}I_2)$/TmPyPB/LiF/Al	CPL-300

2022	R/S-DOBNT[10]	24.1	17.2	25.6	1.0	ITO/HATCN/TAPC/2,6-DCzPPy:R/S-DOBNT/TmPyPb/LiF/Al	CPL-300
2022	R/S-SDMAC[11]	60.4	35.5	28.4	0.8	ITO/HATCN/TAPC/TCTA/mCBP/PPF:R/S-SDMAC/PPF/TmPyPB/Liq/Al	CPL-300
2022	R/S-BN-MeIAc[12]	103.0	130.2	37.2	0.3	ITO/HATCN/TAPC/TCTA/mCBP/DMIC-TRZ:R/S-BN-MeIAc/POT2T/ANT-BIZ/Liq/Al	CPL-300
2022	R/S-SCFPY[13]	67.5	44.2	23.3	3.6	ITO/HATCN/TAPC/26DCzPPy:R/S-SCFPY/TmPyPB/LiF/Al	CPL-300
2022	R/S-OSFSO/R/S-SPOCN[14]	51.6	40.5	20.4	3.0	ITO/HATCN/TAPC/TCTA:R/S-OSFSO/R/S-SPOCN/PPF:R/S-OSFSO/R/S-SPOCN/PPF/TmPyPB/LiF/Al	CPL-300
2022	P/M-QPO-PhCz[15]	25.0	30.5	10.6	1.6	ITO/HATCN/TAPC/TCTA/mCP/DPEPO:P/M-QPO-PhCz /DPEPO/TmPyPB/Liq/Al	CPL-300
2022	(R,R,R)/(S,S,S)-BINA-A[16]	28.7	11.0	14.3	1.9	ITO/PEDOT:PSS/TCTA:mCP:OXD-7:(R,R,R)/(S,S,S)-BINA-A/BmPyPb/LiF/Al	CPL-300
2022	R/S-OBN-AICz[17]	55.9	47.5	19.0	0.5	ITO/TAPC/mCBP/mCBP:R/S-OBN-AICz/TmPyPB/LiF/Al	CPL-300
2022	(P,P″,P″)/(M,M″,M″)-BN4[18]	66.3	38.6	20.6	3.7	ITO/PEDOT:PSS/PVK/mCPCN:(P,P″,P″)/(M,M″,M″)-BN4/DPEPO/TmPyPB/LiF/Al	CPL-300
2021	R/S-OSFSO[19]	38.1	21.8	20.0	3.1	ITO/HATCN/TAPC/TCTA/TCTA:R/S-OSFSO/DPEPO:R/S-OSFSO/ DPEPO/TmPyPB/LiF/Al	CPL-300
2021	R/S-NID-MeIAc[20]	68.4	61.3	23.7	2.4	ITO/MoO_3/TAPC/mCP/mCPCN:R/S-NID-MeIAc/3TPYMB/LiF/Al	CPL-300
2021	R/S-OBN-2CN-BN[21]	71.9	79.8	29.8	1.4	ITO/TAPC/TCTA/PhCzBCz:5CzBN:R/S-OBN-2CN-BN/ TmPyPB/LiF/Al	CPL-200
2021	R/S-*o*-BAMCN[22]	12.2	7.6	4.3	4.6	ITO/HATCN/HATCN:TAPC/mCP:R/S-*o*-BAMCN/Tm3PyP26PyB/ LiF/Al/Ag	CPL-300

2021	R/S-P-BPCZ4[23]	37.4	23.0	18.3	5.5	ITO/HATCN/TAPC/mCP/mCP: R/S-P-BPCZ4/DPEPO: R/S-P-BPCZ4/TmPyPB/LiF/Al	CPL-300
2021	(R,R)-/(S,S)-CPAD[24]	67.9	68.2	20.4	1.8	ITO/TAPC/TCTA/PhCzTrz:PXZ-DPS:(R,R)-/(S,S)-CPAD/BPBiPA/LiF/Al	CPL-300
2021	Λ/Δ-Ir-(R-camphor)[25]	105.9	106.9	30.6	0.6	ITO/HATCN/TAPC:HATCN/TCTA:Λ/Δ-Ir-(R-camphor)/Tm3PyP26PyB/LiF/Al	CPL-300
2021	(R,R)-/(S,S)-pTpAcBP[26]	73.9	58.1	22.1	1.6	ITO/PEDOT:PSS/mCP: (R,R)-/(S,S)-pTpAcBP/TmPyPB/LiF/Al	CPL-300
2020	R/S-BPPOACZ[27]	61.0	42.6	17.8	4.5	ITO/HATCN/TAPC/26DCzPPy:R/S-BPPOACZ/TmPyPB/LiF/Al	CPL-300
2020	R/S-SFOT[28]	68.0	53.6	23.1	1.0	ITO/HATCN/TAPC/TCTA/mCBP:SFOT/TmPyPB/Liq/Al	CPL-300
2020	R/S-C’3[29]	2.5	Not reported	0.8	1.0	Al-Cu/TiN/Ca/Bphen/Alq3/mCP: R/S-C’3/STTB/STTB:F4TCNQ /Ag/SiO/Al_2O_3	CPL-300
2019	P/M-Pt[30]	22.5	18.6	18.8	1.6	ITO/HATCN/HATCN:TAPC/TCTA:P/M-Pt/26DCzPPy:P/M-Pt/Tm3PyP26PyB/LiF/Al	CPL-300
2019	R/S-BPO-PXZ[31]	4.2	Not reported	1.8	1.0	ITO/PEDOT:PSS/TCTA:R/S-BPO-PXZ/TPBi/Ca/Ag	CPL-300
2019	R/S-OBN-Cz[32]	93.7	59.3	32.6	1.9	ITO/HATCN/TAPC/TCTA:R/S-OBN-Cz/26DCzPPy:R/S-OBN-Cz/TmPyPB/LiF/Al	CPL-300
2019	R/S-OBN-DPA[33]	45.3	30.6	12.4	2.3	ITO/HATCN/TAPC/TCTA:R/S-OBN-DPA/26DCzPPy:R/S-OBN-DPA/TmPyPB/LiF/Al	CPL-300
2019	R/S-CPDCB[34]	39.5	30.3	12.4	0.9	ITO/PEDOT:PSS/TAPC/mCP:R/S-CPDCB/TmPyPB/LiF/Al	CPL-300
2018	(-)-(R,R)-CAI-Cz[35]	59.0	53.0	19.7	1.7	ITO/HATCN/TAPC/TCTA/mCBP/mCBP:(-)-(R,R)-CAI-Cz/TmPyPB/Liq/Al	CPL-200
2021	(S,S)-(+)-TpAc-TRZ[36]	61.2	64.1	17.5	1.5	ITO/PEDOT:PSS/(S,S)-(+)-TpAc-TRZ/TPBi/LiF/Al	QWP/LP[a]

2020	R/S-Cz-Ax-CN[37]	20.6	15.4	12.5	12	ITO/HATCN/TAPC/mCP/DPEPO: R/S-Cz-Ax-CN/TSPO1/TPBi/LiF/Al	QWP/LP
2018	R/S-BN-CF[38]	19.6	24.6	9.3	60	ITO/HATCN/TAPC:HATCN/TCTA/mCP:R/S-BN-CF/BmPyPB/BmPyPB:Liq/Liq/Al	QWP/LP

[a]Quarter-wave plate/linear polarizer

Supplementary Table 10. Maximum and average g_{PL} values for (P,P)/(M,M)-CzTBCO, and (P,P)/(M,M)-D(32)CzTBCO across various solvents, measured within the 480-600 nm spectral range.

Molecule	Solvent	Max g_{PL}[a]	Average g_{PL}[a]	Max g_{PL}[b]	Average g_{PL}[b]
**(P,P)/(M,M)-CzTBCO**	hexane	9.1×10^{-3}	8.0×10^{-3}	-8.9×10^{-3}	-7.8×10^{-3}
	toluene	8.6×10^{-3}	7.5×10^{-3}	-8.6×10^{-3}	-7.5×10^{-3}
	dichloromethane	6.4×10^{-3}	5.3×10^{-3}	-6.4×10^{-3}	-5.4×10^{-3}
	methanol	8.1×10^{-3}	6.8×10^{-3}	-8.7×10^{-3}	-6.9×10^{-3}
**(P,P)/(M,M)-D(32)CzTBCO**	hexane	1.2×10^{-2}	1.1×10^{-2}	-1.2×10^{-2}	-1.1×10^{-2}
	toluene	1.1×10^{-2}	9.2×10^{-3}	-1.1×10^{-2}	-9.1×10^{-3}
	dichloromethane	8.0×10^{-3}	6.6×10^{-3}	-8.3×10^{-3}	-6.7×10^{-3}
	methanol	9.5×10^{-3}	7.9×10^{-3}	-9.4×10^{-3}	-7.9×10^{-3}

[a]Maximum g_{PL} values and average g_{PL} values for the (P,P)-enantiomer. [b]Maximum g_{PL} values and average g_{PL} values for the (M,M)-enantiomer.

Supplementary Table 11. Summary of the calculated data for absolute configuration (*R* or *P*).

Molecule	R^{a}	g^{a}	$\bar{R}^{b}$	$\bar{g}^{b}$	g_{cal} reduction[c]	g_{PL} *increment*[d]
R_p-CzpPhTrz	-5.0 × 10^{-39}	-8.5 × 10^{-4}	-4.5 × 10^{-39}	-8.1 × 10^{-4}	4.7%	-
(-)-(*R*,*R*)-CAI-Cz	1.7 × 10^{-39}	3.7 × 10^{-3}	1.5 × 10^{-39}	3.3 × 10^{-3}	10.8%	-
(*R*)-OBN-Cz	-7.6 × 10^{-40}	-6.6 × 10^{-4}	-5.0 × 10^{-40}	-4.6 × 10^{-4}	30.3%	-
(*P*)-Aza[6]helicene	-1.1 × 10^{-38}	-1.0 × 10^{-2}	-1.0 × 10^{-38}	-7.7 × 10^{-3}	23.0%	-
**R-CzCN**	**1.6 × 10^{-39}**	**1.2 × 10^{-2}**	**7.8 × 10^{-40}**	**3.4 × 10^{-3}**	**71.7%**	**2.0-fold**
**(P,P)-CzTBCO**	**2.8 × 10^{-38}**	**1.5 × 10^{-2}**	**2.1 × 10^{-38}**	**1.0 × 10^{-2}**	**33.3%**	**1.3-fold**

[a]Rotatory strengths (*R*, in units of $esu^2 \cdot cm^2$), and *g*-factors calculated without considering vibrational effects using stationary-point TD-DFT calculations. [b]Rotatory strengths ($\bar{R}$, in units of $esu^2 \cdot cm^2$), and *g*-factors ($\bar{g}$) calculated with considering vibrational effects using nuclear ensemble approach. [c]g_{cal} reduction is defined as the reduction in *g*-factor due to molecular vibrations divided by the *g*-factor calculated without considering vibrational effects (from stationary-point TD-DFT calculations). [d]g_{PL} increment is defined as the enhancement factor of g_{PL} after deuteration.

Supplementary Table 12. The specific rotation of new chiral compounds.

Molecule	$[\alpha]_D^{25}$
R-MeCzCN	+76° (*c* = 0.30 g/100 mL, $CHCl_3$)
S-MeCzCN	-73° (*c* = 0.30 g/100 mL, $CHCl_3$)
R-D(16)CzCN	+91° (*c* = 0.70 g/100 mL, $CHCl_3$)
S-D(16)CzCN	-89° (*c* = 0.70 g/100 mL, $CHCl_3$)
(P,P)-D(32)CzTBCO	+32° (*c* = 0.20 g/100 mL, $CHCl_3$)
(M,M)-D(32)CzTBCO	-30° (*c* = 0.20 g/100 mL, $CHCl_3$)

Supplementary Notes

Supplementary Note 1. Orbital contribution to the magnetic dipole transition moment.

The g-factor, a dimensionless parameter quantifying the CPL magnitude, can be estimated as follows:

$$g = \frac{4R}{D} = \frac{4\langle \Psi_{S_1}|\boldsymbol{\mu}|\Psi_{S_0}\rangle \cdot \left\langle \Psi_{S_1}\left|\left(\frac{i}{2c}\right)\mathbf{m}\right|\Psi_{S_0}\right\rangle}{D} = \frac{4\langle \Psi_{S_1}|-e\mathbf{r}|\Psi_{S_0}\rangle \cdot \left\langle \Psi_{S_1}\left|\left(\frac{-ie}{2m_e \cdot c}\right)\mathbf{L} + g_e\mathbf{S}\right|\Psi_{S_0}\right\rangle}{D} \quad (1)$$

where e, the elementary charge of an electron; **r**, the position operator; m_e is the mass of the electron; c, the speed of light; **L**, orbital angular momentum; $g_e \approx 2$, the free electron g-factor; **S**, spin angular momentum. The term (**S**) specifically represents the spin contribution to the magnetic dipole transition moment. From the Supplementary Equation 1, it is clear that the orbital contribution (**L**) is positively correlated with the g-factor. This parameter (**L**) is intimately linked to rotating and overlapping between the transition orbitals, which can be modulated by molecular skeletal nuclear coordinates.

Supplementary Note 2. Analyses of rate constants.

The respective rate constants of the prompt and delayed fluorescence components (k_p and k_d) occurring in the CP-TADF emitter, can be given by:

$$k_p = \frac{k_{r,S} + k_{nr,S} + k_{ISC} + k_{r,T} + k_{nr,T} + k_{RISC}}{2} \times \left(1 + \sqrt{1 - \frac{4(k_{r,S} + k_{nr,S} + k_{ISC})(k_{r,T} + k_{nr,T} + k_{RISC}) - 4k_{ISC}k_{RISC}}{(k_{r,S} + k_{nr,S} + k_{ISC} + k_{r,T} + k_{nr,T} + k_{RISC})^2}}\right) \quad (2)$$

$$k_d = \frac{k_{r,S} + k_{nr,S} + k_{ISC} + k_{r,T} + k_{nr,T} + k_{RISC}}{2} \times \left(1 - \sqrt{1 - \frac{4(k_{r,S} + k_{nr,S} + k_{ISC})(k_{r,T} + k_{nr,T} + k_{RISC}) - 4k_{ISC}k_{RISC}}{(k_{r,S} + k_{nr,S} + k_{ISC} + k_{r,T} + k_{nr,T} + k_{RISC})^2}}\right) \quad (3)$$

k_p and k_d can be experimentally determined from prompt and delayed fluorescence decay time constants τ_p, τ_d as follows:

$$k_p = \frac{1}{\tau_p} \quad (4)$$

$$k_d = \frac{1}{\tau_d} \quad (5)$$

Besides, the emission quantum yields Φ_p and Φ_d for the prompt and delayed fluorescence components have the following relationship with these rate constants:

$$\Phi_p = \frac{k_{r,S}}{k_p} \frac{k_{r,S} + k_{nr,S} + k_{ISC} - k_d}{k_p - k_d} \quad (6)$$

$$\Phi_d = \frac{k_{r,S}}{k_d} \frac{k_p - k_{r,S} - k_{nr,S} - k_{ISC}}{k_p - k_d} \quad (7)$$

From the equations above, one could obtain the following relationship between rate constants and k_p, k_d, Φ_p and Φ_d experimentally determined from typical PLQY and transient PL characteristics:

$$k_{r,S} = \Phi_p k_p + \Phi_d k_d \quad (8)$$

$$k^S = k_{r,S} + k_{nr,S} + k_{ISC} = \frac{\Phi_p k_p^2 + \Phi_d k_d^2}{k_{r,S}} \quad (9)$$

$$k^T = k_{r,T} + k_{nr,T} + k_{RISC} = \frac{(\Phi_p + \Phi_d) k_p k_d}{k_{r,S}} \quad (10)$$

$$k_{ISC} k_{RISC} = \frac{\Phi_p \Phi_d k_p k_d (k_p - k_d)^2}{k_{r,S}^2} \quad (11)$$

Supplementary Note 3. Critical role of diastereomeric interactions.

Strong diastereomeric interactions are prerequisite for achieving efficient CP emission from achiral guest in chiral host and achiral guest systems. Three achiral MR-TADF emitters (BN2, DtBuCzB, and BNSeSe) were selected based on spectral overlap between their absorption and the host's emission. BN2 exists as a racemate with two enantiomers (*CC*-BN2 and *C*-BN2) exhibiting propeller chirality (twisted axial blades at ±36-48°) and helical chirality from its multiresonant framework (Supplementary Fig. 27). In contrast, DtBuCzB and BNSeSe feature distorted [4]helicene substructures, with BNSeSe showing greater distortion due to selenium incorporation (Supplementary Figs. 29 and 30).

Density functional theory (DFT) calculations (Supplementary Fig. 27) reveal an energetic difference of approximately 4.3 kcal·mol^{-1} between the R-D(16)CzCN:*CC*-BN2 (matched) and R-D(16)CzCN:*C*-BN2 (mismatched) complexes, thermodynamically favoring the former (*CC*-BN2 enantiomer) under Boltzmann distribution at 298 K (kT ≈ 0.6 kcal·mol^{-1}). This theoretical prediction is corroborated by experimental CD spectra (Supplementary Fig. 28). R/S-D(16)CzCN:BN2 films (2 wt%) exhibit distinct CD signals in the 400-500 nm range that is absent in the pristine R/S-D(16)CzCN neat films. The CD spectra of R/S-D(16)CzCN:BN2 in THF/water mixtures (V_{THF}:V_{water} = 10:90) further confirm this signal. In combination with FRET, the R/S-D(16)CzCN:BN2 system enables efficient CPL from the achiral BN2 emitter.

By contrast, DFT calculations indicate that the energetic difference between the R-D(16)CzCN:*P*-DtBuCzB (matched) and R-D(16)CzCN:*M*-DtBuCzB (mismatched) is minimal, approximately 0.2 kcal·mol^{-1} (Supplementary Fig. 29). Similarly, the energetic difference between the R-D(16)CzCN:*P*-BNSeSe (matched) and R-D(16)CzCN:*M*-BNSeSe (mismatched)

is *ca.* 0.7 kcal·mol^{-1} (Supplementary Fig. 30). These small energetic differences are insufficient to drive diastereomeric interactions at room temperature, assuming Boltzmann distribution at 298 K (kT ≈ 0.6 kcal.mol^{-1}). Accordingly, R/S-D(16)CzCN:DtBuCzB (2 wt%) and R/S-D(16)CzCN:BNSeSe (2 wt%) films display CD signals indistinguishable from pristine R/S-D(16)CzCN films, with no evidence of induced chirality in the guests. Despite the presence of FRET in these systems (Supplementary Fig. 31), their CPL signals remain weak (Supplementary Fig. 35), highlighting the necessity of strong diastereomeric interactions for efficient CPL generation in chiral host and achiral guest systems.

Supplementary Note 4. Mechanisms and rationale underlying CPL emission in CP-OLEDs based on R/S-D(16)CzCN as host.

The CPL emission in OLEDs employing R/S-D(16)CzCN as the chiral host arises from a synergistic interplay between two key processes: (i) Förster resonance energy transfer (FRET) from the chiral host to the achiral MR-TADF emitter, and (ii) diastereomeric interactions, which are host-guest interactions that energetically favor specific conformations of the emitter. The chiral host R/S-D(16)CzCN creates an chiral microenvironment around the emitter BN2. During FRET, dynamic structural features in BN2, such as its diphenylamine rotors, enhance these interactions, promoting a preferential chiral conformation of BN2 and thus leading to CPL emission from BN2. Conversely, emitters lacking such rotors (e.g., DtBuCzB and BNSeSe) exhibit weaker interaction with the host, resulting in markedly reduced CPL emission despite comparable energy transfer efficiency. These findings confirm that robust CPL in CP-OLEDs based on R/S-D(16)CzCN:BN2 requires both the deuteration of the chiral host to enhance its chiroptical properties, as well as the presence of dynamic structural motifs (e.g., rotors) in the achiral emitter, besides efficient FRET.

Supplementary References

1 Xu, L. et al. Efficient Circularly Polarized Electroluminescence from Achiral Luminescent Materials. *Angew. Chem. Int. Ed.* **62**, e202300492 (2023).

2 Chen, Z. et al. Cascade Chirality Transfer Through Diastereomeric Interaction Enables Efficient Circularly Polarized Electroluminescence. *Adv. Funct. Mater.* **33**, 2215179 (2023).

3 Dong, Q. et al. Binaphthol-based chiral host molecules for efficient solution-processed circularly polarized OLEDs. *Chem. Commun.* **59**, 1473-1476 (2023).

4 Chen, Z. et al. High-Performance Circularly Polarized Electroluminescence with Simultaneous Narrowband Emission, High Efficiency, and Large Dissymmetry Factor. *Adv. Mater.* **34**, 2109147 (2022).

5 Wan, S.-P. et al. Axially chiral thermally activated delayed fluorescence emitters enabled by molecular engineering towards high-performance circularly polarized OLEDs. *Chem. Eng. J.* **468**, 143508 (2023).

6 Tan, K.-K., Zhang, D.-W., Zhao, W.-L., Li, M. & Chen, C.-F. Axially chiral TADF-active materials with π-extended acceptors for highly efficient circularly polarized electroluminescence. *Chem. Eng. J.* **462**, 142123 (2023).

7 Qu, C. et al. Helically Chiral Donor-Acceptor Double Hetero[4]helicenes with Circularly Polarized Thermally Activated Delayed Fluorescence. *Adv. Opt. Mater.* **11**, 2203030 (2023).

8 Liao, X.-J. et al. Planar Chiral Multiple Resonance Thermally Activated Delayed Fluorescence Materials for Efficient Circularly Polarized Electroluminescence. *Angew. Chem. Int. Ed.* **62**, e202217045 (2023).

9 Zhou, Y.-H. et al. Circularly polarised photoluminescence and electroluminescence of chiral copper(i) dimers based on R/S-2,2'-bis(diphenylphosphino)-1,1'-binaphthalene ligands. *J. Mater. Chem. C* **11**, 1329-1335 (2023).

10 Yan, Z.-P. et al. A Chiral Dual-Core Organoboron Structure Realizes Dual-Channel Enhanced Ultrapure Blue Emission and Highly Efficient Circularly Polarized Electroluminescence. *Adv. Mater.* **34**, 2204253 (2022).

11 Yang, S.-Y. et al. Highly Efficient Sky-Blue π-Stacked Thermally Activated Delayed Fluorescence Emitter with Multi-Stimulus Response Properties. *Angew. Chem. Int. Ed.* **61**, e202206861 (2022).

12 Yang, Y. et al. Chiral Multi-Resonance TADF Emitters Exhibiting Narrowband Circularly Polarized Electroluminescence with an EQE of 37.2 %. *Angew. Chem. Int. Ed.* **61**, e202202227 (2022).

13 Zhang, Y.-P. et al. Efficient circularly polarized photoluminescence and electroluminescence of chiral spiro-skeleton based thermally activated delayed fluorescence molecules. *Sci. China Chem.* **65**, 1347-1355 (2022).

14 Zhang, Y.-P. et al. Circularly Polarized White Organic Light-Emitting Diodes Based on Spiro-Type Thermally Activated Delayed Fluorescence Materials. *Angew. Chem. Int. Ed.* **61**, e202200290 (2022).

15 Yang, S.-Y. et al. Efficient circularly polarized thermally activated delayed fluorescence hetero-[4]helicene with carbonyl-/sulfone-bridged triarylamine structures. *J. Mater. Chem. C* **10**, 4393-4401 (2022).

16 Song, J. et al. Highly Phosphorescent Planar Chirality by Bridging Two Square-Planar

Platinum(II) Complexes: Chirality Induction and Circularly Polarized Luminescence. *J. Am. Chem. Soc.* **144**, 2233-2244 (2022).
17 Wang, Y.-F., Liu, X., Zhu, Y., Li, M. & Chen, C.-F. Aromatic-imide-based TADF enantiomers for efficient circularly polarized electroluminescence. *J. Mater. Chem. C* **10**, 4805-4812 (2022).
18 Wu, X. et al. Fabrication of Circularly Polarized MR-TADF Emitters with Asymmetrical Peripheral-Lock Enhancing Helical B/N-Doped Nanographenes. *Adv. Mater.* **34**, 2105080 (2022).
19 Zhang, Y.-P. et al. Chiral Spiro-Axis Induced Blue Thermally Activated Delayed Fluorescence Material for Efficient Circularly Polarized OLEDs with Low Efficiency Roll-Off. *Angew. Chem. Int. Ed.* **60**, 8435-8440 (2021).
20 Ni, F. et al. Integrating molecular rigidity and chirality into thermally activated delayed fluorescence emitters for highly efficient sky-blue and orange circularly polarized electroluminescence. *Mater. Horiz.* **8**, 547-555 (2021).
21 Xu, Y., Wang, Q., Cai, X., Li, C. & Wang, Y. Highly Efficient Electroluminescence from Narrowband Green Circularly Polarized Multiple Resonance Thermally Activated Delayed Fluorescence Enantiomers. *Adv. Mater.* **33**, 2100652 (2021).
22 Yan, Z.-P. et al. Chiral Thermally Activated Delayed Fluorescence Materials Based on R/S-N2,N2′-Diphenyl-[1,1’-binaphthalene]-2,2’-diamine Donor with Narrow Emission Spectra for Highly Efficient Circularly Polarized Electroluminescence. *Adv. Funct. Mater.* **31**, 2103875 (2021).
23 Tu, Z.-L. et al. Blue Axially Chiral Biphenyl Based Thermally Activated Delayed Fluorescence Materials for Efficient Circularly Polarized OLEDs. *Adv. Opt. Mater.* **9**, 2100596 (2021).
24 Li, M., Wang, M.-Y., Wang, Y.-F., Feng, L. & Chen, C.-F. High-Efficiency Circularly Polarized Electroluminescence from TADF-Sensitized Fluorescent Enantiomers. *Angew. Chem. Int. Ed.* **60**, 20728-20733 (2021).
25 Lu, G. et al. Semitransparent Circularly Polarized Phosphorescent Organic Light-Emitting Diodes with External Quantum Efficiency over 30% and Dissymmetry Factor Close to 10−2. *Adv. Funct. Mater.* **31**, 2102898 (2021).
26 Wang, Y.-F. et al. Chiral TADF-Active Polymers for High-Efficiency Circularly Polarized Organic Light-Emitting Diodes. *Angew. Chem. Int. Ed.* **60**, 23619-23624 (2021).
27 Tu, Z.-L. et al. Axially Chiral Biphenyl Compound-Based Thermally Activated Delayed Fluorescent Materials for High-Performance Circularly Polarized Organic Light-Emitting Diodes. *Adv. Sci.* **7**, 2000804 (2020).
28 Yang, S.-Y. et al. Circularly Polarized Thermally Activated Delayed Fluorescence Emitters in Through-Space Charge Transfer on Asymmetric Spiro Skeletons. *J. Am. Chem. Soc.* **142**, 17756-17765 (2020).
29 Frédéric, L. et al. Maximizing Chiral Perturbation on Thermally Activated Delayed Fluorescence Emitters and Elaboration of the First Top-Emission Circularly Polarized OLED. *Adv. Funct. Mater.* **30**, 2004838 (2020).
30 Yan, Z.-P. et al. Configurationally Stable Platinahelicene Enantiomers for Efficient Circularly Polarized Phosphorescent Organic Light-Emitting Diodes. *Chem. Eur. J.* **25**, 5672-5676 (2019).

31 Wang, Y. et al. Circularly Polarized Electroluminescence of Thermally Activated Delayed Fluorescence-Active Chiral Binaphthyl-Based Luminogens. *ACS Applied Materials & Interfaces* **11**, 26165-26173 (2019).

32 Wu, Z.-G. et al. Chiral Octahydro-Binaphthol Compound-Based Thermally Activated Delayed Fluorescence Materials for Circularly Polarized Electroluminescence with Superior EQE of 32.6% and Extremely Low Efficiency Roll-Off. *Adv. Mater.* **31**, 1900524 (2019).

33 Wu, Z.-G. et al. Non-doped and doped circularly polarized organic light-emitting diodes with high performances based on chiral octahydro-binaphthyl delayed fluorescent luminophores. *J. Mater. Chem. C* **7**, 7045-7052 (2019).

34 Sun, S. et al. Thermally activated delayed fluorescence enantiomers for solution-processed circularly polarized electroluminescence. *J. Mater. Chem. C* **7**, 14511-14516 (2019).

35 Li, M. et al. Stable Enantiomers Displaying Thermally Activated Delayed Fluorescence: Efficient OLEDs with Circularly Polarized Electroluminescence. *Angew. Chem. Int. Ed.* **57**, 2889-2893 (2018).

36 Wang, Y.-F., Li, M., Teng, J.-M., Zhou, H.-Y. & Chen, C.-F. High-Performance Solution-Processed Nondoped Circularly Polarized OLEDs with Chiral Triptycene Scaffold-Based TADF Emitters Realizing Over 20% External Quantum Efficiency. *Adv. Funct. Mater.* **31**, 2106418 (2021).

37 Li, M., Wang, Y.-F., Zhang, D., Duan, L. & Chen, C.-F. Axially Chiral TADF-Active Enantiomers Designed for Efficient Blue Circularly Polarized Electroluminescence. *Angew. Chem. Int. Ed.* **59**, 3500-3504 (2020).

38 Song, F. et al. Highly Efficient Circularly Polarized Electroluminescence from Aggregation-Induced Emission Luminogens with Amplified Chirality and Delayed Fluorescence. *Adv. Funct. Mater.* **28**, 1800051 (2018).
