# Supplementary material for: Deuteration promotes circularly polarized light emission by suppression of vibration
Source: Nat Commun. 2025 Dec 18;17:678. doi: 10.1038/s41467-025-67342-y (PMC12820309; doi:10.1038/s41467-025-67342-y)
Supplement: Supplementary file 2 — Description Of Additional Supplementary File [file 41467_2025_67342_MOESM2_ESM.pdf]

Description of Additional supplementary files

Supplementary Data 1:

CheckCIF reports for each crystal
