# Supplementary material for: Deuteration promotes circularly polarized light emission by suppression of vibration
Source: Nat Commun. 2025 Dec 18;17:678. doi: 10.1038/s41467-025-67342-y (PMC12820309; doi:10.1038/s41467-025-67342-y)

checkCIF/PLATON report

Structure factors have been supplied for datablock(s) 2267192

THIS REPORT IS FOR GUIDANCE ONLY. IF USED AS PART OF A REVIEW PROCEDURE FOR PUBLICATION, IT SHOULD NOT REPLACE THE EXPERTISE OF AN EXPERIENCED CRYSTALLOGRAPHIC REFEREE.

No syntax errors found. CIF dictionary Interpreting this report

Datablock: 2267192

```
Bond precision:   C-C = 0.0032 A          Wavelength=1.54178

Cell:              a=7.8317(2)      b=9.5762(2)      c=36.7910(8)
                   alpha=90         beta=90          gamma=90
Temperature:       170 K

                Calculated                   Reported
Volume          2759.25(11)                  2759.25(11)
Space group     P 21 21 21                   P 21 21 21
Hall group      P 2ac 2ab                    P 2ac 2ab
Moiety formula  C38 H22 N4                   C38 H22 N4
Sum formula     C38 H22 N4                   C38 H22 N4
Mr              534.60                       534.59
Dx,g cm-3       1.287                        1.287
Z               4                            4
Mu (mm-1)       0.598                        0.598
F000            1112.0                       1112.0
F000'           1115.04
h,k,lmax        9,11,44                      9,11,44
Nref            5057[ 2921]                  5053
Tmin,Tmax       0.812,0.965                  0.650,0.753
Tmin'           0.750

Correction method= # Reported T Limits: Tmin=0.650 Tmax=0.753
AbsCorr = MULTI-SCAN

Data completeness= 1.73/1.00       Theta(max)= 68.260

R(reflections)= 0.0343( 4707)                          wR2(reflections)=
                                                       0.0820( 5053)
S = 1.106                  Npar= 379
```

```
The following ALERTS were generated. Each ALERT has the format
       test-name_ALERT_alert-type_alert-level.
Click on the hyperlinks for more details of the test.
```

Alert level G

```
PLAT432_ALERT_2_G Short Inter X...Y Contact  N2       ..C16      .       2.98 Ang.
                                           1/2+x,3/2-y,1-z  =      4_566 Check
PLAT883_ALERT_1_G No Info/Value for _atom_sites_solution_primary .     Please Do !
PLAT978_ALERT_2_G Number C-C Bonds with Positive Residual Density.          0 Info
```

```
   0 ALERT level A = Most likely a serious problem - resolve or explain
   0 ALERT level B = A potentially serious problem, consider carefully
   0 ALERT level C = Check. Ensure it is not caused by an omission or oversight
   3 ALERT level G = General information/check it is not something unexpected

   1 ALERT type 1 CIF construction/syntax error, inconsistent or missing data
   2 ALERT type 2 Indicator that the structure model may be wrong or deficient
   0 ALERT type 3 Indicator that the structure quality may be low
   0 ALERT type 4 Improvement, methodology, query or suggestion
   0 ALERT type 5 Informative message, check
```

It is advisable to attempt to resolve as many as possible of the alerts in all categories. Often the minor alerts point to easily fixed oversights, errors and omissions in your CIF or refinement strategy, so attention to these fine details can be worthwhile. In order to resolve some of the more serious problems it may be necessary to carry out additional measurements or structure refinements. However, the purpose of your study may justify the reported deviations and the more serious of these should normally be commented upon in the discussion or experimental section of a paper or in the "special_details" fields of the CIF. checkCIF was carefully designed to identify outliers and unusual parameters, but every test has its limitations and alerts that are not important in a particular case may appear. Conversely, the absence of alerts does not guarantee there are no aspects of the results needing attention. It is up to the individual to critically assess their own results and, if necessary, seek expert advice.

Publication of your CIF in IUCr journals

A basic structural check has been run on your CIF. These basic checks will be run on all CIFs submitted for publication in IUCr journals (*Acta Crystallographica*, *Journal of Applied Crystallography*, *Journal of Synchrotron Radiation*); however, if you intend to submit to *Acta Crystallographica Section C* or *E* or *IUCrData*, you should make sure that full publication checks are run on the final version of your CIF prior to submission.

Publication of your CIF in other journals

Please refer to the *Notes for Authors* of the relevant journal for any special instructions relating to CIF submission.

PLATON version of 10/05/2023; check.def file version of 10/05/2023

Datablock 230526_czd_peak2_0519 - ellipsoid plot

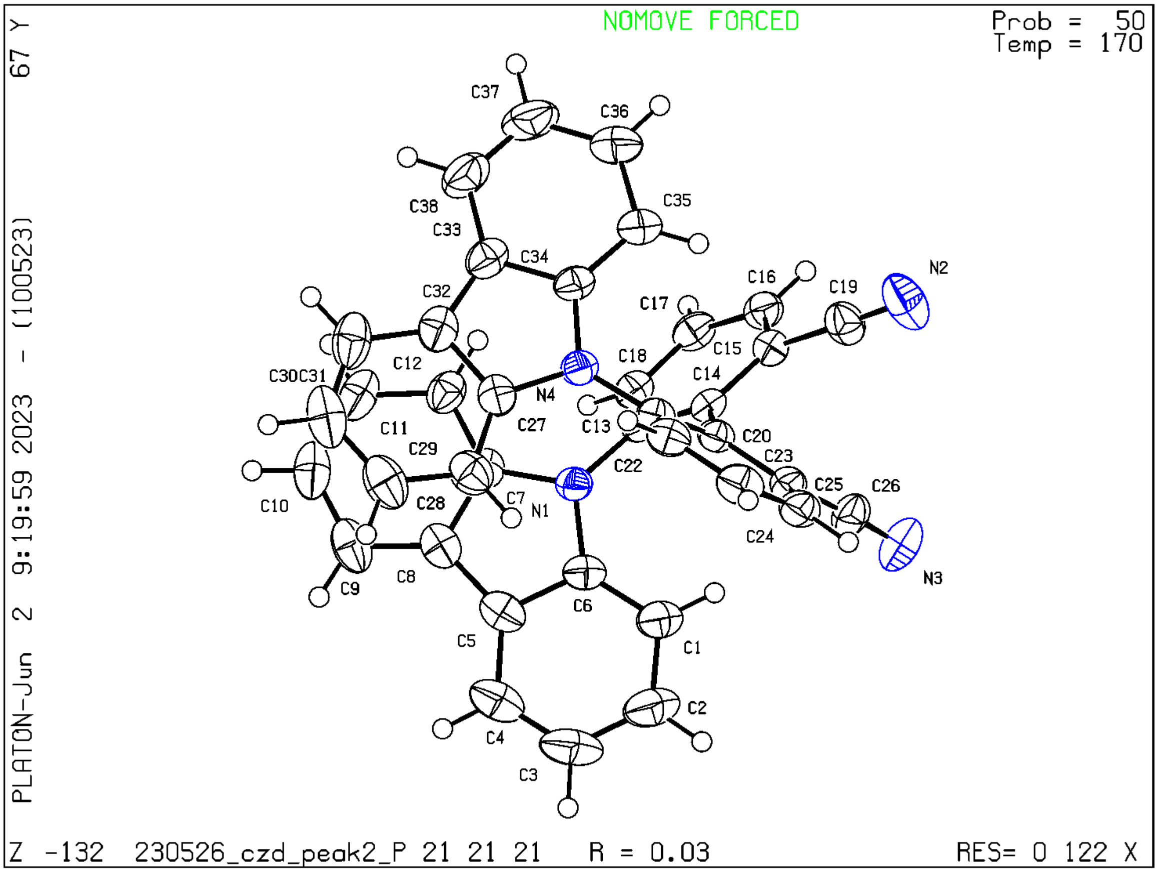

checkCIF/PLATON report

Structure factors have been supplied for datablock(s) 2285327

THIS REPORT IS FOR GUIDANCE ONLY. IF USED AS PART OF A REVIEW PROCEDURE FOR PUBLICATION, IT SHOULD NOT REPLACE THE EXPERTISE OF AN EXPERIENCED CRYSTALLOGRAPHIC REFEREE.

No syntax errors found. CIF dictionary Interpreting this report

Datablock: 2285327

Bond precision: C-C = 0.0030 A Wavelength=1.54178

Cell:	a=7.8325(2)	b=9.5739(3)	c=36.7607(11)
	alpha=90	beta=90	gamma=90
Temperature:	170 K		

	Calculated	Reported
Volume	2756.60(14)	2756.60(14)
Space group	P 21 21 21	P 21 21 21
Hall group	P 2ac 2ab	P 2ac 2ab
Moiety formula	C38 H22 N4	C38 H22 N4
Sum formula	C38 H22 N4	C38 H22 N4
Mr	534.60	534.59
Dx,g cm-3	1.288	1.288
Z	4	4
Mu (mm-1)	0.598	0.598
F000	1112.0	1112.0
F000'	1115.04	
h,k,lmax	9,11,44	9,11,44
Nref	5068[2927]	5048
Tmin,Tmax	0.778,0.871	0.658,0.753
Tmin'	0.750	

Correction method= # Reported T Limits: Tmin=0.658 Tmax=0.753
AbsCorr = MULTI-SCAN

Data completeness= 1.72/1.00 Theta(max)= 68.465

R(reflections)= 0.0315(4845) wR2(reflections)= 0.0757(5048)

S = 1.077 Npar= 379

The following ALERTS were generated. Each ALERT has the format
test-name_ALERT_alert-type_alert-level.
Click on the hyperlinks for more details of the test.

Alert level C

```
PLAT911_ALERT_3_C Missing FCF Refl Between Thmin & STh/L=    0.600         12 Report
```

Alert level G

```
PLAT432_ALERT_2_G Short Inter X...Y Contact  N1       ..C17       .       2.98 Ang.
                                          -1/2+x,1/2-y,1-z  =      4_456 Check
PLAT912_ALERT_4_G Missing # of FCF Reflections Above STh/L=  0.600          2 Note
PLAT933_ALERT_2_G Number of HKL-OMIT Records in Embedded .res File         10 Note
PLAT978_ALERT_2_G Number C-C Bonds with Positive Residual Density.          1 Info
```

0 **ALERT level A** = Most likely a serious problem - resolve or explain
0 **ALERT level B** = A potentially serious problem, consider carefully
1 **ALERT level C** = Check. Ensure it is not caused by an omission or oversight
4 **ALERT level G** = General information/check it is not something unexpected

0 ALERT type 1 CIF construction/syntax error, inconsistent or missing data
3 ALERT type 2 Indicator that the structure model may be wrong or deficient
1 ALERT type 3 Indicator that the structure quality may be low
1 ALERT type 4 Improvement, methodology, query or suggestion
0 ALERT type 5 Informative message, check

It is advisable to attempt to resolve as many as possible of the alerts in all categories. Often the minor alerts point to easily fixed oversights, errors and omissions in your CIF or refinement strategy, so attention to these fine details can be worthwhile. In order to resolve some of the more serious problems it may be necessary to carry out additional measurements or structure refinements. However, the purpose of your study may justify the reported deviations and the more serious of these should normally be commented upon in the discussion or experimental section of a paper or in the "special_details" fields of the CIF. checkCIF was carefully designed to identify outliers and unusual parameters, but every test has its limitations and alerts that are not important in a particular case may appear. Conversely, the absence of alerts does not guarantee there are no aspects of the results needing attention. It is up to the individual to critically assess their own results and, if necessary, seek expert advice.

Publication of your CIF in IUCr journals

A basic structural check has been run on your CIF. These basic checks will be run on all CIFs submitted for publication in IUCr journals (*Acta Crystallographica*, *Journal of Applied Crystallography*, *Journal of Synchrotron Radiation*); however, if you intend to submit to *Acta Crystallographica Section C* or *E* or *IUCrData*, you should make sure that full publication checks are run on the final version of your CIF prior to submission.

Publication of your CIF in other journals

Please refer to the *Notes for Authors* of the relevant journal for any special instructions relating to CIF submission.

PLATON version of 06/07/2023; check.def file version of 30/06/2023

Datablock cu_230717_czd_peak1_0708_0m - ellipsoid plot

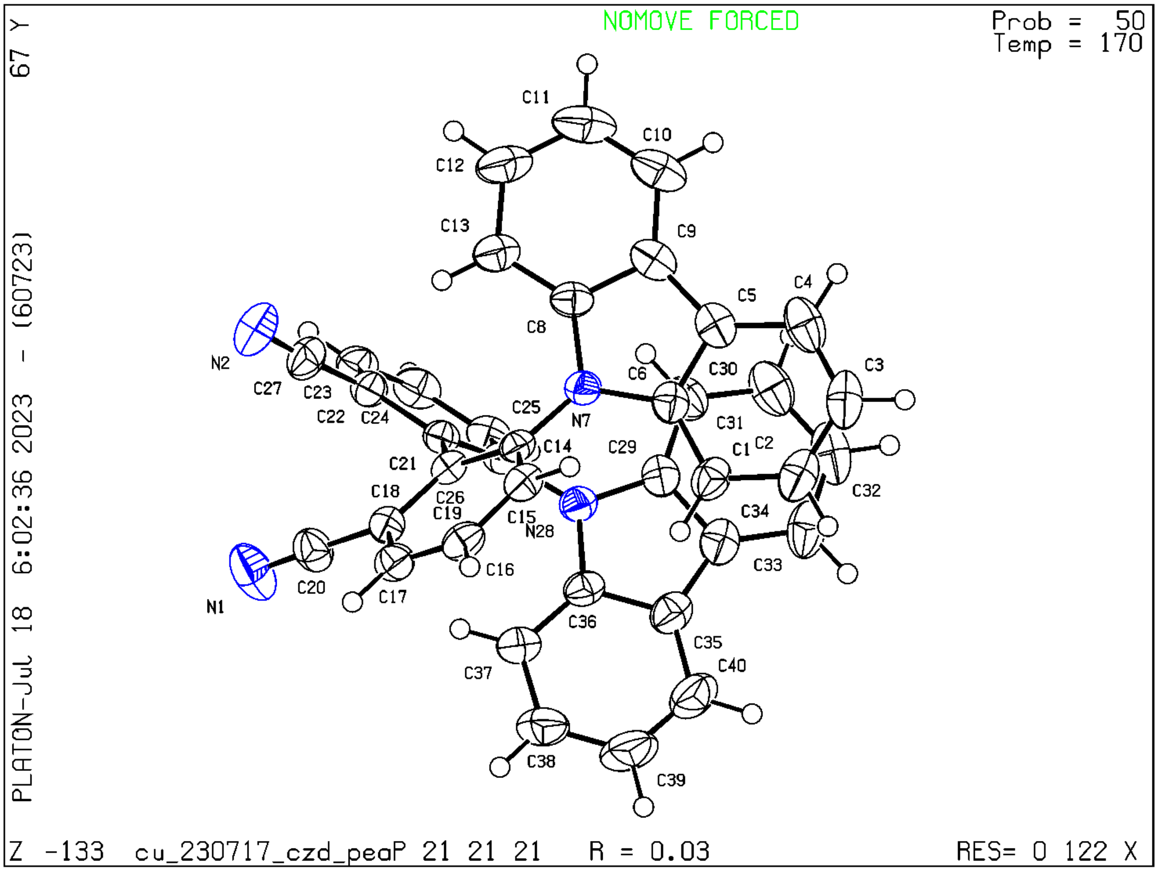

checkCIF/PLATON report

You have not supplied any structure factors. As a result the full set of tests cannot be run.

THIS REPORT IS FOR GUIDANCE ONLY. IF USED AS PART OF A REVIEW PROCEDURE FOR PUBLICATION, IT SHOULD NOT REPLACE THE EXPERTISE OF AN EXPERIENCED CRYSTALLOGRAPHIC REFEREE.

No syntax errors found. CIF dictionary Interpreting this report

Datablock: 2280086

```
Bond precision:   C-C = 0.0079 A           Wavelength=1.54184

Cell:          a=8.0571(3)        b=10.4854(5)      c=10.7967(5)
               alpha=114.097(4)   beta=96.294(3)    gamma=105.178(3)
Temperature:   104 K

                Calculated                  Reported
Volume          778.95(7)                   778.95(6)
Space group     P 1                         P 1
Hall group      P 1                         P 1
Moiety formula  C42 H30 N4                  C42 H30 N4
Sum formula     C42 H30 N4                  C42 H30 N4
Mr              590.70                      590.70
Dx,g cm-3       1.259                       1.259
Z               1                           1
Mu (mm-1)       0.576                       0.576
F000            310.0                       310.0
F000'           310.83
h,k,lmax        9,12,12                     9,12,12
Nref            5514[ 2757]                 4600
Tmin,Tmax       0.920,0.955                 0.526,1.000
Tmin'           0.912

Correction method= # Reported T Limits: Tmin=0.526 Tmax=1.000
AbsCorr = MULTI-SCAN

Data completeness= 1.67/0.83       Theta(max)= 66.593

R(reflections)= 0.0668( 4106)                          wR2(reflections)=
                                                       0.1701( 4600)
S = 1.073                  Npar= 419
```

The following ALERTS were generated. Each ALERT has the format
test-name_ALERT_alert-type_alert-level.
Click on the hyperlinks for more details of the test.

Alert level C

STRVA01_ALERT_4_C Flack test results are meaningless.
From the CIF: _refine_ls_abs_structure_Flack 0.000
From the CIF: _refine_ls_abs_structure_Flack_su 0.600
PLAT089_ALERT_3_C Poor Data / Parameter Ratio (Zmax < 18) .... 6.58 Note
PLAT340_ALERT_3_C Low Bond Precision on C-C Bonds .... 0.00787 Ang.

Alert level G

PLAT012_ALERT_1_G N.O.K. _shelx_res_checksum Found in CIF ...... Please Check
PLAT032_ALERT_4_G Std. Uncertainty on Flack Parameter Value High . 0.600 Report
PLAT072_ALERT_2_G SHELXL First Parameter in WGHT Unusually Large 0.12 Report
PLAT850_ALERT_4_G Check Flack Parameter Exact Value 0.00 with s.u. 0.60 Check
PLAT933_ALERT_2_G Number of HKL-OMIT Records in Embedded .res File 3 Note
PLAT941_ALERT_3_G Average HKL Measurement Multiplicity .... 3.9 Low

---

0 **ALERT level A** = Most likely a serious problem - resolve or explain
0 **ALERT level B** = A potentially serious problem, consider carefully
3 **ALERT level C** = Check. Ensure it is not caused by an omission or oversight
6 **ALERT level G** = General information/check it is not something unexpected

1 ALERT type 1 CIF construction/syntax error, inconsistent or missing data
2 ALERT type 2 Indicator that the structure model may be wrong or deficient
3 ALERT type 3 Indicator that the structure quality may be low
3 ALERT type 4 Improvement, methodology, query or suggestion
0 ALERT type 5 Informative message, check

It is advisable to attempt to resolve as many as possible of the alerts in all categories. Often the minor alerts point to easily fixed oversights, errors and omissions in your CIF or refinement strategy, so attention to these fine details can be worthwhile. In order to resolve some of the more serious problems it may be necessary to carry out additional measurements or structure refinements. However, the purpose of your study may justify the reported deviations and the more serious of these should normally be commented upon in the discussion or experimental section of a paper or in the "special_details" fields of the CIF. checkCIF was carefully designed to identify outliers and unusual parameters, but every test has its limitations and alerts that are not important in a particular case may appear. Conversely, the absence of alerts does not guarantee there are no aspects of the results needing attention. It is up to the individual to critically assess their own results and, if necessary, seek expert advice.

Publication of your CIF in IUCr journals

A basic structural check has been run on your CIF. These basic checks will be run on all CIFs submitted for publication in IUCr journals (*Acta Crystallographica*, *Journal of Applied Crystallography*, *Journal of Synchrotron Radiation*); however, if you intend to submit to *Acta Crystallographica Section C* or *E* or *IUCrData*, you should make sure that full publication checks are run on the final version of your CIF prior to submission.

Publication of your CIF in other journals

Please refer to the *Notes for Authors* of the relevant journal for any special instructions relating to CIF submission.

PLATON version of 10/05/2023; check.def file version of 10/05/2023

Datablock peak1-0622 - ellipsoid plot

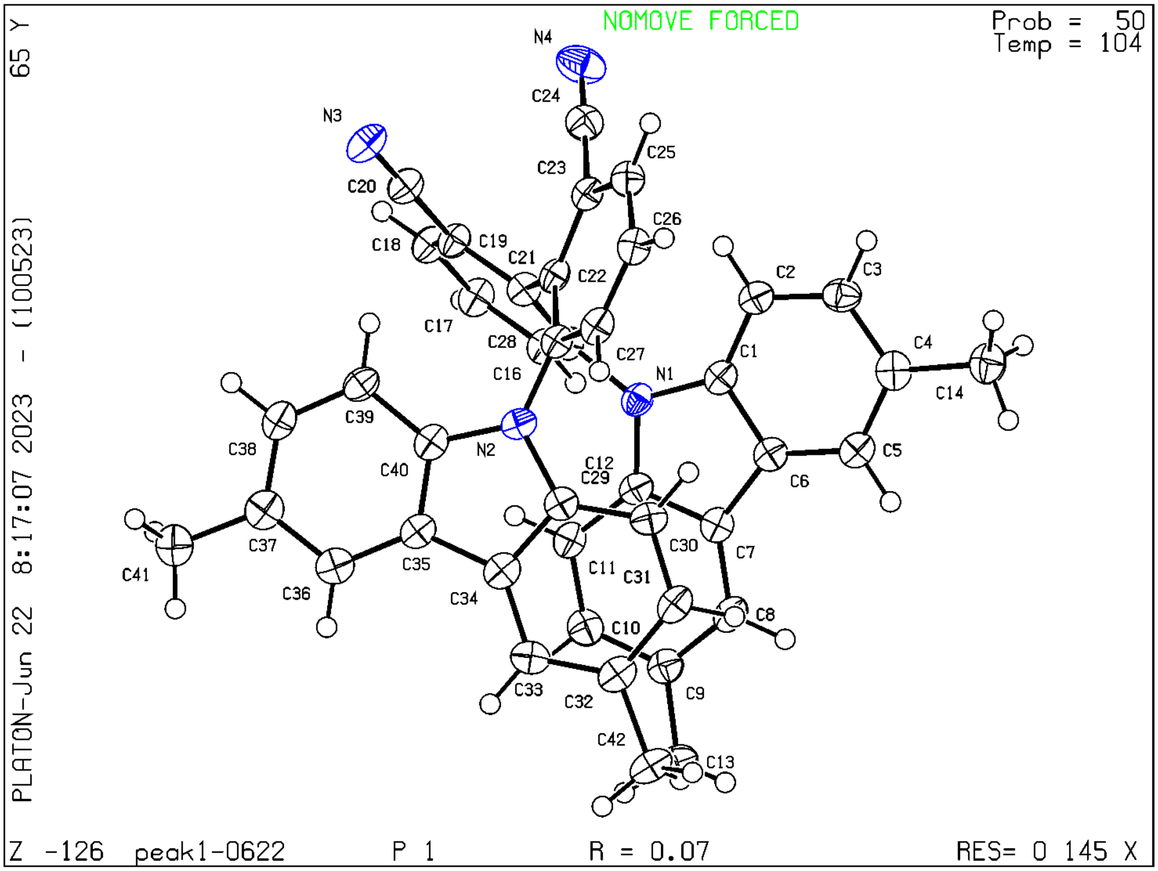

checkCIF/PLATON report

Structure factors have been supplied for datablock(s) 2280087

THIS REPORT IS FOR GUIDANCE ONLY. IF USED AS PART OF A REVIEW PROCEDURE FOR PUBLICATION, IT SHOULD NOT REPLACE THE EXPERTISE OF AN EXPERIENCED CRYSTALLOGRAPHIC REFEREE.

No syntax errors found. CIF dictionary Interpreting this report

Datablock: 2280087

```
Bond precision:   C-C = 0.0028 A           Wavelength=1.54184

Cell:          a=8.06502(14)      b=10.4954(3)       c=10.7929(3)
               alpha=114.135(3)   beta=96.2062(17)   gamma=105.2119(19)
Temperature:   100 K

                 Calculated                    Reported
Volume           780.06(4)                     780.07(4)
Space group      P 1                           P 1
Hall group       P 1                           P 1
Moiety formula   C42 H30 N4                    C42 H30 N4
Sum formula      C42 H30 N4                    C42 H30 N4
Mr               590.70                        590.70
Dx,g cm-3        1.258                         1.257
Z                1                             1
Mu (mm-1)        0.575                         0.575
F000             310.0                         310.0
F000'            310.83
h,k,lmax         10,13,13                      10,13,13
Nref             6346[ 3173]                   5336
Tmin,Tmax        0.933,0.955                   0.800,1.000
Tmin'            0.923

Correction method= # Reported T Limits: Tmin=0.800 Tmax=1.000
AbsCorr = MULTI-SCAN

Data completeness= 1.68/0.84        Theta(max)= 74.151

R(reflections)= 0.0262( 5285)                         wR2(reflections)=
                                                      0.0672( 5336)
S = 1.027                   Npar= 420
```

The following ALERTS were generated. Each ALERT has the format
test-name_ALERT_alert-type_alert-level.
Click on the hyperlinks for more details of the test.

Alert level C

```
PLAT089_ALERT_3_C Poor Data / Parameter Ratio (Zmax < 18) ........       7.27 Note
PLAT911_ALERT_3_C Missing FCF Refl Between Thmin & STh/L=    0.600         14 Report
PLAT915_ALERT_3_C No Flack x Check Done: Low Friedel Pair Coverage         72 %
```

Alert level G

```
PLAT230_ALERT_2_G Hirshfeld Test Diff for    C19      --C28      .        5.1 s.u.
PLAT230_ALERT_2_G Hirshfeld Test Diff for    C22      --C27      .        5.6 s.u.
PLAT912_ALERT_4_G Missing # of FCF Reflections Above STh/L=  0.600        104 Note
PLAT913_ALERT_3_G Missing # of Very Strong Reflections in FCF ....          1 Note
PLAT933_ALERT_2_G Number of HKL-OMIT Records in Embedded .res File          2 Note
PLAT941_ALERT_3_G Average HKL Measurement Multiplicity ..........         3.9 Low
PLAT978_ALERT_2_G Number C-C Bonds with Positive Residual Density.         17 Info
```

0 **ALERT level A** = Most likely a serious problem - resolve or explain
0 **ALERT level B** = A potentially serious problem, consider carefully
3 **ALERT level C** = Check. Ensure it is not caused by an omission or oversight
7 **ALERT level G** = General information/check it is not something unexpected

0 ALERT type 1 CIF construction/syntax error, inconsistent or missing data
4 ALERT type 2 Indicator that the structure model may be wrong or deficient
5 ALERT type 3 Indicator that the structure quality may be low
1 ALERT type 4 Improvement, methodology, query or suggestion
0 ALERT type 5 Informative message, check

It is advisable to attempt to resolve as many as possible of the alerts in all categories. Often the minor alerts point to easily fixed oversights, errors and omissions in your CIF or refinement strategy, so attention to these fine details can be worthwhile. In order to resolve some of the more serious problems it may be necessary to carry out additional measurements or structure refinements. However, the purpose of your study may justify the reported deviations and the more serious of these should normally be commented upon in the discussion or experimental section of a paper or in the "special_details" fields of the CIF. checkCIF was carefully designed to identify outliers and unusual parameters, but every test has its limitations and alerts that are not important in a particular case may appear. Conversely, the absence of alerts does not guarantee there are no aspects of the results needing attention. It is up to the individual to critically assess their own results and, if necessary, seek expert advice.

Publication of your CIF in IUCr journals

A basic structural check has been run on your CIF. These basic checks will be run on all CIFs submitted for publication in IUCr journals (*Acta Crystallographica*, *Journal of Applied Crystallography*, *Journal of Synchrotron Radiation*); however, if you intend to submit to *Acta Crystallographica Section C* or *E* or *IUCrData*, you should make sure that full publication checks are run on the final version of your CIF prior to submission.

Publication of your CIF in other journals

Please refer to the *Notes for Authors* of the relevant journal for any special instructions relating to CIF submission.

PLATON version of 10/05/2023; check.def file version of 10/05/2023

Datablock peak-0618 - ellipsoid plot

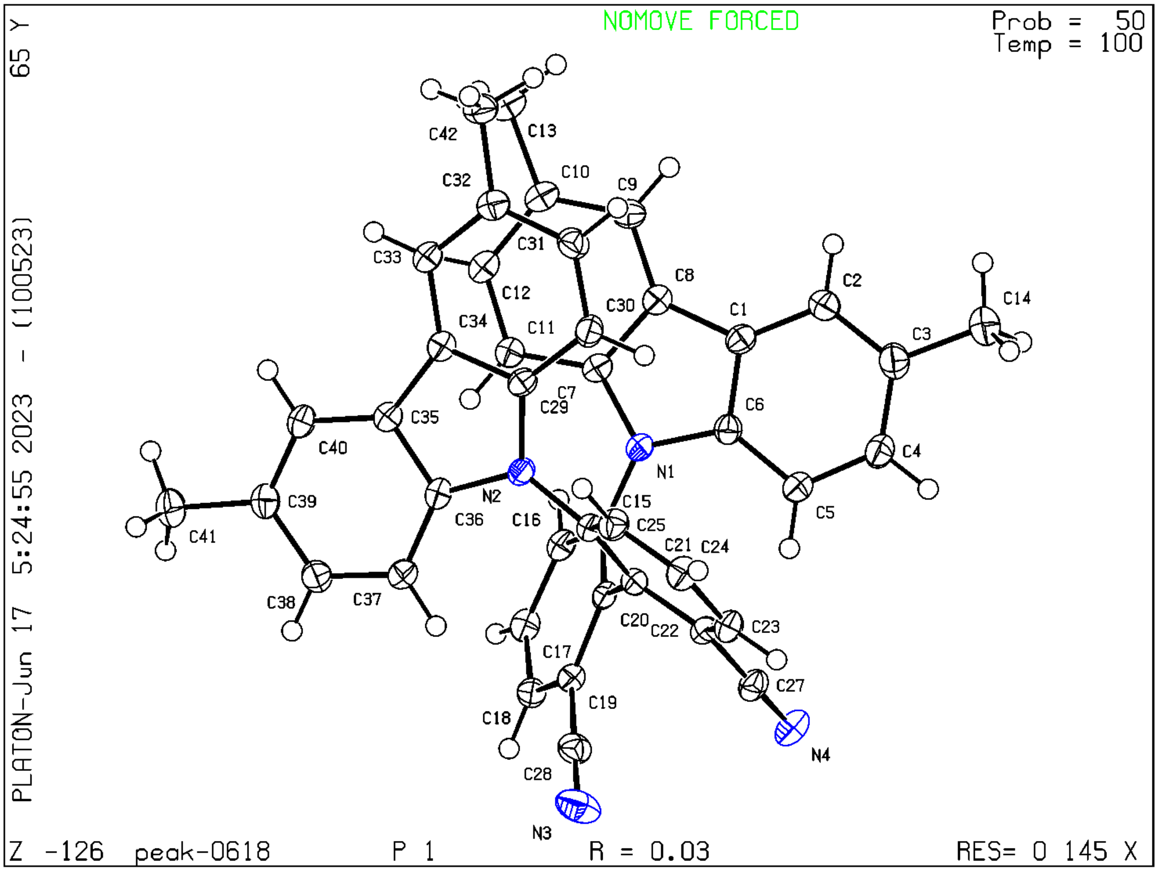

Supplement: Supplementary file 3 — Supplementary Data 1 [file 41467_2025_67342_MOESM3_ESM.pdf]
